# Embodying an artificial hand increases blood flow to the investigated limb

**DOI:** 10.12688/openreseurope.13641.1

**Published:** 2021-05-21

**Authors:** Giovanni Di Pino, Alessandro Mioli, Claudia Altamura, Marco D'Alonzo

**Affiliations:** 1 NeXT: Neurophysiology and Neuroengineering of Human-Technology Interaction Research Unit, Campus Bio-Medico University of Rome, via Alvaro del Portillo, 5, Rome, 00128, Italy; 2 Headache and Neurosonology Unit, Neurology, Campus Bio-Medico University of Rome, via Alvaro del Portillo, 200, Rome, 00128, Italy

**Keywords:** Autonomic nervous system, upper limb embodiment, blood flow, Rubber Hand Illusion paradigm, peripherical vascular resistance, brachial artery, body representation

## Abstract

**Background: **The autonomic nervous system is the main determinant of the blood flow directed towards a body part, and it is tightly connected to the representation of the body in the brain; would the experimental modulation of the sense of ownership of the limb affect its blood perfusion?

**Methods: **In healthy participants, we employed the rubber hand illusion paradigm to modulate limb ownership while we monitored the brachial artery blood flow and resistance of the investigated limb.

**Results: **In all conditions with brush-stroking, we found an initial drop in the blood flow due to tactile stimulation. Subsequently, in the illusion condition where both the rubber and real hand experience synchronous brush-stroking, the blood flow rose significantly faster and reached significantly higher values. Moreover, the increase in blood flow correlated to the embodiment level measured by questionnaires and, negatively, to the change of peripherical vascular resistance.

**Conclusions: **These findings demonstrate that modulating the representation of a body part impacts its blood perfusion.

## Plain language summary

The autonomic nervous system controls the visceral body and its blood perfusion, by adapting it to our behavior. Its activity is influenced by cognitive and emotional processes, it is bidirectionally connected to the network hosting the body representation, to the creation of which it contributes. By exploiting brachial artery blood flow recording during the rubber hand illusion paradigm, we demonstrated that modulating the belonging of a body part to the body representation increases its perfusion, through a sympathetic-driven downstream vasodilatation. The blood flow increase correlated to the achieved level of fake hand embodiment. This raises intriguing questions on the local specificity of the blood flow enhancement, and on the essence of its causal connection with the alteration of the sense of embodiment of the limbs.

## Introduction

The autonomic nervous system (ANS) takes care of the involuntary control of the visceral body. Glands, smooth and cardiac muscles are regulated to maintain the body homeostasis and to adapt the digestion, body temperature, ventilation, cardiac activity and regional blood flow to our behavior.

Despite the ANS being mainly a low-level control system, it is strongly influenced by emotive and cognitive processes. Depending on emotions and feelings, connections between the amygdala and the medial cortices (anterior cingulate, insular, and ventromedial prefrontal cortex) in association with the dorsal pons and hypothalamus, modulate blood pressure, pupil size, heart rate and electrodermal activity ^1^. Moreover, ANS homeostatic information related to pain, temperature, pH, carbon dioxide, and oxygen are sent to the insula and interact with somatosensory processing to build the body representation ^2^.

Pathways and cortical elaboration centers of interoceptive and exteroceptive information often overlap. For example, somatosensori-motor cortices, extra-striate body area and the dorsal precuneus control gastric activity, digestion, cardiac output and heart rate, and they are also involved in mapping bodily space through touch, action and vision. In particular, the primary sensorimotor cortex receives both tactile and visceral afferents combining internal and external bodily information ^3– 6^.

Evidence of the tight connection between the ANS and central body representation derives from complex regional pain syndrome (CRPS) ^7^, where the alteration of the brain representation of a body part impacts on the autonomic neural pathway subserving that part. In CRPS, an autonomic dysfunction results in changes to the skin blood flow, warmer limbs, change of colour, edema, longer nails and abnormal sudomotor activity ^8^. CRPS is usually triggered by a limb-related trauma and a subsequent period of immobilization. The associated pain is related to sympathetic hyperactivity as well, so patients benefit from early sympathetic blockade ^9^. The strange association of a ‘neglect-like’ syndrome ^10^ with an over-representation of the affected hemispace ^11^, and of an enlargement of the affected limb motor cortex ^12^ with a reduction in its primary sensory cortex ^13^ could imply that the derangement of body representation affects CRPS pathogenesis. Moreover, both pain and autonomic symptoms are relieved with interventions manipulating the representation of the limb, such as mirror therapy ^14^; minimizing lens ^15^; or prism adaptation ^7, 11^.

Emerging evidence for the existence of a strong relationship between body representation and interoceptive signals are not confined to pathological models, but can be also gathered in the normal context from studies on healthy participants. For example, interoceptive information such as cardiac feedback can modulate the visual body perception ^16^ and influence one’s own body awareness ^17, 18^ or, vice-versa, changes in body-ownership and self-identification can alter the ability to detect internal body signals ^19^. Furthermore, interoceptive sensitivity predicts the malleability of participants’ body representation ^20^.

ANS is in charge of blood perfusion; think for instance to the viscera-to-muscle redirection of the flow during the fight or flight response, to the reduction of wound hemorrhages, thermoregulation and thermomimesis ^21^. For these responses, the nucleus tractus solitarius integrates signals from the periphery and from higher brain centers, to control vagal and sympathetic outflow ^22^. Preoptic hypothalamic and forebrain centers interact with periaqueductal gray and raphe nuclei ^23^ when the limb flow is modulated by cognitive and emotional processes, the level of attention ^24^, and anxiety ^25– 27^. The amygdala, involved in vigilance and arousal, and the habenula, activated by aversive events or missing rewards, control vasoconstriction triggered by salient alerting stimuli ^28^.

Hitherto, we know that i) the central ANS is tightly connected with circuits subserving the representation of the body, ii) cognitive processes influence central ANS control of the local blood flow, and that iii) a syndrome due to an alteration of the limb representation (i.e. CPRS) presents an autonomic-driven dysfunction of the vascular supply to the affected limb.

Altogether, this suggests that modifying the brain representation of a body part could result in a change of the blood perfusion of that part; however, this has never been demonstrated so far.

A simple way to modulate the body representation is through the rubber hand illusion (RHI), a perceptual illusion caused by the synchronous brush-stroking of the hidden participant’s real hand and a visible fake one ^29^. Spatio-temporal congruency of visuo-tactile stimuli is mandatory for the illusion to arise and asynchronous stimuli abolish it, because the sense of body ownership depends on Bayesian integration of different information into a pre-existent internal body map ^30– 32^. Indeed, the illusion is abolished when the visual and somatosensory stimulation are presented asynchronously.

This work assessed whether modulating the belonging of the upper limb to the body representation would impact on its perfusion. In healthy subjects, we recorded the brachial artery flow of the limb involved in three different RHI conditions: synchronous *(Synch)*, asynchronous brush-stroking ( *Asynch*), and the mere sight of the fake hand while the hidden real hand was not stimulated ( *VisionOnly*).

## Methods

### Participants

The participants were selected among a population of students and collaborators of Neurophysiology and Neuroengineering of Human-Technology Interaction (NeXT) Research Unit that volunteers to participate to the study. Inclusion criteria were to be older than 18 years, to be naïve to the RHI protocol, to have normal hand sensation and normal, or corrected to normal, vision. To our best knowledge, we are the first to systematically measure blood flow on the forearm and on the hand while participants experience the rubber hand illusion and, for reason, it was not possible to calculate participants sample size with *a priori* power analysis. Therefore, in this case, the number of participants was chosen equal to previous RHI studies. Participants were enrolled after having signed a written informed consent to the participation and publication of the data, including the permission for their treatment images. The experimental procedures were approved by the Ethics Committee of the Università Campus Bio-Medico di Roma (EMBODY protocol) and carried out according to the Declaration of Helsinki and its future amendments.

### Experimental procedure

**Figure 1.**
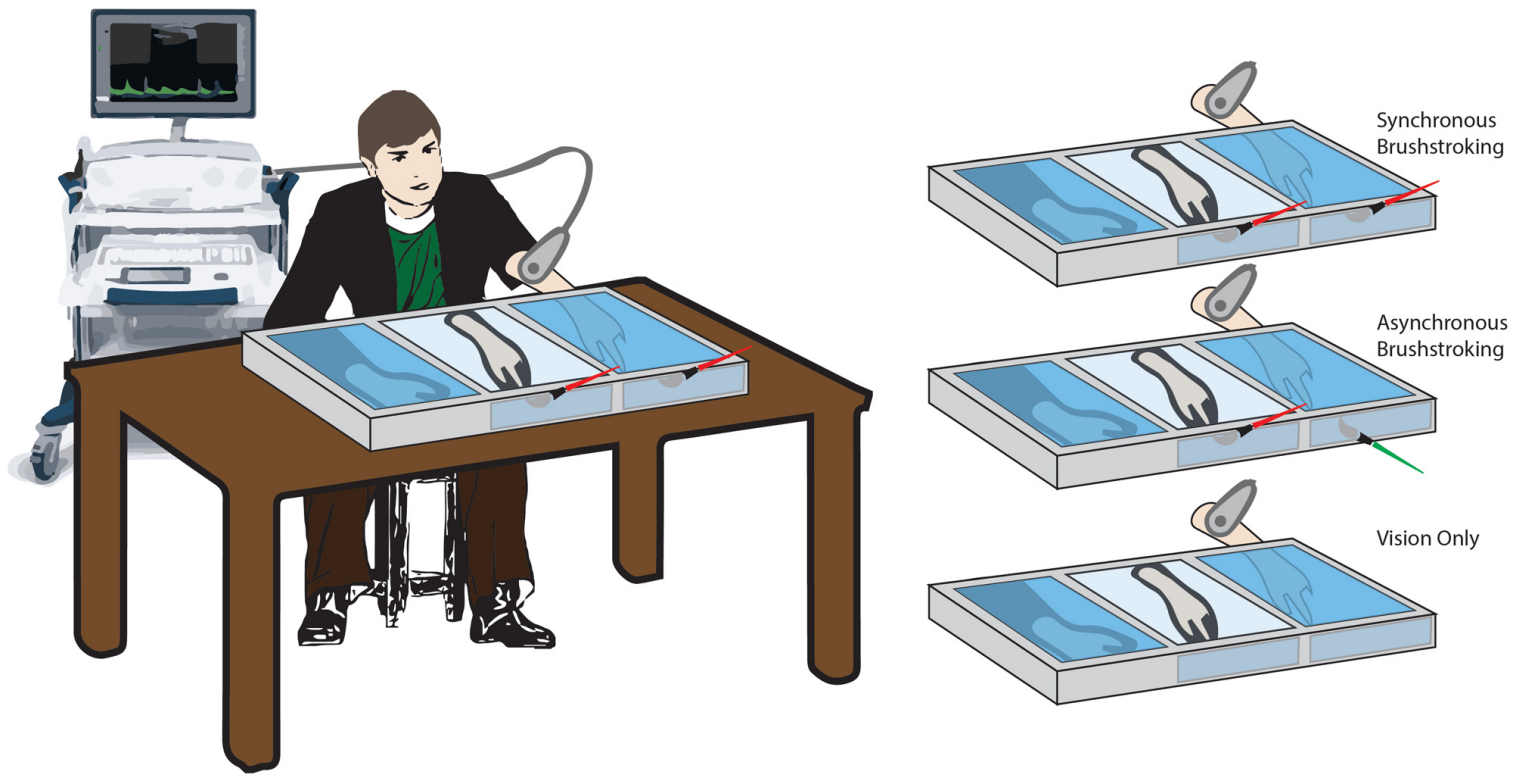
Schematic illustration of the experimental conditions. Setup and rubber hand illusion paradigm conditions.

The study was performed in a dedicated room of the NeXT Research Unit, in a period ranged between September 2018 and June 2019. Participants were placed in front of a custom-made experimental set-up, made of three parallel compartments (L × W × H = 40 × 60 × 20 cm each) covered by a two-way mirror ( Figure 1). They could see the content of each compartment only when the experimenter turned the relative internal light on 33, 34. Then, participants were invited to place their forearms inside the two lateral compartments while their shoulders were covered by a black cloak. A left rubber hand, matching the participant’s gender, was placed in the central compartment of the structure, 15cm apart from the real hidden left hand of the subject. The left hand was tested because it appears to be the side where it is easier to induce the RHI ^35^.

Three conditions were tested for each participant, administered in a random order:

- *Synchronous* ( *Synch*) *condition*: a well-trained experimenter used two identical paintbrushes to stroke both the second digit of the rubber hand and the corresponding digit on the real hidden hand. The tactile stimulation was delivered at a frequency of 1Hz. The brushstroke duration was about 0.6–0.7s, and it was delivered from the proximal to the distal phalanx.
- *Asynchronous* ( *Asynch*) *condition*: similarly, the experimenter used the paintbrushes to stroke the second digit of the participant, but a small temporal delay (about 0.5s) was added between the stimulus delivered on the rubber hand and the one delivered on the real hand ( Figure 1).
- *Vision only condition*: in this case, no stroking was delivered to either the rubber or the real hidden hand. The participant was instructed to simply look at the rubber hand for the entire duration of the condition. Such condition was performed in order to control for the effect of mere tactile stimulation on the blood flow and was considered as an additional condition of no embodiment ^36^. In such condition only the blood flow was recorded.

Each condition lasted 100s.

Blood flow was collected at a sampling rate of 100 Hz by using a Multidop-X DWL (Elektronische Systeme GmbH, Germany). The probe of the device was placed at the level of the brachial artery on the medial aspect of the tested (i.e. left) arm. We selected to employ a 4 MHz DWL ultrasound probe, which is suited to monitor the blood flow of the brachial artery considering that it can penetrate roughly 12–30 mm. The probe was kept still by the experimenter during the whole protocol. The brachial artery was selected because it is the major blood vessel located in the upper arm: the main supplier of blood to the arm and hand.

Each 1.3s, the device calculated and saved for further analysis three parameters: the mean blood flow, the peak of systolic flow and the peak of the diastolic blood flow. Once the blood flow was stable, it was recorded for 120s, from 20s before the compartment’s lighting was turned on to 100s after it. For each condition, about 92 measures of each parameter were recorded (about 77 if considering the period when compartment’s lighting was on).

### Embodiment measures

Two measures of embodiment (proprioceptive drift and nine-item self-evaluation questionnaire) were collected to assess the embodiment of the rubber hand induced by brush-stroking in synchronous and asynchronous conditions.

The proprioceptive drift was assessed ^29^ by asking the participants to verbally report a number on a measuring tape reflected on the two-way mirror that corresponded to the perceived location of their left index finger by maintaining the hands still and relaxed.

For each condition, the perceived location was collected twice, before and after brush-stroking. To guarantee a random offset before every assessment, the measuring tape had the possibility to slide. Positive differences between the hand position estimated post and pre-stimulation indicate a drift of the perceived location of the participants’ hand towards the rubber hand.

Then, the experimenter handed to the participant a nine-item questionnaire made up of three questions aimed at investigating the extent of the self-attribution of the rubber hand and six control questions testing participant susceptibility ^29^ ( Table 1). The participants were asked to rate the extent to which these items did or did not apply to them, using a seven-point scale. On this scale, -3 meant ‘‘absolutely certain that it did not apply,’’ 0 meant ‘‘uncertain whether it applied or not,’’ and +3 meant ‘‘absolutely certain that it applied’’. Such questionnaire was provided with two additional items to rate the vividness (0 - 10) of the perceived illusion (i.e. how realistic the illusion was when it was experienced) and the prevalence (0 - 100%), which reflected the percentage of time that the illusion was experienced (i.e. how long with respect to the length of section the perception of the illusion was).

**Table 1.** List of items of the questionnaire.

Questionnaire	Item	Rating
Item 1	It seemed as if I were feeling the tactile stimulation at the location where I saw the visible hand touched	-3 – +3
Item 2	It seemed as though the stimulation I felt was caused by the touch on the visible hand	
Item 3	I felt as if the visible hand was mine	
Item 4	I felt as if the position of my real hand was drifting towards the visible hand	
Item 5	It seemed as if I had more than two hand or arm	
Item 6	It seemed as if the tactile stimulation I was feeling came from somewhere between my own hand and the visible one	
Item 7	I felt as if my real hand were turning ‘rubbery’	
Item 8	It appeared as if the position of the visible hand was drifting towards my real hand	
Item 9	The visible hand began to resemble my own hand, in terms of shape, skin tone, freckles or some other visual features	
Vividness	How realistic and life-like was the illusion that the visible hand was yours when it was experienced?	0 – 10
Prevalence	How long with respect to the length of section the perception of such illusion was?	0 – 100%

The overall experimental session lasted about 30 minutes for each participant.

### Data analysis

The Kolgomorov-Smirnov test (p >0.05) was used to verify that the data relative to the typical RHI outcomes (nine-item questionnaire, vividness, prevalence score and proprioceptive drift) were normally distributed. To verify that the responses to the questionnaires were not due to the participants’ suggestibility, the mean score of the three items employed to measure the effective illusion was compared against the mean score of six items served as controls for compliance, suggestibility, and ‘‘placebo effect’’, by using a two-tailed paired t-test.

Then, on the basis of questionnaires’ responses, a single index was calculated: the RHI index, which expresses the difference between the mean score of the illusion items and the mean score of the other ones ^37^. The RHI index was calculated for each condition and considered as the “illusion outcome” in the following analyses.

Questionnaire outcomes and proprioceptive drift were analyzed with paired t-test to highlight differences between the illusion condition ( *Synch*) and the asynchronous control condition ( *Asynch*). Effect size (d) was also calculated as Cohen’s d.

Regarding the blood flow signal analysis, the mean blood flow signal ( *f*) was smoothed by using a moving average 5s window to eliminate the high frequency noise. In order to minimize the influence of inter-individual variability and of the circumstance on which the experiment was run (e.g. the room temperature), the extracted measure was expressed as percentage change with respect to a baseline value for each trial ( *F(t)*), for simplicity hereafter called mean blood flow and calculated using the following equation:

$$F(t)=100*\frac{f(t)-\bar{f}\left(\text{Δ}t_{b}\right)}{\bar{f}(\text{Δ}t_{b})}\;(1)$$

Where *f(t)* is the blood flow value at certain time t, $\bar{f}(\text{Δ}t_{b})$ is the value of baseline calculated as blood flow values averaged on the last 5s window of the baseline interval (i.e. Δt _b_ = [-5s, 0s]). These values were calculated for each condition and participant.

After that, the *F*(t) values were averaged across participants for each condition.

The statistical analysis was performed on three contiguous equally-long time-intervals, altogether lasting the entire duration of the trial: Δt _1_ = [0.01s, 33.33s], Δt _2_ = [33.34s, 66.66s] and Δt _3_ = [66.66s, 100s]. The average value of the blood flow was extracted in the different time-intervals for each condition and participant ( $\bar{F}$ (Δt _1_), $\bar{F}$ (Δt _2_) and $\bar{F}$ (Δt _3_)). After checking the normality of the data by using Kolmogorov-Smirnov test (p >0.05), a Mauchly test was employed to verify the sphericity of the distribution of the values and a repeated measures ANOVA (rmANOVA) with two factors (i.e. *condition* and *time*) was performed with Greenhouse-Geisser adjustment. Hence, a paired t-test with Tukey-Kramer adjustment was employed as post-hoc analysis. Additionally, the effect size (Cohen’s d) was calculated for each comparison.

A drop of the mean blood flow values was identified at around 10s from the beginning of the conditions. Thus, we corrected the blood curves of all the three conditions to make all of them starting from the same value after the drop, the value of the drop was subtracted to the mean blood flow by using the following equation:

$$\text{Δ}F(t)=F(t)-\bar{F}(\text{Δ}t_{d})\;(2)$$

where $\bar{F}$ *(Δt _d_)* was calculated as blood flow value averaged on a 10s window centered 10s after the beginning of the trial (i.e. the drop of the signal) (i.e. Δt _d_ = [5s, 15s]).

The obtained signal in the interval between 10s and 100s were fitted by using an exponential curve:

$$y(t)=a*(1-e^{\left(b*(t-10s)\right)})\;(3)$$

where *a* and *b* are the coefficients of the curve employed to fit the data.

*a* is the value to which the curve asymptotically tends (i.e. trend value): the higher the *a*, the higher the trend value. *b* is the rate to reach the trend value: the higher the absolute value of *b*, the faster the curve rate. *a* and *b* coefficients in the different conditions were compared using a Friedman test, and post hoc tests with Tukey-Kramer correction were employed for pairwise comparisons. Effect size (r) was calculated as z/√n, where z is test statistic for signed-rank test and n is the number of observations.

The link between blood flow changes and embodiment was investigated by correlating (Spearman’s) *a* and *b* coefficients with the illusion outcomes in *Synch* and *Asynch* condition.

The resistance index ( *ri*) ^38– 40^ was calculated as:

$$ri(t)=\frac{f_{Syst}(t)-f_{Dias}(t)}{f_{Syst}(t)}\;(4)$$

where *f _Syst_* is the systolic peak blood flow, *f _Dias_* is the diastolic blood flow. The signal of the resistance index was smoothed and normalized by using the same strategy of equation (1), the result is hereafter simply named resistance index ( *RI(t)*). The average value of the resistance index was extracted in Δt _1_, Δt _2_ and Δt _3 _time-intervals for each condition and participant ( $\bar{RI}$ *(Δt _1_)* $\bar{RI}$ *(Δt _2_)* and $\bar{RI}$ *(Δt _3_)*). Correlations (Spearman’s coefficients) between the resistance index and mean blood flow values in all conditions were calculated for all three time intervals (Δt _1_, Δt _2_ and Δt _3_).

The analysis was performed with Matlab2015a (Mathworks), a freely available alternative software is GNU Octave and JASP for statistical analysis.

## Results

Twenty volunteers took part in the experiment (age: 29.55 ± 6.12; 12 M, 8 F; 20 right-handed as by self-report).

**Figure 2.**
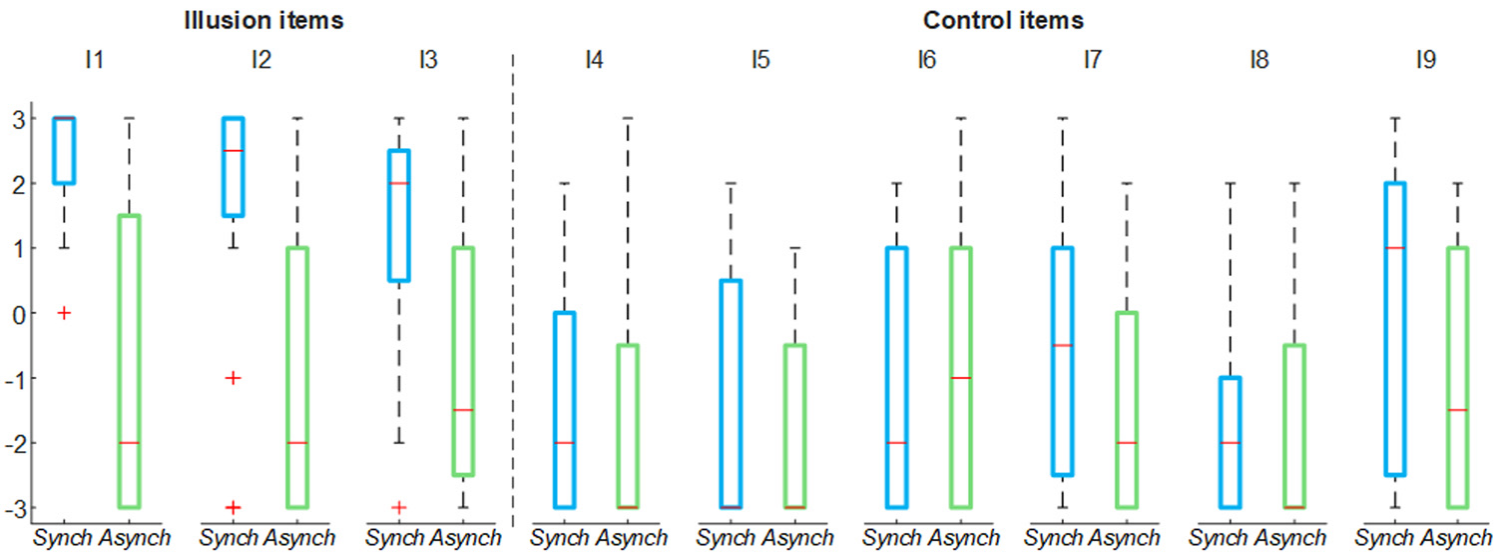
Nine-item questionnaire outcomes. Box and whisker plots of nine-item questionnaire outcomes for *Synch* and *Asynch* conditions: median (red lines), 1 ^st^ and 3 ^rd^ quartiles (box), lowest and highest values comprised within 1.5 times the interquartile range from the 1 ^st^ and 3 ^rd^ percentiles (whisker).

For both stroking ( *Synch* and *Asynch*) conditions, the mean value of the illusion items of the self-evaluation questionnaire was higher than the mean value of the control items ( *Synch*: d = 1.82; t(19) = 7.95, p <0.001; *Asynch*: d = 0.50; t(19) = 2.19, p = 0.041), thus the group of participants were generally not suggestible ( Figure 2) ^41^.

**Figure 3.**
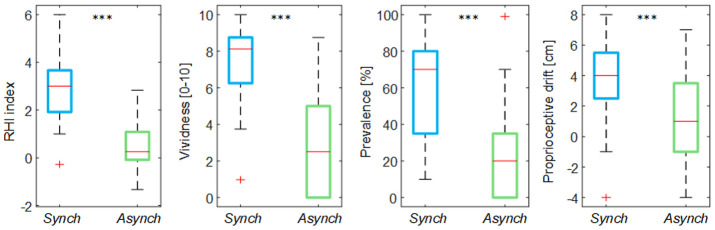
Illusion outcomes. Box and whisker plots of the illusion outcomes (rubber hand illusion [RHI] index, vividness, prevalence rating and proprioceptive drift) for *Synch* and *Asynch* conditions: median (red lines), 1 ^st ^and 3 ^rd^ quartiles (box), lowest and highest values comprised within 1.5 times the interquartile range from the 1 ^st^ and 3 ^rd^ percentiles (whisker). *** indicates a p-value <0.001.

The illusion items’ score in the *Synch* condition was significantly higher than that in the *Asynch* condition for all the embodiment measures employed (RHI index: d = 1.17; t(19) = 5.12, p <0.001; vividness: d = 1.34; t(19) = 5.84, p <0.001; prevalence: d = 1.22; t(19) = 5.31, p <0.001; proprioceptive drift: d = 0.84, t(19) = 3.68, p = 0.002) ( Figure 3). This confirms that participants effectively experienced the RHI in the *Synch* condition.

**Figure 4.**
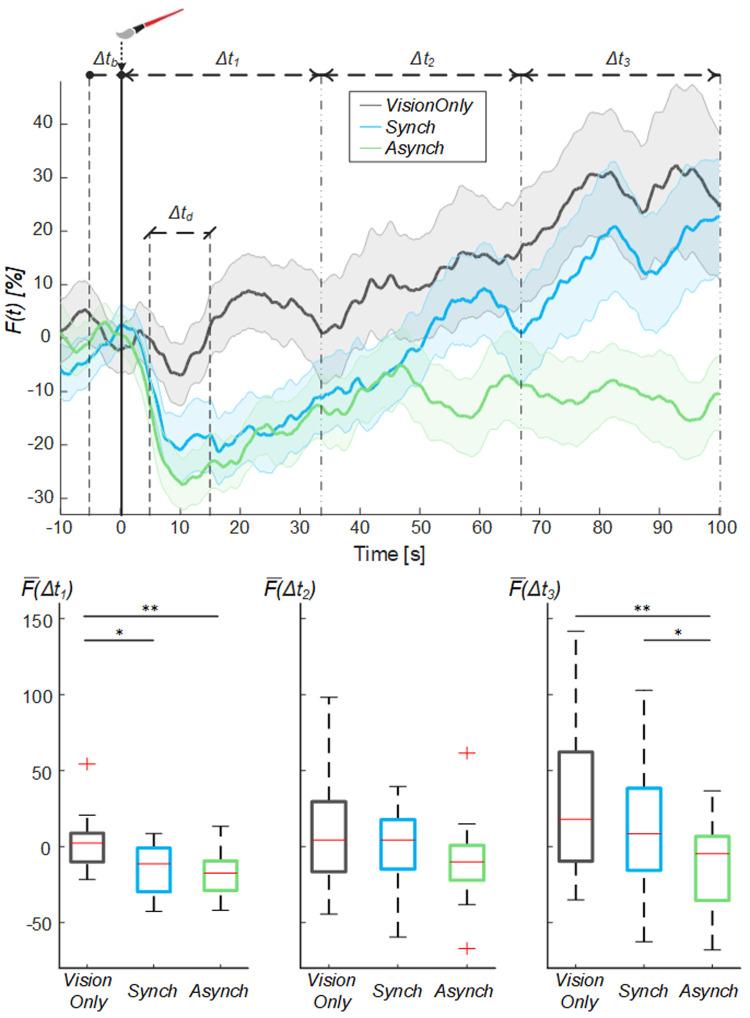
Mean blood flow data. Mean blood flow (F) averaged across participants for each condition, the shade represents the standard error (SEM), dashed lines indicate the time intervals where the mean baseline and drop values were calculated (Δt _b_ and Δt _d_) and the analysis performed (Δt _1_, Δt _2_ and Δt _3_). Time=0 sec is when the trial began. (Upper panel). Box and whisker plots relative to averaged blood flow values calculated in the selected intervals for Synch, *Asynch* and *VisionOnly* conditions (Lower panel): median (red lines), 1 ^st^ and 3 ^rd^ quartiles (box), lowest and highest values comprised within 1.5 times the interquartile range from the 1 ^st^ and 3 ^rd^ percentiles (whisker). * indicates a p-value <0.05.; ** indicates a p-value <0.01.

By analyzing the behavior of the mean blood flow ( *F(t)*) averaged among the participants, it is possible to note that right after the experiment began there was a drop in the mean blood flow, peaking at 10s. This drop was present in all conditions, but it was more evident for *Synch* and *Asynch* conditions ( Figure 4). After this drop, the *F(t)* tends to increase in all conditions. In particular, the mean blood flow behavior for *Synch* and *Asynch* conditions is similar until 50 s; after which, the value for the *Synch* condition has a higher increasing trend. The blood flow value for the *VisionOnly* condition is higher on average than for the other two conditions, probably because of the lower extent of the drop for this condition. For further analysis, the whole duration of each session (100s) has been divided into three time windows equal in length, in line with the neurogenic dynamic of the slow changes of the flow ^42^.

The rmANOVA run on the mean blood flow of the three temporal intervals of the trial showed the presence of both of the main factors time (F(2, 38) = 6.56, p = 0.006) and condition (F(2, 38) = 8.55, p = 0.004), and of their interaction (F(4, 76) = 2.89, p = 0.047). Post-hoc analysis showed that mean blood flow in the *VisionOnly* condition was significantly higher than in the *Asynch* one (d = 0.80, t(19) = 3.60, p = 0.005) and that mean blood flow value in the Δt _3 _temporal interval was higher than the other temporal intervals (Δt _1_: d = 0.70, t(19) = 3.15, p = 0.014; Δt _2_: d = 0.59, t(19) = 2.64, p = 0.041). Considering the interaction between the factors and given that our aim was to find a difference among conditions in a single time interval, we made three separate analysis, one for each time-interval: the *VisionOnly* flow in the first interval was significantly higher than both the others ( *Asynch*: d = 0.87, t(19) = 3.88, p = 0.003; *Synch*: d = 0.59, t(19) = 2.62, p = 0.042), whereas in the third interval, the *Asynch* flow was significantly lower than both the *Synch* and *VisionOnly* ones ( *Synch*: d = 0.60, t(19) = 2.67, p = 0.038; *VisionOnly*: d = 0.85, t(19) = 3.80, p = 0.003). No significant differences were identified in the second interval ( Figure 4).

**Figure 5.**
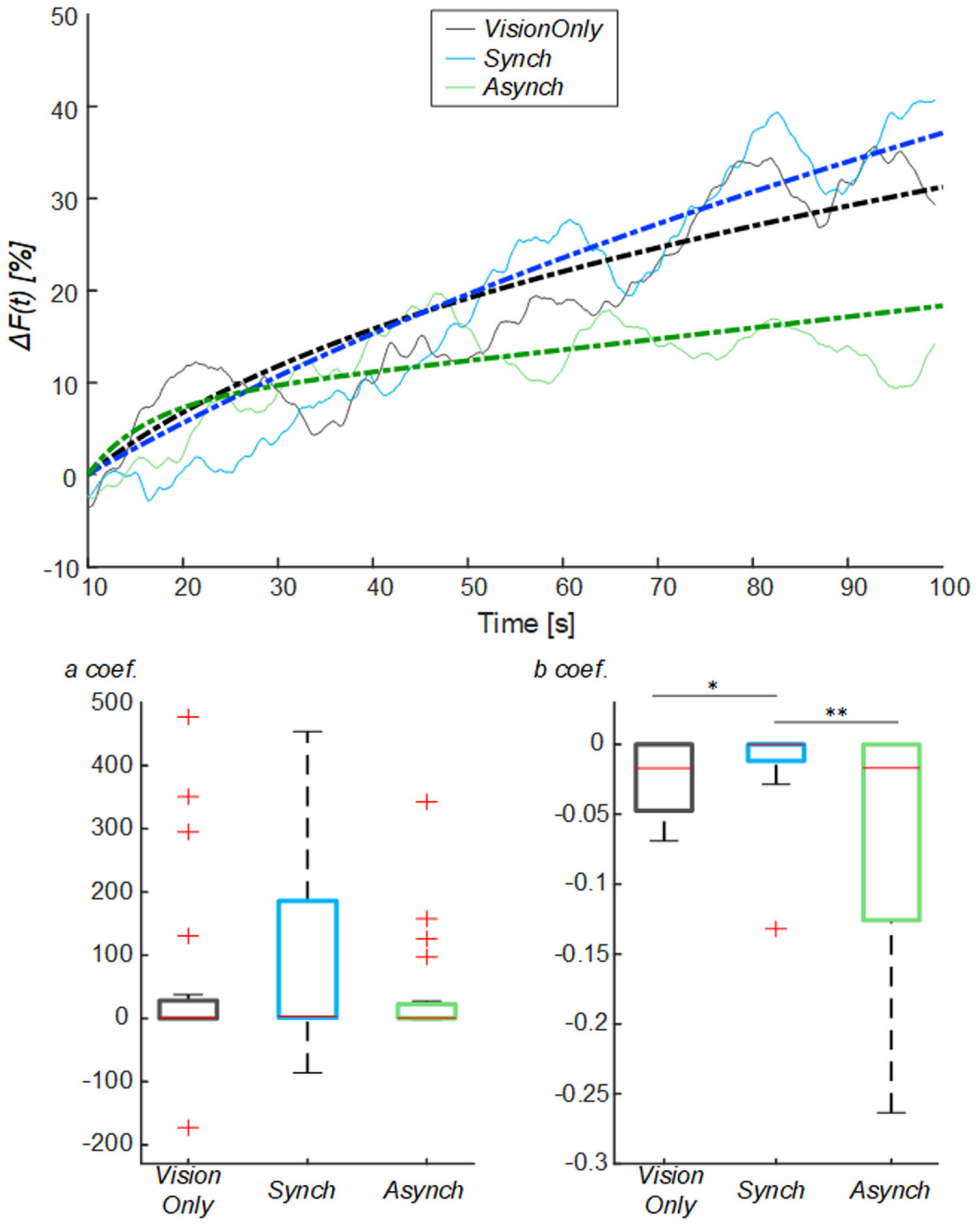
Exponential fitting of the mean blood flow data. Mean blood flow value from the drop ( *ΔF*) averaged across participants for each condition, dashed lines indicate the exponential curves that fit the data, averaged across participants (Upper panel). Box and whisker plots relative to *a* and *b* coefficients calculated in the selected intervals for *Synch* and *Asynch* and *VisionOnly* conditions (Lower panel): median (red lines), 1 ^st^ and 3 ^rd^ quartiles (box), lowest and highest values comprised within 1.5 times the interquartile range from the 1 ^st^ and 3 ^rd^ percentiles (whisker). * indicates a p-value <0.05.; ** indicates a p-value <0.01.

In this analysis, the flow value for each time window was not independent from the value in the previous window, so that higher *VisionOnly* value may have been the effect of its milder drop. To avoid such influence, an exponential curve was employed to fit the behavior of the blood flow from the drop identified at 10s ( Figure 5): the *a* fitting coefficient corresponds to the curve trend value; whereas the *b* coefficient corresponds to the rate to reach the trend value. The statistical analysis showed a difference in the curve fitting *b* coefficients ( *a* coefficient: χ ^2^(2, 38) = 3.70; p = 0.157; *b* coefficient: χ ^2^(2, 38) = 11.20; p = 0.004): the *b* values for the *Synch* condition were significantly higher than those of both the *VisionOnly* and *Asynch* conditions (r = 0.47, z = 2.10, p = 0.031; r = 0.49, z = 2.21, p = 0.005; respectively). This means a faster growth rate for the *Synch* condition ( Figure 5).

Both *a* and *b* coefficients were correlated to RHI index, vividness and prevalence scores (ρ >0.30, p <0.05) ( Table 2). In particular, the *b* coefficient related to the blood flow growth dynamics was more strongly correlated to questionnaire scores (ρ >0.40, p <0.05). There was no correlation with proprioceptive drift.

**Table 2.** Correlation analysis fitting coefficients and illusion outcomes.

	Coef. *a*		Coef. *b*	
	ρ	p	ρ	p
RHI index	0.33	0.037	0.43	0.007
Vividness	0.30	0.050	0.40	0.012
Prevalence	0.36	0.024	0.41	0.010
Prop. Drift	0.15	0.372	0.09	0.576

RHI, rubber hand illusion; Prop. Drift, proprioceptive drift.

**Figure 6.**
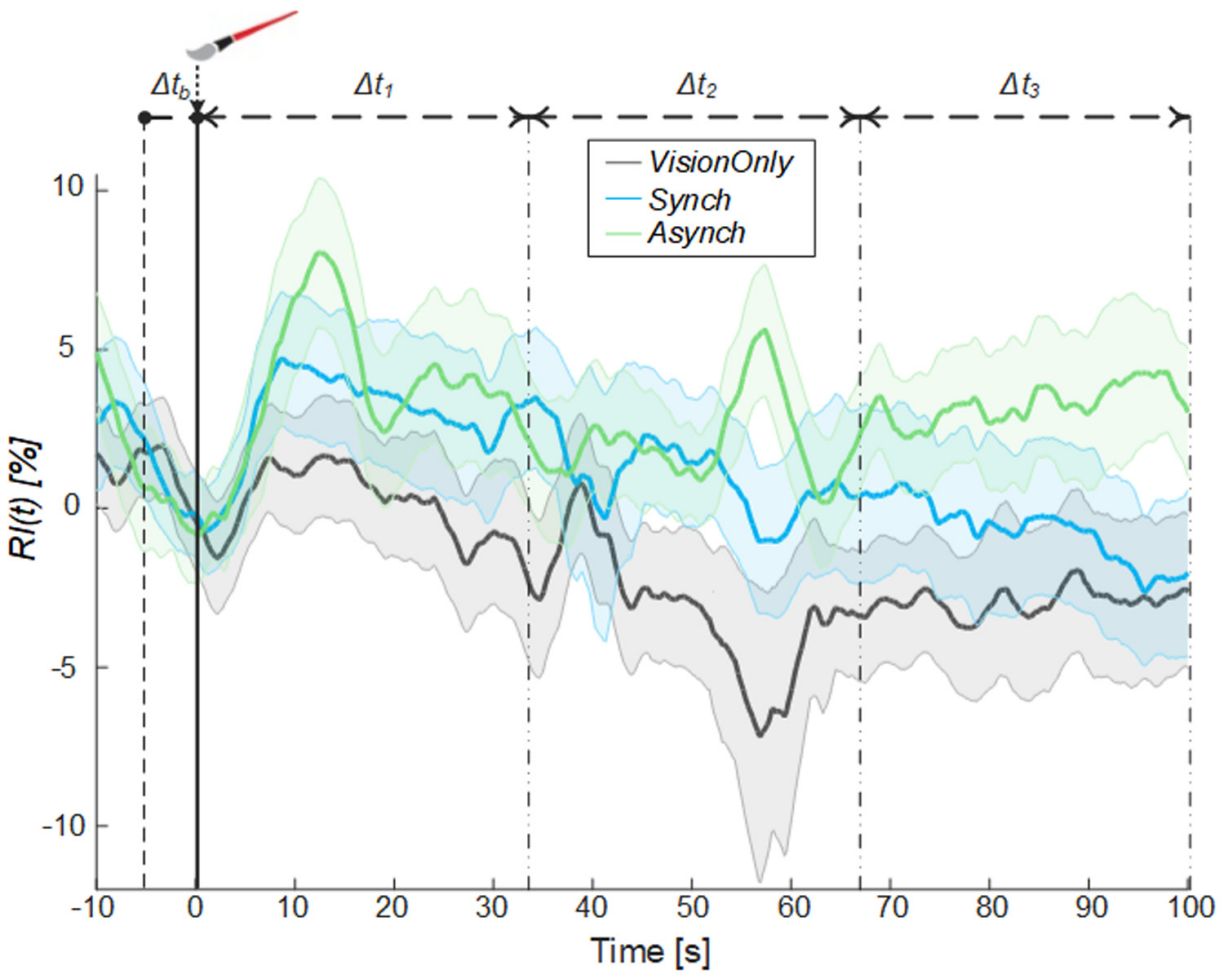
Resistance index. Resistance index ( *RI*) averaged across participants for each condition, the shade represents the standard error (SEM), dashed lines indicate the time intervals where the mean baseline value was calculated (Δt _b_) and the analysis performed (Δt _1_, Δt _2_ and Δt _3_). Time=0 sec is when the trial began.

Moreover, from the comparison of the systolic and diastolic variation of the flow, a resistance index reflecting the resistance caused by microvascular bed distal to the site of measurement ^38– 40^ was calculated ( Figure 6).

A significant negative correlation between the averaged blood flow and the resistance index values was highlighted in all time intervals (Δt _1_: ρ = -0.36, p =0.005; Δt _2_.: ρ = -0.38, p = 0.003; Δt _3_: ρ = -0.62, p <0.001), demonstrating that the measured increase in the blood flow was tightly related to a decrease in peripheral vessels resistance of the tested limb.

## Discussion

This study was designed to investigate possible changes in the blood flow directed towards the hand and forearm induced by the modulation of sense of limb ownership. To modulate limb ownership, we employed the RHI paradigm while the brachial artery blood flow of the homolateral limb was monitored.

Embodiment of a fake hand induced by the synchronous stimulation of the fake visible hand and real hidden hand of the participant ( *Synch* condition) was tested against the commonly-adopted control condition where embodiment was inhibited because the rubber hand and the participant’s own hand were asynchronously stroked ( *Asynch* condition).

Since we suspected that brush-stroking itself could have affected the flow independently from the achieved embodiment, a third condition was introduced as further control, where participants were instructed to simply look at the fake hand, without receiving any paintbrush stimulation on the real or on the fake hand ( *VisionOnly* condition). In the latter case, tactile stimuli were not present.

The first element to note is that the adopted experimental process induced a consistent modulation of the blood flow, characterized by having different behaviors for different conditions, but small variability across participants (SEM lower than 14, 11 and 16 % for *Synch*, *Asynch* and *VisionOnly* conditions, respectively). This suggest that the designed experiment was suited to assess the targeted phenomenon.

Furthermore, looking at the average blood flow dynamics, in all conditions, we found a common initial drop beginning at the start of the experiment, when the light was turned on in the fake hand compartment and the fake hand began to be stimulated. This drop was always followed by a progressive increase in the blood flow, which reached its maximal value at the end of the stimulation period ( Figure 4). Considering that the dynamic of the blood flow oscillation at frequencies of 0.02–0.05 Hz are mainly affected by sympathetic nerve activity ^42^, we chose to analyze the blood flow signal by splitting the recording session into three time intervals (33s each).

In the first time-interval (0<Δt _1_≤33.33s), a significant difference in mean blood flow was found between the conditions with brush stimulation ( *Synch* and *Asynch*) and the *VisionOnly* condition, while no significant difference was found between *Synch* and *Asynch* conditions.

The drop was a lot less evident in the *VisionOnly* condition, which was the only condition without any brush-stroking applied to the real hand. This suggests that the drop was due to the initial, mostly unexpected, tactile stimulation of the hidden hand caused by the brush, regardless of whether the stroke was synchronous or asynchronous and if an illusion was achieved. A blood flow drop due to tactile stimulus has already been reported ^43^.

While the flow in the *VisionOnly* condition had the milder drop and remained higher throughout the experiment, in the *Asynch* control condition the flow had a deep drop due to brush-stroking and it remained lower throughout the experiment. The only condition in which the blood flow dramatically increased after experiencing a deep drop was the synchronous brush-stroking condition, which was precisely the one designed to test the effect of the fake hand embodiment.

Indeed, focusing on the third time-interval (66.66<Δt _3_<100s), the blood flow of the asynchronous condition was significantly lower than the blood flow of all others. From the visual inspection of the evolution of the flow, different causes may be supposed to determine the difference between *Asynch* and *VisionOnly*, and the one between *Asynch* and *Synch*.

Compared to *Asynch*, the higher *VisionOnly* value in the third interval may be the effect of the previous milder *VisionOnly* drop or may be the effect of a slight embodiment induced by *VisionOnly.*

Indeed, despite embodiment illusion being strongly dependent on the integration of coherent multisensory afferences, previous studies hypothesized the mere vision of a fake hand placed in a congruent position as being able to induce some mild degree of embodiment ^44, 45^, while another study did not ^46^. In this work, the *VisionOnly* condition was introduced to control for the cause of the initial drop of the flow and collecting the embodiment measures during this condition was outside the original scope of the investigation. There are both technical and scientific reasons that may suggest not recording embodiment measures in *VisionOnly*: the most significant Botvinick and Cohen questions focus on being touched by the brush, and they lose meaning if the hand is not touched. Furthermore, the collection of the proprioceptive drift without relying on self-assessment measures could be misleading, considering that such measures are related to different aspects of the embodiment process ^36^. However, the lack of embodiment evaluation in the *VisionOnly* condition, especially of the proprioceptive drift, should be considered a limitation of the study and it is envisaged for future studies investigating the topic.

On the contrary, *Asynch* and *Synch* conditions did not experience different magnitudes of drop. Their significantly different value can only be due to their different effect on the body representation of the hand. This finding confirms our hypothesis: the embodiment illusion is able to significantly modulate the blood flow directed towards the tested forearm.

The main aim of the experiment was to investigate the modulation of the limb blood flow due to the embodiment of a fake hand and not the modulation due to tactile stimulation. To better highlight the effect of embodiment, we further corrected the blood flow changes for the initial drop by subtracting the value of the drop. Thus, we corrected the blood flow curves of all three conditions to have all of them start from the same value after the drop and modelled the following flow increase with an exponential curve. The comparison of the growth rate between conditions, employing the *b* fitting parameter, showed that the synchronous illusion condition had a significantly faster growth rate than the other conditions.

Moreover, the fitting coefficients positively correlate with most of the widely-validated measures of the illusion (i.e. RHI index, vividness and prevalence scores), confirming that the variation in the blood flow dynamics is linked to the change of embodiment level during the trials.

Generally, an increase in blood flow can be due to vasodilatation and /or an increase in cardiac output, both of which are mainly driven by the ANS.

To our knowledge, we are the first to demonstrate that embodying an artificial limb enhances the blood flow directed to the tested limb.

However, this is not the first finding involving an overactivation of the sympathetic nervous system in correlation with the illusion. Indeed, the skin conductance response, which is known to be mainly driven by the sympathetic branch of the ANS, is modulated by the illusion as well; a threat to the fake hand induces a stronger event-related skin conductance response when the hand is embodied ^30, 47– 49^. More recently, studies have demonstrated that the embodiment induced by the synchronous RHI brush-stroking, by itself without the need of any threat, enhances the spontaneous fluctuations of the skin conductance ^50, 51^.

Two tightly interconnected questions remain to be addressed: i) Is the hyperactivation of the ANS a local or a systemic response? And ii) Is the hyperactivation of the ANS just due to an alert after the perceived abnormalities linked with the experimental manipulation or, more intriguingly, is it due to a mismatch between the sensory, the motor and autonomic representations of the limb in the brain?

Limb vasoconstriction/dilatation is mainly affected by the ANS, specifically by the sympathetic and, to a lesser extent, by the parasympathetic activity ^52, 53^. Most systemic blood vessels, particularly those of the abdominal viscera and skin of the limbs, are constricted by the sympathetic stimulation. Contrarily, parasympathetic stimulation has almost no effect on most blood vessels, except for vasodilatation in certain restricted areas, such as in the blush area of the face ^52^.

Theoretically, the activation of the sympathetic branch of the ANS is a systemic response that recruits the whole body. However, in favor of the local response hypothesis, previous works found a selective cooling of the investigated hand compared to the contralateral one, when ownership over the rubber hand was induced ^54– 57^. For the sake of completeness, few other studies called into question the consistency of this phenomena ^58, 59^.

In the attempt to test the local specificity of our hypothesis, in a preliminary experiment run before the study, we tried to record the blood flow from both arms at the same time, but unfortunately, we realized that our experimental setup was not robust enough for that. Nonetheless, an indirect cue on the local specificity of the autonomic response can be gathered from the resistance index we extracted. Indeed, the resistance index value is determined by the arterial compliance (as opposite to the vessel’s stiffness) and vascular resistance, mainly due to the diameter of the vessels, that results in the normal loss of pulsatility as flow progresses from the arteries to the capillaries ^38– 40^.

If the ultrasonographic probe remains in the same spot, a decrease in the index is a sign of vasodilatation. The significant negative correlation between blood flow and resistance index percentage suggests that the change in the blood flow that we highlighted was tightly linked with the peripheral vessel resistance change. This, together with previous works not reporting significant heart rate variability differences between RHI illusion and control conditions ^50^, indirectly suggest the local specificity of the described phenomena.

Previously, it has been shown that the synchronous brush-stroking of the RHI procedure limited the increase in peripheral perfusion of the pierced skin of the hand induced by acupuncture ^60^. The reduction of a further evoked increase in skin perfusion coexists well with an increase in the general flow, them being competitive causes for a limited possible increase in the flow.

We reported an enhancement in limb blood flow with fake hand embodiment. Would this fit with reduced hand skin blood perfusion and with the previous reported cooling of the RHI tested hand, considering that blood perfusion is the main parameter affecting hand temperature ^61^? Skin perfusion may well not be representative of the whole flow directed towards the limb.

Indeed, the brachial artery blood flow we recorded is a cumulative measure of the flow through all the vessels placed distally with respect to the position of the probe (in our case the vessels of forearm and hand). The main part of this flow goes to the muscles (59% of the total flow), then to the bones and fat, which are relatively avascular under normal conditions (28%), and the remaining part to the skin (13%) ^62^. Blood flow recorded on the brachial artery is, hence, predominantly a measurement of the flow to the forearm and hand muscles and may not be correlated with what happens in the cutaneous bed where thermoregulation is performed.

In regard to the second question, whether the embodiment induced sympathetic hyperactivity is an unspecific alert response or the effect of the mismatched image of the body, there are conflicting hypotheses.

On one side, there is evidence towards the unspecific response: a state of anxiety has been reported to raise the blood flow in the forearm at rest ^25– 27^. Indeed, an increase in the sympathetic response can enhance the heart rate and decrease the resistance of peripheric vessels in the limb, increasing its blood flow. Therefore, sympathetic-induced skin vasoconstriction and muscle blood vessel dilatation may be explained as an unspecific alerting state to the defense "fight or flight" reaction: a preparatory adjustment for the muscular activation inseparable from these activities ^25– 27^.

On the other side, previous studies interpreted selective cooling of the tested hand and the increase in histamine reactivity after the RHI as an illusionary disownership and as a sign of rejection of the real hand in favor of the artificial limb ^54, 63^. A similar interpretation was provided for the downregulation of the somatosensory ^64, 65^ and motor ^66– 68^ cortices when the fake hand is embodied, resulting in a reduction of the amplitude of the recorded somatosensory and motor evoked potentials. In line with our finding, mounting an immune response towards a disowned limb would likely go through an increase in the blood flow towards the targeted limb. Also, this hypothesis fits with the presence of a correlation between the reduction of the skin conductance response to the threatening of a fake hand and the loss of its self-attribution ^69^. In regard to the time course of the measured effect, the difference between illusion and control conditions was demonstrated in the 66–100s time window after the beginning of the trial, whereas previous research demonstrated a sympathetic-induced increase in the variability of the non-specific skin conductance response in the 10–55s range ^50^. This temporal mismatch between the effect seen for the skin conductance response and the one seen for blood flow could be either due to the time that the flow needed to bounce back after the initial drop, or to the different sudomotor and vasomotor dynamics induced by the sympathetic activation. Indeed, a temporal dissociation between responses to sympathetic activity in the skin and muscle tissue was unveiled while monitoring sympathetic neural activity during handgrip. The former abruptly raised at the onset of the task and the latter increased slowly after a 60s latency ^70^. Despite having a cumulative faster growth rate, the synchronous illusory condition had slower initial (<30 s) dynamics. Interestingly, this behavior could be explained by the temporal dissociation of the ANS effect on skin and muscles. The more marked skin vasoconstriction elicited by a higher sympathetic activity in the *Synch* condition could slow down the rise of the blood flow in the first phase of the trial. However, in the following phase, when the increment of the vasodilation in skeletal muscles supersedes skin vasoconstriction, the blood flow level in the *Synch* condition rapidly grows and overcomes the others.

Previous work highlighted that it is possible to induce an increase in arousal just approaching a rubber hand placed in a congruent way with respect to the real hidden hand ^71^ and this effect could contribute to our outcomes. In order to assess the effect of the visual stroking per se, future studies could measure the blood flow when the stroking is delivered only on the rubber hand.

The RHI paradigm is an easy test to perform to evaluate embodiment. For its simplicity, low requirements and costs, it has been extremely widespread in research related to the representation of the body. It is not free of possible weaknesses ^72– 74^; one of them is that it lacks objective measures to evaluate its outcome. As previously suggested for the fluctuation in the non-specific skin conductance response ^50^, the blood flow may be a good candidate to evaluate the achieved embodiment as well. Indeed, the increase in the blood flow significantly correlated with all the other employed measures designed to rate the strength of the illusion (RHI index, vividness and prevalence scores), except for proprioceptive drift, which is often a dissociated measure weighting different aspects of the embodiment process ^36^.

In conclusion, we demonstrated that the modulation of the sense of limb ownership impacts on the blood flow directed to that limb. It is likely that the fake hand embodiment induced a sympathetic driven vasodilatation of the muscular territories downstream of the brachial artery.

This is a further proof that there is a bidirectional influence between the ANS and body ownership. Interoception, led by the afferent branch of the ANS, contributes to shape the sense of body ownership and, in turn, this modulation changes the autonomic outflow and becomes manifested through changes of the sudomotor ^50^ and vasomotor activity. Another interesting manifestation of such bidirectional influence is that embodiment of a fake hand seems to alter real hand temperature and, in turn, the propensity to perceive the embodiment illusion seems to be influenced by the hand temperature itself ^50^.

An important overlap of the brain circuits in charge of the representation of the body with those processing interoception and controlling body temperature, heart and vessel function has been recently confirmed by several experimental, meta-analytic and theoretical works ^75– 78^, which highlighted the main role played by premotor, parietal-temporal, cingulate cortex, the amygdala and the insula.

This is the first study demonstrating that the update of the perceptual status leading to a change of a limb presence in the body representation is paralleled by an enhancement in the perfusion of the tested limb. It also opens the intriguing question of whether the reported changes are unspecific effects of an alert response regarding the whole body or, on the contrary, are specifically causally and topographically related to the limb, the representation of which was modulated. We speculated on this topic providing cues in favor of the latter. This, however, remains an extremely interesting question, a matter still open for future research.

## Data availability

### Underlying data

Mendeley Data: Embodying an artificial hand increases blood flow to the investigated limb. http://dx.doi.org/10.17632/pcbtb8xfg6.1 ^41^.

This project contains the following underlying data:

- Dataset.mat (matrices of data, Matlab dataset)
- Table_MeanBF.csv (mean blood flow [20 participants X 12000 samples (from -20s to 100s at 100Hz) X 3 conditions (order: VisionOnly, Synch, Asynch)])
- Table_PD.csv (proprioceptive drift [20 participants X 2 conditions (order: Synch, Asynch)])
- Table_RHIi.csv (RHI index [20 participants X 2 conditions (order: Synch, Asynch)])
- Table_RI.csv (resistance index [20 participants X 12000 samples (from -20s to 100s at 100Hz) X 3 conditions (order: VisionOnly, Synch, Asynch)])
- Table_T.csv (prevalence score [20 participants X 2 conditions (order: Synch, Asynch)])
- Table_V.csv (vividness score [20 participants X 2 conditions (order: Synch, Asynch)])

Data are available under the terms of the Creative Commons Attribution 4.0 International license (CC-BY 4.0).
