# Embodying an artificial hand increases blood flow to the investigated limb

**DOI:** 10.12688/openreseurope.13641.3

**Published:** 2022-04-21

**Authors:** Giovanni Di Pino, Alessandro Mioli, Claudia Altamura, Marco D'Alonzo

**Affiliations:** 1 NeXT: Neurophysiology and Neuroengineering of Human-Technology Interaction Research Unit, Campus Bio-Medico University of Rome, via Alvaro del Portillo, 5, Rome, 00128, Italy; 2 Headache and Neurosonology Unit, Neurology, Campus Bio-Medico University of Rome, via Alvaro del Portillo, 200, Rome, 00128, Italy

**Keywords:** Autonomic nervous system, upper limb embodiment, blood flow, Rubber Hand Illusion paradigm, peripherical vascular resistance, brachial artery, body representation

## Abstract

**Background: **The autonomic nervous system is the main determinant of the blood flow directed towards a body part, and it is tightly connected to the representation of the body in the brain; would the experimental modulation of the sense of limb ownership affect its blood perfusion?

**Methods: **In healthy participants, we employed the rubber hand illusion paradigm to modulate limb ownership while we monitored the brachial artery blood flow and resistance index within the investigated limb.

**Results: **In all conditions with brush-stroking, we found an initial drop in the blood flow due to tactile stimulation. Subsequently, in the illusion condition (where both the rubber and real hand synchronous brush-stroking were present), the blood flow rose significantly faster and reached significantly higher values. Moreover, the increase in blood flow correlated with the extent of embodiment as measured by questionnaires and correlated negatively with the change of peripherical vascular resistance.

**Conclusions: **These findings suggest that modulating the representation of a body part impacts its blood perfusion.

## Plain language summary

The autonomic nervous system controls the visceral body and its blood perfusion, by adapting it to our behavior. Its activity is influenced by cognitive and emotional processes, and is bidirectionally connected to the network hosting the body representation, while contributing to the creation of this ultimate representation. By exploring the brachial artery blood flow recording during the rubber hand illusion paradigm, we observed that modulating the belonging of a body part to the body representation seems to increase its perfusion, through a sympathetic-driven downstream vasodilatation. The blood flow increase was correlated with the achieved level of fake hand embodiment. This raises intriguing questions as to the specificity of the blood flow enhancement, and on the essence of its causal connection with the alteration of the sense of embodiment of the limbs.

## Introduction

The autonomic nervous system (ANS) takes care of the involuntary control of the visceral body. Glands, smooth and cardiac muscles are regulated to maintain the body homeostasis and to adapt the digestion, body temperature, ventilation, cardiac activity and regional blood flow to our behavior.

Despite the ANS being mainly a low-level control system, it is strongly influenced by emotive and cognitive processes. Depending on emotions and feelings, connections between the amygdala and the medial cortices (anterior cingulate, insular, and ventromedial prefrontal cortex) in association with the dorsal pons and hypothalamus, modulate blood pressure, pupil size, heart rate and electrodermal activity ^1^. Moreover, ANS homeostatic information related to pain, temperature, pH, carbon dioxide, and oxygen are sent to the insula and interact with somatosensory processing. This has been suggested to have a role in the construction of the body representation ^2^.

Pathways and cortical centers of interoceptive and exteroceptive information often overlap. For example, somatosensori-motor cortices, extra-striate body area and the dorsal precuneus control gastric activity, digestion, cardiac output and heart rate, and they are also involved in mapping bodily space through touch, action and vision. In particular, the primary sensorimotor cortex receives both tactile and visceral afferents combining internal and external bodily information ^3– 6^.

Evidence of the tight connection between the ANS and central body representation may be derived from complex regional pain syndrome (CRPS) ^7^, where the alteration of the brain representation of a body part impacts on its autonomic neural pathway. In CRPS, autonomic dysfunction results in changes to the skin blood flow, leading to warmer limbs, change of colour, edema, longer nails and abnormal sudomotor activity ^8^. CRPS is usually triggered by a limb-related trauma and a subsequent period of immobilization. The associated pain is also related to sympathetic hyperactivity, so patients benefit from early sympathetic blockade ^9^. The strange association of a “neglect-like” syndrome ^10^ with an over-representation of the affected hemispace ^11^, and of an enlargement of the affected limb motor cortex ^12^ with a reduction in its primary sensory cortex ^13^ could imply that a disordered body representation affects CRPS pathogenesis. Moreover, both pain and autonomic symptoms are relieved with interventions manipulating the representation of the limb, such as mirror therapy ^14^; minimizing lens ^15^; or prism adaptation ^7, 11^.

Aside from pathological models, in healthy subjects the meaning and strength of the relationship between body representation and interoceptive signals is still matter of debate. For example, emerging evidence suggests that interoceptive information such as cardiac feedback modulates the visual body perception ^16^ and influences one’s own body awareness ^17, 18^ or, vice-versa: changes in body-ownership and self-identification alter the ability to detect internal body signals ^19^. Furthermore, interoceptive sensitivity seems to predicts the malleability of participants’ body representation ^20^.

The ANS regulates blood perfusion; for instance, to the viscera-to-muscle redirection of blood flow during the fight or flight response, or the reduction of wound hemorrhages, thermoregulation and thermomimesis ^21^. For these responses, the nucleus of the solitary tract integrates signals from the periphery and from higher brain centers, to control vagal and sympathetic outflow ^22^. Preoptic hypothalamic and forebrain centers interact with the periaqueductal gray and raphe nuclei ^23^ when blood flow to the limb is modulated by cognitive and emotional processes, as well as attention ^24^, and anxiety ^25– 27^. The amygdala, involved in vigilance and arousal, and the habenula, activated by aversive events or missing rewards, control vasoconstriction triggered by salient alerting stimuli ^28^.

Hitherto, we know that i) the central ANS is tightly connected with circuits underlying the representation of the body, ii) cognitive processes influence central ANS control of the local blood flow, and iii) a syndrome due to an alteration of the limb representation (i.e. CPRS) presents an autonomic-driven dysfunction of the vascular supply to the affected limb.

Altogether, this raises the question that modifying the brain representation of a body part could result in a change of its blood perfusion; however, this has never been demonstrated.

A simple way to modulate the body representation is using the rubber hand illusion (RHI), a perceptual illusion caused by the synchronous brush-stroking of the hidden participant’s real but hidden hand and a visible but fake hand ^29^. Spatio-temporal congruency of visuo-tactile stimuli is mandatory for the illusion to arise, owing to the dependence upon Bayesian integration of different information into a pre-existent internal body map to create a sense of body ownership ^30– 32^. Indeed, the illusion is abolished when the visual and somatosensory stimulation are presented asynchronously.

A link between autonomic mechanisms and cognitive processes underlying the body representation was previously demonstrated using RHI paradigm, including altered temperature regulation while inducing body ownership over the fake hand ^20, 33^. The occurrence of the RHI results in disownership and a decrease in skin temperature of the real hand ^34^, but the consistency of such findings is still under debate ^35, 36^. Furthermore, increased fluctuations in the skin conductance correlate with the onset and the strength of the illusion during the RHI ^37^. On the other hand, the relationship between RHI-generated ownership and these interoceptive measures was not always reported ^38^ while other interoceptive indexes were not found to be correlated with illusion strength: e.g. the scores in heartbeat counting tasks ^39, 40^. Interestingly, artificially-induced peripheral ischemia modulated the proprioceptive drift during the RHI paradigm ^41^.

This work assessed whether modulating the belonging of the upper limb to the body representation would impact on its perfusion. In healthy subjects, we recorded the brachial artery flow of the limb involved in three different RHI conditions: synchronous *(Synch)*, asynchronous brush-stroking ( *Asynch*), and the mere sight of the fake hand while the hidden real hand was not stimulated ( *VisionOnly*).

## Methods

### Participants

Twenty participants were recruited among the friends and relatives of collaborators of Neurophysiology and Neuroengineering of Human-Technology Interaction (NeXT) Research Unit that volunteers to participate to the study. Inclusion criteria were to be older than 18 years, to be naïve to the RHI protocol, to have normal hand sensation and normal, or corrected to normal, vision. To our best knowledge, we are the first to systematically measure blood flow on the forearm and on the hand while participants experience the rubber hand illusion and, for reason, it was not possible to calculate participants sample size with *a priori* power analysis. Therefore, in this case, the number of participants was chosen equal to previous RHI studies ^30, 32, 42– 45^. Participants were enrolled after signing written informed consent to the participation and publication of the data, including the permission for the treatment of their images. The experimental procedures were approved by the Ethics Committee of the Università Campus Bio-Medico di Roma (EMBODY protocol) and carried out according to the Declaration of Helsinki and its future amendments.

### Experimental procedure

**Figure 1.**
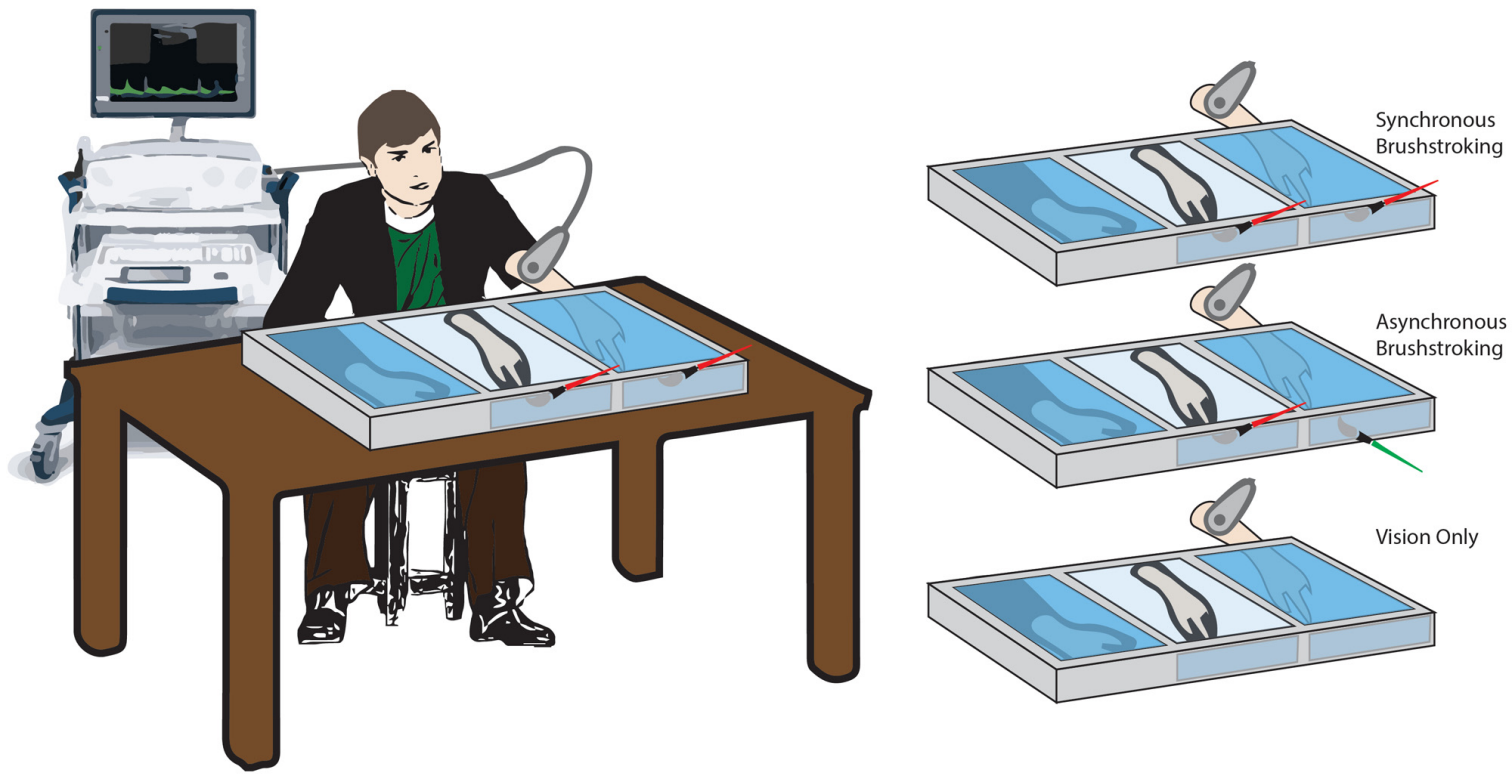
Schematic illustration of the experimental conditions. Setup and rubber hand illusion paradigm conditions.

The study was performed in a dedicated room of the NeXT Research Unit from September 2018 to June 2019. Participants were seated in front of a custom-made experimental set-up, made of three parallel compartments (L × W × H = 40 × 60 × 20 cm each) covered by a two-way mirror ( Figure 1) ^42, 46^. They could see the content of each compartment only when the experimenter turned the relative internal light on. Then, participants were invited to place their forearms inside the two lateral compartments while their shoulders were covered by a black cloak. A left rubber hand, matching the participant’s gender, was placed in the central compartment of the structure, 15cm apart from the real hidden left hand of the subject. The left hand was tested because it is thought to be easier to induce the RHI ^47^.

Three conditions were tested for each participant, administered in a random order:

- *Synchronous* ( *Synch*) *condition*: a well-trained experimenter used two identical paintbrushes to stroke both the second digit of the rubber hand and the corresponding digit on the real hidden hand. The tactile stimulation was delivered at a frequency of 1Hz. The brushstroke duration was about 0.6–0.7s, and it was delivered from the proximal to the distal phalanx.
- *Asynchronous* ( *Asynch*) *condition*: similarly, the experimenter used the paintbrushes to stroke the second digit of the participant, with a small temporal delay (about 0.5s) between the stimulus delivered to the rubber hand and that delivered to the real hand ( Figure 1).
- *Vision only condition*: in this case, no stroking was delivered to either the rubber or the real hidden hand. The participant was instructed to simply look at the rubber hand for the entire duration of the condition. Such condition was performed in order to control for the effect of mere tactile stimulation on the blood flow and was considered an additional condition of no embodiment ^43^. As in previous studies ^43^, questionnaire outcomes were not recorded in this condition.

Each condition lasted 100s. The order of each condition was evenly distributed across participants in order to decrease the order effect in our findings (first position in the sequence of performed conditions: 7- *VisionOnly*, 6- *Synch* and 7- *Asynch*; second position: 6- *VisionOnly*, 7- *Synch* and 7- *Asynch*; third position: 7- *VisionOnly*, 7- *Synch* and 6- *Asynch*).

Blood flow was collected at a sampling rate of 100 Hz by using a Multidop-X DWL (Elektronische Systeme GmbH, Germany). The device probe was placed over the brachial artery on the medial side of the tested (i.e. left) arm. We employed a 4 MHz DWL ultrasound probe, which is well-suited to monitoring blood flow of the brachial artery since it can penetrate approximately 12–30 mm. The probe was kept still by the experimenter during the whole protocol. The brachial artery was selected because it is the major blood vessel located in the upper arm: the main supplier of blood to the arm and hand.

Every 1.3s (i.e. using 130 samples each time), from the blood flow data the device calculated and saved three parameters: the mean blood flow, the peak of systolic flow and the peak of the diastolic blood flow. Once the blood flow was stable, it was recorded for 120s, from 20s before the compartment’s lighting was turned until 100s after it. For each condition, about 92 measures (i.e. 120s/ 1.3s = 92 times) of each parameter were recorded (about 77 if considering the period when compartment’s lighting was on).

### Embodiment measures

The measures of embodiment were collected to assess the embodiment of the rubber hand induced across different conditions.

The proprioceptive drift was assessed ^29^ by asking the participants to verbally report a number on a measuring tape (reflected on the two-way mirror) that corresponded to the perceived location of their left index finger while maintaining their hands still and relaxed.

For each condition, the perceived location was collected twice, before and after the administration of the condition. To guarantee a random offset, the measuring tape was moved before every assessment. Positive differences between the hand position estimated pre- and post-stimulation indicate a drift of the perceived location of the participants’ hand towards the rubber hand.

Then, the experimenter handed the participant a nine-item questionnaire with three questions aimed at investigating the extent of the self-attribution of the rubber hand and six control questions testing participant susceptibility ^29^ ( Table 1). The participants were asked to rate the extent to which these items did or did not apply to them, using a seven-point scale. On this scale, -3 meant ‘‘absolutely certain that it did not apply,’’ 0 meant ‘‘uncertain whether it applied or not,’’ and +3 meant ‘‘absolutely certain that it applied’’. This questionnaire was provided with two additional items to rate the vividness (0 – 10) of the perceived illusion (i.e. how realistic the illusion was when it was experienced) and the prevalence (0 – 100%), which reflected the percentage of time that the illusion was experienced (i.e. how long with respect to the length of section was the perception of the illusion).

**Table 1.** List of questionnaire items.

Questionnaire	Item	Rating
Item 1	It seemed as if I were feeling the tactile stimulation at the location where I saw the visible hand touched	-3 – +3
Item 2	It seemed as though the stimulation I felt was caused by the touch on the visible hand	
Item 3	I felt as if the visible hand was mine	
Item 4	I felt as if the position of my real hand was drifting towards the visible hand	
Item 5	It seemed as if I had more than two hand or arm	
Item 6	It seemed as if the tactile stimulation I was feeling came from somewhere between my own hand and the visible one	
Item 7	I felt as if my real hand were turning ‘rubbery’	
Item 8	It appeared as if the position of the visible hand was drifting towards my real hand	
Item 9	The visible hand began to resemble my own hand, in terms of shape, skin tone, freckles or some other visual features	
Vividness	How realistic and life-like was the illusion that the visible hand was yours when it was experienced?	0 – 10
Prevalence	How long with respect to the length of section was the perception of such illusion?	0 – 100%

The overall experimental session lasted about 30 minutes for each participant. This included reading and signing informed consent, participant setup, probe positioning on the artery, administration of three experimental conditions (one time for each condition) and, for each condition, performing the proprioceptive measures and filling out the questionnaire.

### Data analysis

The Kolgomorov-Smirnov test (p >0.05) was used to verify that the data relative to the typical RHI outcomes (nine-item questionnaire, vividness, prevalence score and proprioceptive drift) were normally distributed. To verify that the responses to the questionnaires were not due to the participants’ suggestibility, the mean score of the three items employed to measure the effective illusion was compared against the mean score of six items that served as controls for compliance, suggestibility, and ‘‘placebo effect’’, by using a two-tailed paired t-test.

The RHI index, which expresses the difference between the mean scores of the illusion items and the control items ^48^, was calculated. This was performed for each condition and considered as the “illusion outcome” in the following analyses.

Questionnaire outcomes were analyzed with paired t-tests to highlight differences between the illusion condition ( *Synch*) and the asynchronous control condition ( *Asynch*). After checking the sphericity of the distribution of the values by using a Mauchly test, a repeated measures ANOVA (rmANOVA) with one factor ( *condition*) was performed with Greenhouse-Geisser adjustment on the proprioceptive data. Hence, a Tukey’s honest significant difference test was employed as post-hoc analysis. Effect size (d) for each comparison was also calculated as Cohen’s d.

Regarding the blood flow signal analysis, the mean blood flow ( *f*) was smoothed by using a moving average 5s window to eliminate the high frequency noise. The use of this moving-average window eliminates high-frequency noise without attenuating the frequency of interest ^49^ (i.e. 0.02–0.05 Hz ^50^). In order to minimize the influence of inter-individual variability and of the circumstance on which the experiment was run (e.g. the room temperature), the extracted measure was expressed as percentage change with respect to a baseline value for each trial ( *F(t)*), for simplicity hereafter called mean blood flow, calculated using the following equation:

$$F\left(t\right)=100*\frac{\left(f\left(t\right)-\bar{f}\left(\Delta t_{b}\right)\right)}{\bar{f}\left(\Delta t_{b}\right)}\;(1)$$

Where *f(t)* is the blood flow value at certain time t, $\bar{f}$ (Δt _b_) is the value of baseline calculated as blood flow values averaged on the last 5s window of the baseline interval (i.e. Δt _b_ = [-5s, 0s]). This baseline duration was selected based on visual inspection, where 5s duration was the best compromise between the stability of the signal before starting the experiment and after starting acquisition. Furthermore, additional analysis evaluating the effect of longer baseline normalization on analysis outcomes highlighted no significant change in obtained results (Supplementary extended data ^51^). These values were calculated for each condition and participant.

After that, the *F*(t) values were averaged across participants for each condition.

In order to identify the notable time intervals where further statistics could be performed, we computed a point-by-point ANOVA using multiple datasets and a permutation testing to find the cluster of significant differences among the three conditions. In particular, we employed 1000 permutations in *clustersize-based permutation testing and percentile of mean cluster sum* as method to define the threshold distinguishing between “significant” and “non-significant” clusters ^52^. This method returned two significantly different clusters: one in the interval between 5 and 31s (Δt _1_) and the other between 69 and 100s (Δt _2_). Then, further analysis was focused on these two intervals where the average value of the blood flow was extracted in the different time-intervals for each condition and participant ( $\bar{F}$ (Δt _1_) and $\bar{F}$ (Δt _2_)). After checking the normality of the data by using the Kolmogorov-Smirnov test (p >0.05), a Mauchly test was employed to verify the sphericity of the distribution of the values and a repeated measures ANOVA (rmANOVA) with two factors ( *condition* and *time*) was performed with Greenhouse-Geisser adjustment. Subsequently, a Tukey’s honest significant difference test was employed as post-hoc analysis. Lastly, the effect size (Cohen’s d) was calculated for each comparison.

A drop of the mean blood flow values was identified at around 10s from the beginning of each conditions. $\bar{F}$ *(Δt _d_)* (i.e. the drop in signal) was calculated as blood flow value averaged on a 10s-window centered 10s after the beginning of each trial (i.e. Δt _d_ = [5s, 15s]). For each condition, the drop in signal value was analyzed to assess whether it was significantly lower than baseline (i.e. 0 value), by using a one-sample t-test. Additionally, a repeated measure ANOVA was employed to detect differences between conditions. Considering the obtained significant difference, we corrected the blood curves (relative shift along the y-axis) of all the three conditions to make all of them starting from the same flow value after the drop and analyze the blood flow increase independently to the drop. This was done by subtracting the value of the drop to the mean blood flow as in the following equation:

$$\text{Δ}F(t)=F(t)-\bar{F}(\text{Δ}t_{d})\;(2)$$

The corrected signals in the interval between 10s and 100s were fitted by using an exponential curve:

$$y(t)=a*(1-e^{\left(b*(t-10s)\right)})\;(3)$$

with *a* and *b* as the coefficients of the curve employed to fit the data, where *a* is the value to which the curve asymptotically tends (i.e. trend value): the higher the *a*, the higher the trend value. Furthermore, *b* is the rate to reach the trend value: the higher the absolute value of *b*, the faster the curve rate. Considering that the coefficient data were not normally distributed, *a* and *b* coefficients in the different conditions were compared using a Friedman test, and post hoc tests with Bonferroni correction were employed for pairwise comparisons. Effect size (r) was calculated as z/√n, where z is the test statistic for the signed-rank test and n is the number of observations.

The link between blood flow changes and embodiment was investigated by correlating (Spearman’s) *a* and *b* coefficients with the illusion outcomes in *Synch* and *Asynch* condition pooled together.

The resistance index ( *ri*) ^53– 55^ was calculated as:

$$ri(t)=\frac{f_{Syst}(t)-f_{Dias}(t)}{f_{Syst}(t)}\;(4)$$

where *f _Syst_* is the systolic peak blood flow, and *f _Dias_* is the diastolic blood flow. The obtained signal of the resistance index was smoothed and normalized by using the same strategy of equation (1), the result of which is labeled the resistance index ( *RI(t)*). The average value of the resistance index was extracted in Δt _1_ and Δt _2_ time-intervals for each condition and participant ( $\bar{RI}$ *(Δt _1_)* and $\bar{RI}$ *(Δt _2_)*). Correlations (Pearson’s coefficients) between the resistance index and mean blood flow values in all conditions were calculated for both time intervals (Δt _1_ and Δt _2_).

No trial or data was rejected or excluded from the analysis.

The analysis was performed with Matlab2015a (Mathworks), a freely available alternative software is GNU Octave and JASP for statistical analysis.

## Results

Twenty volunteers took part in the experiment (age: 29.55 ± 6.12; 12 M, 8 F; 20 right-handed as by self-report).

**Figure 2.**
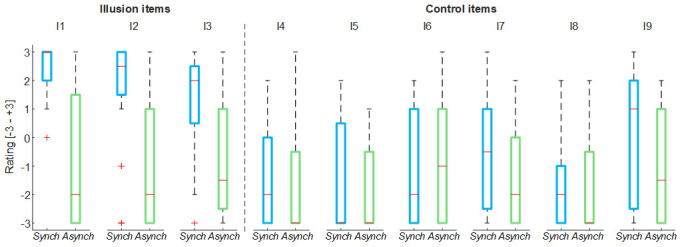
Nine-item questionnaire outcomes. Box and whisker plots of nine-item questionnaire outcomes for *Synch* and *Asynch* conditions: median (red lines), 1 ^st^ and 3 ^rd^ quartiles (box), lowest and highest values comprised within 1.5 times the interquartile range from the 1 ^st^ and 3 ^rd^ percentiles (whisker).

For both stroking ( *Synch* and *Asynch*) conditions, the mean value of the illusion items of the self-evaluation questionnaire was higher than the mean value of the control items ( *Synch*: d = 1.82; t(19) = 7.95, p <0.001; *Asynch*: d = 0.50; t(19) = 2.19, p = 0.041), thus the group of participants found to be generally not suggestible ( Figure 2) ^29^.

**Figure 3.**
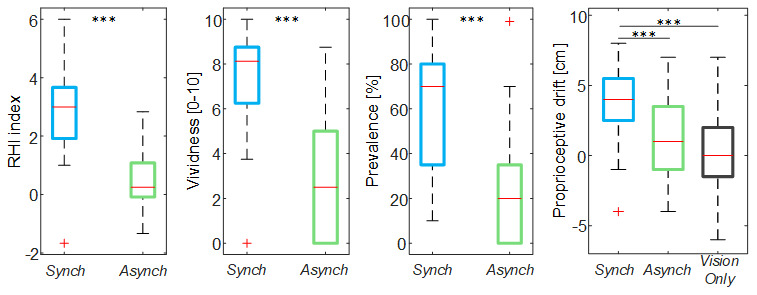
Illusion outcomes. Box and whisker plots of the illusion outcomes (rubber hand illusion [RHI] index, vividness, prevalence rating and proprioceptive drift) for *Synch*, *Asynch* and *VisionOnly* conditions: median (red lines), 1 ^st ^and 3 ^rd^ quartiles (box), lowest and highest values comprised within 1.5 times the interquartile range from the 1 ^st^ and 3 ^rd^ percentiles (whisker). *** indicates a p-value <0.001.

The illusion items scores were significantly higher in the *Synch* condition than the *Asynch* condition for all of the embodiment measures extracted from the questionnaire (RHI index: d = 1.17; t(19) = 5.12, p <0.001; vividness: d = 1.34; t(19) = 5.84, p <0.001; prevalence: d = 1.22; t(19) = 5.31, p <0.001; proprioceptive drift: d = 0.84, t(19) = 3.68, p = 0.002) ( Figure 3). The rmANOVA conducted on the proprioceptive drift showed a significant effect of the condition (F(2, 38) = 7.73, p = 0.004).

The proprioceptive drift relative to the *Synch* condition was significantly higher than both the others ( *Asynch*: d = 0.82, t(19) = 3.68, p = 0.004; *VisioOnly*: d = 0.84, t(19) = 3.77, p = 0.004), while *Asynch* and *VisionOnly* were not significant different (d = 0.24, t(19) = 1.06, p = 0.546).

In general, these findings confirm that participants effectively experienced the RHI in the *Synch* condition.

**Figure 4.**
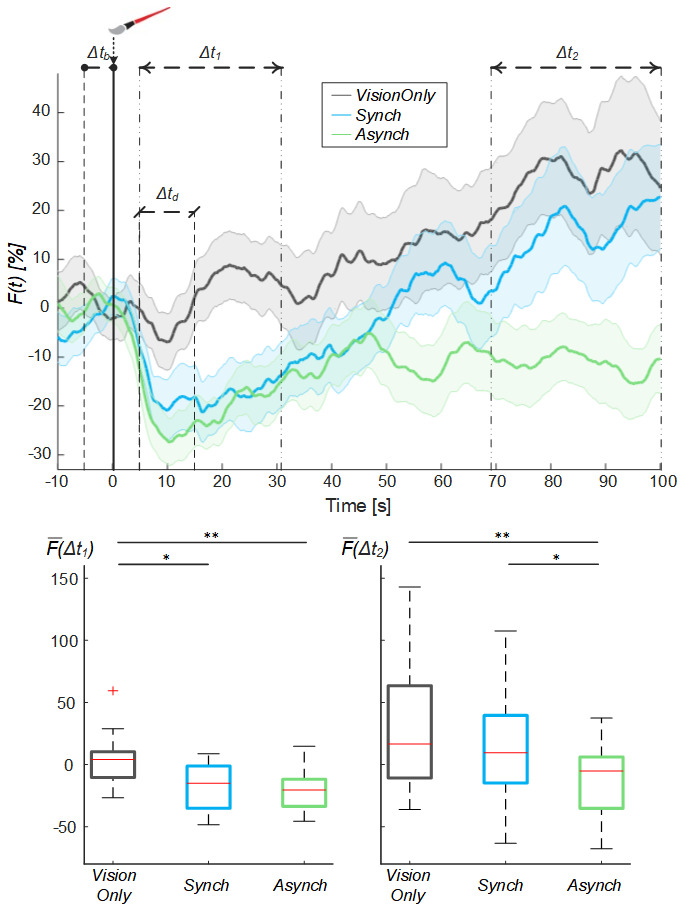
Mean blood flow data. Mean blood flow (F) averaged across participants for each condition, the shaded area represents the standard error (SEM), dashed lines indicate the time intervals where the mean baseline and drop values were calculated (Δt _b_ and Δt _d_) and the analysis performed (Δt _1_ and Δt _2_). Time=0 s is when the trial began. (Upper panel). Box and whisker plots relative to averaged blood flow values calculated in the selected intervals for *Synch*, *Asynch* and *VisionOnly* conditions (Lower panel): median (red lines), 1 ^st^ and 3 ^rd^ quartiles (box), lowest and highest values comprised within 1.5 times the interquartile range from the 1 ^st^ and 3 ^rd^ percentiles (whisker). * indicates a p-value <0.05.; ** indicates a p-value <0.01.

By analyzing the behavior of the mean blood flow ( *F(t)*) averaged across the participants, it is noticeable that right after the experiment began there was a drop in the mean blood flow, peaking at 10s. This drop was shallower in *VisionOnly* ( Figure 4). After this drop, the *F(t)* tends to increase in all conditions. In particular, the mean blood flow behavior for *Synch* condition starts to have a higher growth than *Asynch* already after 25s, and higher than *VisionOnly* after about 48s.

The rmANOVA conducted on the mean blood flow of the two “significant” cluster intervals of the trial showed the presence of both of the main factors time (F(1, 19) = 12.00, p = 0.003) and condition (F(2, 38) = 9.29, p = 0.001), and of their interaction (F(2, 38) = 3.29, p = 0.049). Considering the interaction between the factors and given that our aim was to find a difference across conditions within a single time interval, we conducted two separate post-hoc analyses using a Tukey’s honest significant difference test, one for each time-interval: the *VisionOnly* flow in the first interval was significantly higher than both the others ( *Asynch*: d = 0.87, t(19) = 3.88, p = 0.003; *Synch*: d = 0.59, t(19) = 2.62, p = 0.042), whereas in the second interval, the *Asynch* flow was significantly lower than both the *Synch* and *VisionOnly* flow ( *Synch*: d = 0.60, t(19) = 2.67, p = 0.038; *VisionOnly*: d = 0.85, t(19) = 3.80, p = 0.003) ( Figure 4).

In this analysis, the flow value for each time window was not independent from the value in the previous window, so that higher *VisionOnly* value may have been the effect of its milder drop. In particular, focusing on the drop values calculated as blood flow value averaged on Δt _d_ interval, we found that only drop values in *Synch* and *Asynch* conditions were statistically lower than the baseline ( *Synch*: t(19) = -4.52, p < 0.001; *Asynch*: t(19) = -6.78, p <0.001). The values of the drop were (mean ± st. dev.): -18.4 ± 18.3% and -24.8 ± 16.4% for *Synch* and *Asynch* condition respectively, while just -3.4 ± 16.9%, for *VisionOnly*. A significant difference among conditions was also observed (F(2, 38) = 8.48, *p* = 0.002).

**Figure 5.**
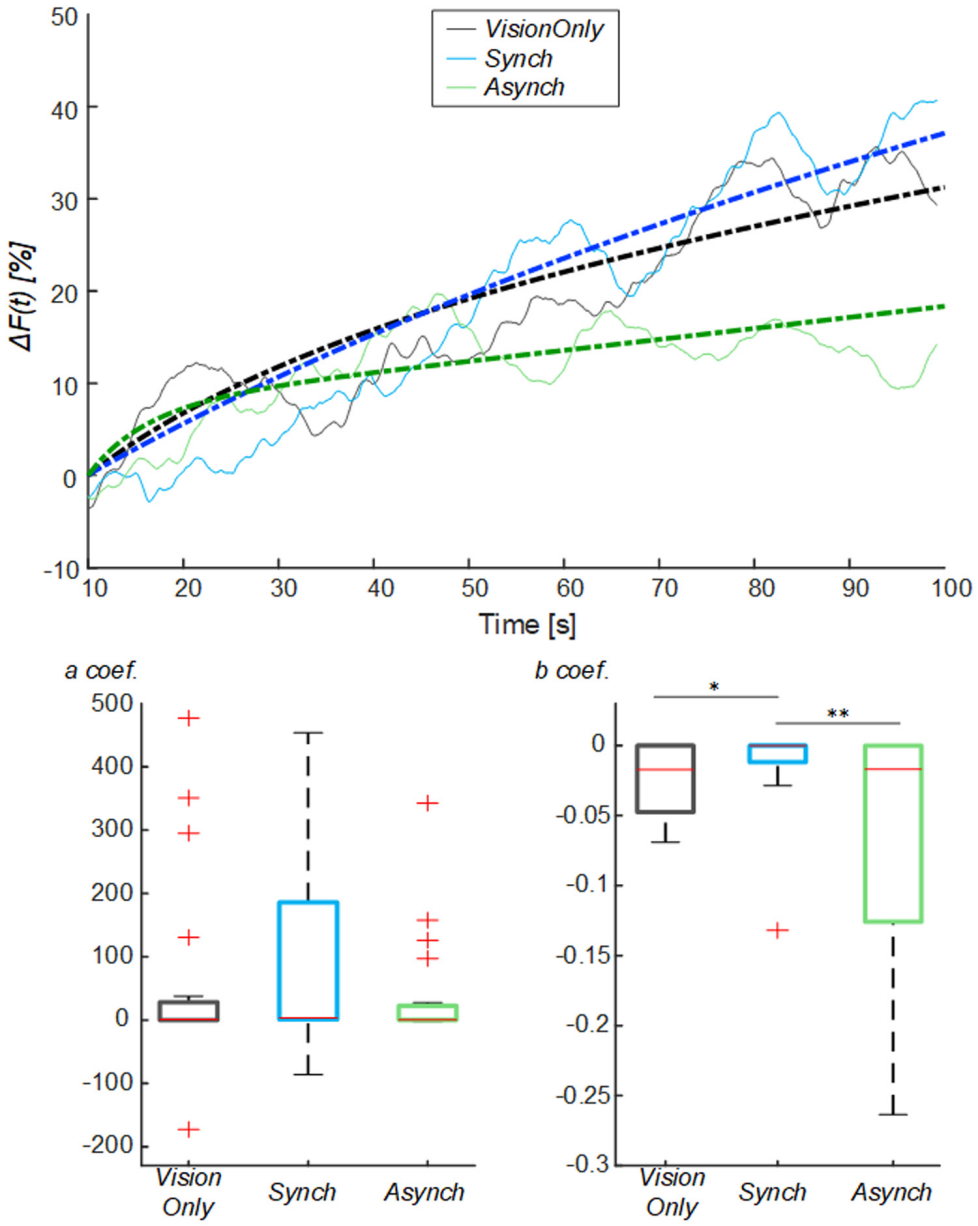
Exponential fitting of the mean blood flow data. Mean blood flow value from the drop ( *ΔF*) averaged across participants for each condition, dashed lines indicate the exponential curves that fit the data, averaged across participants (Upper panel). Box and whisker plots relative to *a* and *b* coefficients calculated in the selected intervals for *Synch* and *Asynch* and *VisionOnly* conditions (Lower panel): median (red lines), 1 ^st^ and 3 ^rd^ quartiles (box), lowest and highest values comprised within 1.5 times the interquartile range from the 1 ^st^ and 3 ^rd^ percentiles (whisker). * indicates a p-value <0.05.; ** indicates a p-value <0.01.

To avoid the influence of such drop flow value, an exponential curve was employed to fit the behavior of the blood flow from the drop identified at 10s ( Figure 5): the *a* fitting coefficient corresponds to the curve trend value; whereas the *b* coefficient corresponds to the rate to reach the trend value. The statistical analysis showed a difference in the curve fitting *b* coefficients ( *a* coefficient: χ ^2^(2, 38) = 3.70; p = 0.157; *b* coefficient: χ ^2^(2, 38) = 11.20; p = 0.004): the *b* values for the *Synch* condition were significantly higher than those of both the *VisionOnly* and *Asynch* conditions (r = 0.47, z = 2.10, p = 0.034; r = 0.49, z = 2.21, p = 0.005; respectively). This means a faster growth rate for the *Synch* condition ( Figure 5).

Both *a* and *b* coefficients were correlated to RHI index, vividness and prevalence scores (ρ >0.30, p <0.05) ( Table 2). In particular, the *b* coefficient related to the blood flow growth dynamics was more strongly correlated to questionnaire scores (ρ >0.40, p <0.05). There was no correlation with proprioceptive drift.

**Table 2.** Correlation values between fitting coefficients and illusion outcomes calculated pooling together *Synch* and *Asynch* conditions.

	Coef. *a*		Coef. *b*	
	ρ	p	ρ	p
RHI index	0.33	0.037	0.43	0.007
Vividness	0.30	0.050	0.40	0.012
Prevalence	0.36	0.024	0.41	0.010
Prop. Drift	0.15	0.372	0.09	0.576

RHI, rubber hand illusion; Prop. Drift, proprioceptive drift.

**Figure 6.**
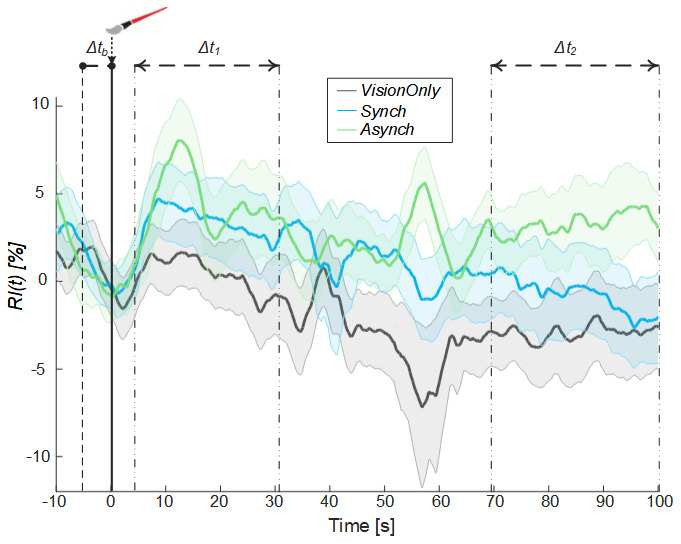
Resistance index. Resistance index ( *RI*) averaged across participants for each condition, the shaded area represents the standard error (SEM), dashed lines indicate the time intervals where the mean baseline value was calculated (Δt _b_) and the analysis performed (Δt _1_ and Δt _2_). Time=0 s is when the trial began.

Moreover, from the comparison of the systolic and diastolic variation of blood flow, a resistance index reflecting the resistance caused by microvascular bed distal to the site of measurement ^53– 55^ was calculated ( Figure 6).

A significant negative correlation between the averaged blood flow and the resistance index values was highlighted in all time intervals (Δt _1_: ρ = -0.45, p =0.005; Δt _2_.: ρ = -0.67, p = 0.003), demonstrating that the measured increase in the blood flow was tightly related to a decrease in peripheral vessels resistance of the tested limb.

## Discussion

This study was designed to investigate possible changes in the blood flow to the hand and forearm induced by the modulation of sense of limb ownership. To modulate limb ownership, we employed the RHI paradigm while the brachial artery blood flow of the ipsilateral limb was monitored.

Embodiment of a fake hand induced by the synchronous stimulation of the fake visible hand and real hidden hand of the participant ( *Synch* condition) was tested against the commonly-adopted control condition where embodiment was inhibited because the rubber hand and the participant’s own hand were asynchronously stroked ( *Asynch* condition).

Since we suspected that brush-stroking itself could have affected the flow independently from the achieved embodiment, a third condition was introduced as further control. To disentangle the effect of hand touch on blood flow from the effect of embodiment, we could have used either a condition maintaining all the factors but touch or a condition with only touch. In the former, participants should have been instructed to simply look at the fake hand, without receiving any paintbrush stimulation on the real hand ( *VisionOnly* condition), while in the latter participant’s real hand should have been brush-stroked, without vision or stimulation of the fake hand. We chose the former *VisionOnly* condition *,* because this was already employed in previous work ^43^.

The first element to note is that the adopted experimental process induced a consistent modulation of the blood flow, characterized by having different behaviors for different conditions, but small variability across participants (SEM lower than 14, 11 and 16 % for *Synch*, *Asynch* and *VisionOnly* conditions, respectively). This suggest that the designed experiment was suited to assess the targeted phenomenon.

Furthermore, looking at the average blood flow dynamics, in all conditions, we found a drop common to all conditions (reaching value significant lower than the baseline only for *Synch* and *Asynch* conditions), beginning at the start of each session, when the light was turned on in the fake hand compartment and the fake hand began to be stimulated. This drop was always followed by a progressive increase in blood flow, which reached its maximal value at the end of the stimulation period ( Figure 4).

After adopting an empirical procedure to identify time windows of signal difference, we found that in the first time-interval (5<Δt _1_≤31s), a significant difference in mean blood flow was found between the conditions with brush stimulation ( *Synch* and *Asynch*) and the *VisionOnly* condition, while no significant difference was found between *Synch* and *Asynch* conditions.

This drop was significantly different from the baseline only for *Synch* and *Asynch* conditions, while it was much less evident (not significantly different from the baseline) for the *VisionOnly*, which was the only condition without any brush-stroking applied to the real hand. This suggests that the drop was due to the initial, mostly unexpected, tactile stimulation of the hidden hand caused by the brush, regardless of whether the stroke was synchronous or asynchronous and if an illusion was achieved. A blood flow drop due to tactile stimulation has been also previously reported ^56^.

While the flow in the *VisionOnly* condition had a shallower drop (the average flow during Δt _d_ in VisionOnly is not statistically different from the baseline) and remained higher throughout the experiment, in the *Asynch* control condition the flow had a more pronounced drop due to brush-stroking and it remained lower throughout the experiment. The only condition in which the blood flow relatively increased the most, after experiencing this drop was the synchronous brush-stroking condition, which was precisely the one designed to test the effect of the fake hand embodiment.

Indeed, focusing on the second time-interval of interest (69<Δt _2_<100s), the blood flow of the asynchronous condition was significantly lower than the blood flow of the others. From the visual inspection of the change in the flow, different causes may be supposed to determine the difference between *Asynch* and *VisionOnly*, as well as between *Asynch* and *Synch*.

Compared to *Asynch*, the higher *VisionOnly* value in the second interval may be the effect of the previous milder *VisionOnly* drop (statistically different with respect to the other two conditions) or due to the absence of stroking on the hidden hand during the session.

Another possibility is that the higher *VisionOnly* value may be the effect of a slight embodiment induced by *VisionOnly*. Indeed, despite the embodiment illusion having strong dependence on the integration of coherent multisensory afferences, previous studies found that the mere sight of a fake hand placed in a congruent position could induce a mild degree of embodiment ^57, 58^, while another study did not ^59^. Since the proprioceptive drift was significantly lower in *VisionOnly* compared in *Synch*, and similar to *Asynch*, a *VisionOnly*-induced illusion should be very low.

Unfortunately, we cannot take a conclusive position on this possibility, because we did not conduct a questionnaire for *VisionOnly* for two reasons: i) this condition was introduced to control for the cause of the initial drop in flow, thus testing its embodiment was not within its original scope; ii) as previously indicated ^43^, several very important items of the Botvinick and Cohen questionnaire focus on being touched by the brush, and they lose meaning if the hand is not touched. We collected proprioceptive drift, but considering that this measure is related to different aspects of embodiment than the questionnaire ^43^, the absence of questionnaires in the *VisionOnly* condition should be considered a limitation of the study and it is envisaged for future work investigating the topic.

In contrast, *Asynch* and *Synch* conditions did not experience different magnitudes of blood flow drop. Their significantly different values can only be due to their different effect on the body representation of the hand. This finding gives credit to our hypothesis: the embodiment illusion seems to modulate the blood flow directed towards the tested forearm.

The main aim of the experiment was to investigate the modulation of the limb blood flow due to the embodiment of a fake hand and not the modulation due to tactile stimulation. To better highlight the effect of embodiment, we further corrected blood flow changes for initial drop by subtracting its value. Thus, we corrected the blood flow curves of all three conditions to have them all start from the same flow value after the drop, and modelled the subsequent flow increase with an exponential curve. The comparison of the growth rate between conditions, employing the *b* fitting parameter, showed that the synchronous illusion condition had a significantly faster growth rate than both the other conditions.

Moreover, the fitting coefficients positively correlate with most of the widely-validated measures of the illusion (i.e. RHI index, vividness and prevalence scores), confirming that the variation in the blood flow dynamics is linked to the change of embodiment level during the trials.

Generally, an increase in blood flow can be due to vasodilatation and /or an increase in cardiac output, both of which are mainly driven by the ANS.

To our knowledge, we are the first to show that embodying an artificial limb enhances the blood flow directed to the tested limb.

However, this is not the first finding involving an overactivation of the sympathetic nervous system in correlation with the illusion. Indeed, the skin conductance response, which is known to mainly be driven by the sympathetic branch of the ANS, is modulated by the illusion as well; a threat to the fake hand induces a stronger event-related skin conductance response when the hand is embodied ^30, 60– 62^. More recently, studies have demonstrated that the embodiment induced by the synchronous RHI brush-stroking, by itself enhances the spontaneous fluctuations of the skin conductance ^37, 63^.

Two tightly interconnected questions remain to be addressed: i) Is the hyperactivation of the ANS a local or a systemic response? And ii) Is the hyperactivation of the ANS due to an alert after the perceived abnormalities linked with the experimental manipulation or, more intriguingly, is it due to a mismatch between the sensory, motor and autonomic representations of the limb in the brain?

Limb vasoconstriction/dilatation is mainly affected by the ANS, specifically by the sympathetic and, to a lesser extent, by the parasympathetic activity ^64, 65^. Most systemic blood vessels, particularly those of the abdominal viscera and skin of the limbs, are constricted by the sympathetic stimulation. Contrarily, parasympathetic stimulation has almost no effect on most blood vessels, except for vasodilatation in certain restricted areas, such as in the blush response in the face ^64^.

Theoretically, the activation of the sympathetic branch of the ANS is a systemic response that recruits the whole body. However, in favor of the local response hypothesis, previous work found a selective cooling of the investigated hand compared to the contralateral hand, when ownership over the rubber hand was induced ^34, 66– 68^. For the sake of completeness, few other studies have called into question the consistency of this phenomenon ^35, 36^.

In the attempt to test the local specificity of our hypothesis, in a preliminary experiment run before this study, we tried to record the blood flow from both arms simultaneously, but unfortunately, we realized that our experimental setup was not sufficiently robust, as it was not feasible for a single experimenter to hold two probes still while accurately monitoring the blood flow of the two arms. Nonetheless, an indirect cue to the local specificity of the autonomic response can be gathered from the resistance index we extracted. Indeed, the resistance index value is determined by the arterial compliance (as opposed to the vessel’s stiffness) and vascular resistance, mainly due to the diameter of the vessels, that results in the normal loss of pulsatility as flow progresses from the arteries to the capillaries ^53– 55^.

If the ultrasonographic probe remains in the same spot, a decrease in the index is a sign of vasodilatation. The significant negative correlation between blood flow and resistance index percentage suggests that the change in the blood flow that we highlighted was tightly linked with the peripheral vessel resistance change. Together with previous work reporting no significant differences in heart rate variability between RHI illusion and control conditions ^37^, the current findings indirectly suggest a local specificity of the described phenomena.

It has been previously shown that the synchronous brush-stroking of the RHI procedure limited the increase in peripheral perfusion of the pierced skin of the hand induced by acupuncture ^69^. The reduction of a further evoked increase in skin perfusion coexists well with an increase in the general flow, being them competitive causes for a limited possible increase in the flow.

Our results suggest an enhancement of limb blood flow with fake hand embodiment. A previous study reported cooling of the RHI tested hand ^34^, probably caused by a reduced hand skin blood perfusion ^70^. How our finding would fit with the previous reported cooling of the RHI tested hand? Firstly, the cooling effect of the RHI is still matter of debate ^35, 36^; secondly, skin perfusion may well not be representative of all blood flow directed within the limb.

Indeed, we recorded brachial artery blood flow that is a cumulative measure of the flow through all the vessels placed distally with respect to the position of the probe (in our case the vessels of forearm and hand). The main part of this flow is to the muscles (59% of the total flow), followed by the bones and fat, which are relatively avascular under normal conditions (28%), and the remainder part to the skin (13%) ^71^. Blood flow recorded over the brachial artery is, hence, predominantly a measurement of the flow to the forearm and hand muscles, and may not be correlated with flow specific to the cutaneous bed where thermoregulation is performed.

With regard to the second question of whether the embodiment induced sympathetic hyperactivity is an unspecific alert response or the effect of the mismatched body image, there are conflicting hypotheses.

On one side, there is evidence for an unspecific response: a state of anxiety has been reported to raise the blood flow to the forearm at rest ^25– 27^. Indeed, an increase in the sympathetic response can enhance the heart rate and decrease the resistance of peripheral vessels in the limb, increasing its blood flow. Thus, sympathetic-induced skin vasoconstriction and muscle blood vessel dilatation may be explained as an unspecific alerting state to the defense fight or flight reaction: a preparatory adjustment for the muscular activation inseparable from these activities ^25– 27^.

On the other side, previous studies interpreted selective cooling of the tested hand and the increase in histamine reactivity after the RHI as an illusionary disembodiment and as a sign of rejection of the real hand in favor of the artificial limb ^34, 72^. In line with our finding, mounting an immune response towards a disowned limb would likely go through an increase in the blood flow towards the targeted limb. Also, this hypothesis fits with the correlation between a reduction of the skin conductance response to the threatening towards a fake hand and the loss of its self-attribution ^73^. With regard to the time course of the measured effect, the difference between illusion and control conditions was demonstrated in the 69–100s time window after the beginning of the trial, whereas previous research demonstrated a sympathetic-induced increase in the variability of the non-specific skin conductance response in the 10–55s range ^37^. This temporal mismatch between the effect seen for the skin conductance response and the effect on blood flow could be either due to the time for the flow to return to baseline after the initial drop, or to the different sudomotor and vasomotor dynamics induced by the sympathetic activation. Indeed, a temporal dissociation between responses to sympathetic activity in the skin and muscle tissue was unveiled while monitoring sympathetic neural activity during handgrip. The former abruptly raised at task onset and the latter increased slowly after a 60s latency ^74^. Despite having a cumulative faster growth rate, the synchronous illusory condition had slower initial (<30 s) dynamics. Interestingly, this behavior could be explained by the temporal dissociation of the ANS effect on skin and muscles. The more marked skin vasoconstriction elicited by a higher sympathetic activity in the *Synch* condition could slow down the rise of the blood flow in the initial phase of the trial. However, in the following phase, when the increment of the vasodilation in skeletal muscles supersedes skin vasoconstriction, the blood flow level in the *Synch* condition rapidly increases beyond the other conditions.

Previous work highlighted the possibility of increasing arousal by merely approaching a rubber hand placed in a congruent way with respect to the real hidden hand ^75^ and this effect could contribute to our outcomes. In order to assess the effect of the visual stroking per se, future studies could measure the blood flow when the stroking is delivered only on the rubber hand. Additionally, in order to assess the repeatability of our findings, an additional control condition could be performed, e.g. synchronous brush-stroking but with the fake hand placed in a incongruent position. In such way, it will be possible to confirm whether the current effect is related to changes in embodiment and not to manipulation of visuotactile stimulation synchrony.

The RHI paradigm is an easy way to evaluate embodiment. For its simplicity, low requirements and costs, it has been extremely widespread in research related to the representation of the body. However, it has several limitations ^76– 78^; one is that its outcomes are mostly assessed with subjective measures (i.e. Questionnaires). As previously suggested for the fluctuation in the non-specific skin conductance response ^37^, the blood flow may be a more objective measure of the achieved embodiment as well. Indeed, the increase in the blood flow significantly correlated with all the other employed measures designed to rate the strength of the illusion (RHI index, vividness and prevalence scores), except for proprioceptive drift. A possible interpretation for this result may be that proprioceptive drift, although correlated to the subjective measures ^44, 48^, does not measure the same aspects of the embodiment process as the questionnaire which is often a dissociated measure weighting different aspects of the embodiment process ^43^.

In conclusion, we observed that the modulation of the sense of limb ownership seems to have an impact on the blood flow directed to that limb. It is likely that the fake hand embodiment induced a sympathetic driven vasodilatation of the muscular territories downstream of the brachial artery.

Our findings seem to indicate that the modulation of body representation has an impact on the efferent branch of the ANS; whereas previous studies demonstrated that the afferent branch of the ANS contributes with the interoception to the body representation ^16– 18^. Taken together, these findings provide a further suggestion that there is a bidirectional influence between the ANS and body ownership. Interoception, led by the afferent branch of the ANS, contributes to the sense of body ownership and, in turn, this modulation may change the autonomic outflow and becomes manifested through changes of the sudomotor ^37^ and vasomotor activity. Another interesting manifestation of such bidirectional influence is that embodiment of a fake hand seems to alter real hand temperature and, in turn, the propensity of perceiving the embodiment illusion seems to be influenced by the hand temperature ^37^.

An important overlap between the brain circuits in charge of body representation and those processing interoception and controlling body temperature, heart and vessel function has been recently confirmed by several experimental, meta-analytic and theoretical works ^79– 82^, which highlighted the main role played by premotor, parietal-temporal, cingulate cortex, the amygdala and the insula.

This is the first to provide preliminary evidence that the update of the perceptual status leading to a change of a limb presence in the body representation is paralleled by an enhancement in the perfusion of the tested limb. It also opens the intriguing question of whether the reported changes are unspecific effects of an alert response regarding the whole body or, on the contrary, are specifically causally and topographically related to the limb, the representation of which was modulated. We speculated on this topic providing cues in favor of the latter. This, however, remains an extremely interesting question, a matter still open for future research.

## Data availability

### Underlying data

Mendeley Data: Embodying an artificial hand increases blood flow to the investigated limb. http://www.doi.org/10.17632/pcbtb8xfg6.3 ^51^.

This project contains the following underlying data:

- Dataset.mat (matrices of data, Matlab dataset)
- Table_MeanBF.csv (mean blood flow [20 participants X 12000 samples (from -20s to 100s at 100Hz) X 3 conditions (order: VisionOnly, Synch, Asynch)])
- Table_PD.csv (proprioceptive drift [20 participants X 3 conditions (order: Synch, Asynch, VisionOnly)])
- Table_RHIi.csv (RHI index [20 participants X 2 conditions (order: Synch, Asynch)])
- Table_RI.csv (resistance index [20 participants X 12000 samples (from -20s to 100s at 100Hz) X 3 conditions (order: VisionOnly, Synch, Asynch)])
- Table_T.csv (prevalence score [20 participants X 2 conditions (order: Synch, Asynch)])
- Table_V.csv (vividness score [20 participants X 2 conditions (order: Synch, Asynch)])
- Supplementary analysis.docx

Data are available under the terms of the Creative Commons Attribution 4.0 International license (CC-BY 4.0).
