# Supplementary material for: Embodying an artificial hand increases blood flow to the investigated limb [version 3; peer review: 2 approved]
Source: Open Res Eur. Author manuscript; Available in PMC 2022 Jun 22. (PMC7612882)
Supplement: Supplementary Information [file EMS145916-supplement-Supplementary_Information.pdf]

Open Peer Review

Current Peer Review Status: ✔ ✔

Version 3

Reviewer Report 13 June 2022

https://doi.org/10.21956/openreseurope.15902.r29080

© **2022 Peviani V.** This is an open access peer review report distributed under the terms of the Creative Commons Attribution License, which permits unrestricted use, distribution, and reproduction in any medium, provided the original work is properly cited.

Valeria Peviani
Department of Neuroscience, Max Planck Institute for Empirical Aesthetics, Frankfurt, Germany

The authors answered my questions.

Competing Interests: No competing interests were disclosed.

I confirm that I have read this submission and believe that I have an appropriate level of expertise to confirm that it is of an acceptable scientific standard.

Reviewer Report 04 May 2022

https://doi.org/10.21956/openreseurope.15902.r29079

© **2022 Ehrsson H et al.** This is an open access peer review report distributed under the terms of the Creative Commons Attribution License, which permits unrestricted use, distribution, and reproduction in any medium, provided the original work is properly cited.

Henrik Ehrsson
Department of Neuroscience, Brain, Body and Self Laboratory, Karolinska Institutet, Solna, Sweden
Renzo Lanfranco
Department of Neuroscience, Brain, Body and Self Laboratory, Karolinska Institutet, Solna, Sweden

I think the authors have addressed our points well and that the manuscript can now be accepted. They have handled our previous concerns regarding methods and analyses well, and the conclusions and discussion are now more balanced and nuanced.

Competing Interests: No competing interests were disclosed.

We confirm that we have read this submission and believe that we have an appropriate level of expertise to confirm that it is of an acceptable scientific standard.

Version 2

Reviewer Report 21 December 2021

https://doi.org/10.21956/openreseurope.15369.r27976

© **2021 Ehrsson H et al.** This is an open access peer review report distributed under the terms of the Creative Commons Attribution License, which permits unrestricted use, distribution, and reproduction in any medium, provided the original work is properly cited.

Henrik Ehrsson
Department of Neuroscience, Brain, Body and Self Laboratory, Karolinska Institutet, Solna, Sweden
Renzo Lanfranco
Department of Neuroscience, Brain, Body and Self Laboratory, Karolinska Institutet, Solna, Sweden

Editorial Note 4th May 2022:
This report has been updated to credit Renzo Lanfranco as a co-reviewer at the request of the primary reviewer.

Recommendation: Not approved. Major revisions are needed.

<u>Summary</u>
As I wrote in my original report, this study addresses and fascinating a novel question: does the rubber hand illusion (RHI) change the arm's blood flow? The authors present evidence from one experiment indicating that this may be the case, at least after a delay, based on contrasting synchronous and asynchronous visuotactile conditions. In principle, I agree with the authors that this study should be reported because the research question is original, and the findings are promising. However, it is unfortunate that the authors did not conduct further control and replication experiments as I suggested. In their response, the authors explain that they no longer have access to the equipment and expertise needed to carry out additional experiments. However, this leaves us with only one experiment that presents rather inclusive evidence. On the positive side, the authors implemented an extra analysis of the blood flow time series based on permutation testing that overcomes the limitations of arbitrarily dividing the data into three periods. I also asked for various changes in the literature review and interpretation of the results, and the authors have done a pretty good job in this regard, and they have implemented many relevant changes that have improved the manuscript. Nevertheless, I have read the whole manuscript carefully again to determine if the text and data we have at hand meet the standard expected from a scientific article. I hoped I could be more positive at this point, but unfortunately,

I still think the article needs more work before recommending that it be accepted.

Main points

1. The methods and statistical analysis still need to be described and motivated in more detail, as I outline below. Some information needed to replicate the study is missing, and some information motivating the statistical procedures need to be clarified. Since this study is based on results from a single experiment, it is particularly important the methods and results from that experiment are solid, well-described, and of high quality (see below).

2. The conclusions are still overstated I some places, and the authors must be more cautious and nuanced when formulating their conclusions (see examples below). In my opinion, the current study presents preliminary evidence that the rubber hand illusion changes the arm's blood flow, but future experiments are needed to verify this observation. However, it was good to see that the authors acknowledged this in the revised discussion.

3. In my opinion, we do not know for sure if the rubber hand illusion increases the arm's blood flow relative to some relevant baseline, or if the blood flow is actually reduced in both the synchronous and the asynchronous conditions, but this suppression just happens to be more sustained in the asynchronous condition for some unknown reason. The baseline before the visuotactile stimulation is probably too short for a reliable and stable assessment of the baseline (see below). The blood flow appears lower in the asynchronous condition compared to the vision only control, but the vision only condition and the synchronous condition display similar blood flow in the critical third period. The authors theorize an effect of tactile stimulation could have influenced the blood flow (or lack of such tactile stimulation in the vision only condition), and this may be reasonable speculation, but problematically, no data is presented to support this view. If you have pilot data demonstrating such a drop in the current setup, I think it could be useful to add them as supplementary material. In a future study, it could be useful to have a vision only condition with tactile stimulation of the real hand to better understand the basic tactile effect. I think the authors could discuss these issues a little bit more in the Discussion (for example on page 11).

4. In my opinion, the authors still need to work a little more on the literature review. Some issues are not presented in a sufficiently balanced manner, for example, the potential changes in skin temperature or other objective measures such as the proprioceptive drift.

5. The structure of the article and language needs to be improved. There are too many grammatical mistakes and awkward phrasing. The paper's level of English doesn't meet academic standards, in my opinion. I would recommend that the text is sent to professional proofreading. Moreover, the sections are somewhat poorly organized: e.g., there's information that belongs to the Methods section in the Results section.

Specific points concerning methods and results

1. The authors mention that the sampling rate is 100Hz. I'm not familiar with blood flow measures, but assuming this is fine, I don't understand why you say "for each condition, about 92 measures of each parameter were recorded…"; are the authors referring to the number of data points in an epoch? To the number of epochs (after segmentation)? The authors need to explain this more clearly so the reader can judge whether having

"approximate" numbers of these by-condition measures is fine.

2. You mention that each session lasted 30 minutes – are you talking about blocks or whole sessions? More importantly, how many trials did their experiment involve? How many epochs per trial? How many trials were rejected, and why?

3. The authors don't provide a rationale for the baseline they determined. They state that they used a "moving average 5s window to eliminate high-frequency noise." But the authors also say they used the last 5s of the baseline interval, averaged. They don't explain the motivation behind those decisions. I don't have experience measuring blood flow-based time series, but a baseline of 5s for 100s conditions seems insufficient to me, especially if there are so many factors that can affect blood flow (as the authors briefly mention).

4. By looking at the main blood-flow timeline plot, I can see that the last 5s before the trial look stable, with similar values between conditions. Perhaps they chose those 5s based on this. However, in my opinion, this approach violates the main purpose of baseline-corrections: that the time series data don't vary significantly between conditions when there's no event or trial taking place. Any activity measured before the event or trial is considered spontaneous or random and therefore it can be averaged and subsequently subtracted from the rest of the trial. What the authors did, choosing the last 5s (instead of using full 10s period), may have confounded their results.

5. The authors used cluster-based permutations to determine significant clusters, and I really like this idea. However, they don't explain why they used 250 permutations. That number seems relatively low to me (for example, in EEG/EMG studies, many more permutations are used). Please explain the choice of 250 permutations and cite other papers that have used a similar number.

6. The authors used Tukey-Kramer corrections for multiple comparisons for the repeated-measures ANOVAs' post hoc t-tests. As far as I understand, this correction assumes that observations are independent. Therefore, it's not appropriate for a within-subjects model such as a repeated-measures ANOVA. In their experiment, the participants went through the three conditions, so the observations are not independent.

7. The authors report having found a drop in blood flow values during the first 10s, for which they applied another correction. However, the authors don't explain the motivation for this – they already applied baseline correction, so why apply another correction of similar nature? Did this drop in blood flow differ between conditions? I think the authors should provide more details; it looks like a random decision as it is explained now.

8. For this same correction, the authors report having used one-sample t-tests to determine whether the drop was significantly different from their original baseline. But the baseline wasn't zero so why did they assume the baseline as zero? This seems quite strange to me. Also, were those t-tests corrected for multiple comparisons? I assume they ran one per condition, but they don't say.

9. To compare the coefficients of the exponential function that the authors used to fit the data (they don't explain why they used that one), they used the Friedman Test. Why? Didn't the

data have a normal distribution? They again used the Tukey-Kramer correction. I think that they should probably have used the Dunn-Bonferroni correction to correct for multiple comparisons done with the Friedman Test.

10. The important significant interaction between time and condition, which is the basis for the post-hoc tests and the author's main claims, was p=0.049. Thus, it is very important that all pre-processing decisions are well motivated and appropriately implemented to ensure that this significant interaction is solid.

11. I could not find where the authors describe where the different blocks were counterbalanced, assuming they were. Given that all conditions were associated with a drop in blood flow during the first 10s (although I could not find that the authors mention whether the conditions differed in this respect or not), it seems important that the three conditions should be properly counterbalanced to avoid order effects.

<u>Literature review and interpretation</u>

1. Given the uncertainties discussed in this review, I recommend that the authors modify the conclusions to fit the results better. For example, instead of stating that your results "have demonstrated" I would say that your results "suggest" or that "our preliminary evidence indicates". Moreover, in the discussion, the authors unnecessary talk about how "the blood flow dramatically increased", and that this increase occurred "precisely" in the condition designed to test the effect. However, this study only uses one useful control condition, the timing of the blood flow changes was not predicted, and I do not understand what is so dramatic about the effect? There are many examples in the abstract and discussion where I think the authors overstate their findings' conclusiveness. I recommend modifying the text to present a more accurate and nuanced description of the results and their interpretation.

2. In on one of the discussion paragraphs, you talk about "the second-time" interval, but in the most recent version of the manuscript, you have three, right?

3. I think you downplay the negative findings too much when reviewing the literature on temperature changes (discussion). In my opinion, we are currently not sure if the arm's skin temperature changes during the RHI. The de Haan study was quite convincing, I think. There exist other published studies with negative findings that you do not cite. We have measured skin temperature in several pilot studies in our lab and never observed significant and systematic changes. So how do your results fit with the view that the skin temperature might not change during the RHI? Maybe the authors should also consider this scenario in the text?

4. In the discussion, you mention studies that found a down-regulation of somatosensory evoked potentials, but you should probably also cite studies that did not, for example, the well-conducted EEG study by Rao and Kayser 2017 (see also the ECoG findings from S1 hand area published by Guterstam *et al* 2019 Cerebral Cortex). Also, I do not fully understand how this early modulation of somatosensory evoked responses is believed to be related to the blood flow changes in the arm. Is this discussion not too speculative?

5. In a paragraph towards the end of the discussion, the authors state that the RHI "lacks objective measures". The authors should rephrase this sentence as it is not a correct description of the literature. Several different objective measures have been proposed and

replicated in previous RHI studies. I also think that your statement “the proprioceptive drift is an often dissociated measure” needs to be modified because many previous studies have reported significant differences in proprioceptive drift between the synchronous and asynchronous conditions. Moreover, it is not uncommon to find reports of significant correlations between drift and subjective illusion (e.g., Abdulkarim & Ehrsson 2016; Kalckert & Ehrsson 2014).

6. The title says, “increases blood flow”, and this is also how you interpret the results. But as I wrote above, can we be entirely sure that the blood flow does not decrease in both the synchronous and the asynchronous conditions compared to some relevant baseline, but just so to varying degrees after a certain delay? Maybe “changes blood flow”, “time-varying changes in blood flow” or “relative increase in blood flow” would be a safer and more appropriately cautiously worded title given the data?

7. You conclude that your study provides “further proof that there is a bidirectional influence between ANS and body ownership”. However, this study has not tested bidirectionality, so this conclusion is incorrect. I found this part unnecessarily speculative.

References
Abdulkarim Z, Ehrsson HH. No causal link between changes in hand position sense and feeling of limb ownership in the rubber hand illusion. Atten Percept Psychophys. 2016 Feb;78(2):707-20. doi: 10.3758/s13414-015-1016-0.

Guterstam A, Collins KL, Cronin JA, Zeberg H, Darvas F, Weaver KE, Ojemann JG, Ehrsson HH. Direct Electrophysiological Correlates of Body Ownership in Human Cerebral Cortex. Cereb Cortex. 2019 Mar 1;29(3):1328-1341. doi: 10.1093/cercor/bhy285.

Kalckert A, Ehrsson HH. The moving rubber hand illusion revisited: comparing movements and visuotactile stimulation to induce illusory ownership. Conscious Cogn. 2014 May;26:117-32. doi: 10.1016/j.concog.2014.02.003.

Rao IS, Kayser C. 2017. Neurophysiological Correlates of the Rubber Hand Illusion in Late Evoked and Alpha/Beta Band Activity. Front Hum Neurosci 11: 377

References
1. Abdulkarim Z, Ehrsson HH: No causal link between changes in hand position sense and feeling of limb ownership in the rubber hand illusion.*Atten Percept Psychophys*. 2016; **78** (2): 707-20 PubMed Abstract | Publisher Full Text
2. Guterstam A, Collins K, Cronin J, Zeberg H, et al.: Direct Electrophysiological Correlates of Body Ownership in Human Cerebral Cortex. *Cerebral Cortex*. 2019; **29** (3): 1328-1341 Publisher Full Text
3. Kalckert A, Ehrsson HH: The moving rubber hand illusion revisited: comparing movements and visuotactile stimulation to induce illusory ownership.*Conscious Cogn*. 2014; **26**: 117-32 PubMed Abstract | Publisher Full Text
4. Rao IS, Kayser C: Neurophysiological Correlates of the Rubber Hand Illusion in Late Evoked and Alpha/Beta Band Activity.*Front Hum Neurosci*. 2017; **11**: 377 PubMed Abstract | Publisher Full Text

Competing Interests: No competing interests were disclosed.

We confirm that we have read this submission and believe that we have an appropriate level of expertise to state that we do not consider it to be of an acceptable scientific standard, for reasons outlined above.

Author Response 11 Apr 2022

Marco D'Alonzo, Campus Bio-Medico University of Rome, via Alvaro del Portillo, 5, Rome, Italy

The new revised version of the manuscript entitled "Embodying an artificial hand increases blood flow to the investigated limb" is updated. We wish to thank again the Reviewers for the time spent on our manuscript. We carefully considered the Reviewers' observations and suggestions that helped us to refine the quality of the draft. Point-by-point replies (A.) to the Reviewers' comments (numbered and in italics) are provided below, and changes to the text are underlined here. The manuscript has been kindly revised by an English mother-tongue scientist (Dr. Silvia Isabella) who we deeply thank. We look forward to your response.
Sincerely, on behalf of all co-authors
Marco D'Alonzo and Giovanni Di Pino

As I wrote in my original report, this study addresses and fascinating a novel question: does the rubber hand illusion (RHI) change the arm's blood flow? The authors present evidence from one experiment indicating that this may be the case, at least after a delay, based on contrasting synchronous and asynchronous visuotactile conditions. In principle, I agree with the authors that this study should be reported because the research question is original, and the findings are promising. However, it is unfortunate that the authors did not conduct further control and replication experiments as I suggested. In their response, the authors explain that they no longer have access to the equipment and expertise needed to carry out additional experiments. However, this leaves us with only one experiment that presents rather inclusive evidence. On the positive side, the authors implemented an extra analysis of the blood flow time series based on permutation testing that overcomes the limitations of arbitrarily dividing the data into three periods. I also asked for various changes in the literature review and interpretation of the results, and the authors have done a pretty good job in this regard, and they have implemented many relevant changes that have improved the manuscript. Nevertheless, I have read the whole manuscript carefully again to determine if the text and data we have at hand meet the standard expected from a scientific article. I hoped I could be more positive at this point, but unfortunately, I still think the article needs more work before recommending that it be accepted.
A: Dear Prof. Ehrsson, thank you again for the time spent on the manuscript. We took into account all your inputs and we implemented all of them in this new version of the manuscript.

MAIN POINTS
1. The methods and statistical analysis still need to be described and motivated in more detail, as I outline below. Some information needed to replicate the study is missing, and some information motivating the statistical procedures need to be clarified. Since this study is based on results from a single experiment, it is particularly important the methods and results from that experiment are solid, well-described, and of high quality (see below).
A: On basis of the reviewer's suggestions reported in the following comments, we made a

huge work editing our manuscript and explaining in more details the employed methods. Additionally, the new methods and data analysis after the reviewer's input allowed us to check the whole information of our manuscript and correct possible typos. For instance, an additional analysis using different longer baselines to normalize data was also performed. See also the responses to specific points in Methods and Results and answer to question 4 of the main points.

2. The conclusions are still overstated in some places, and the authors must be more cautious and nuanced when formulating their conclusions (see examples below). In my opinion, the current study presents preliminary evidence that the rubber hand illusion changes the arm's blood flow, but future experiments are needed to verify this observation. However, it was good to see that the authors acknowledged this in the revised discussion
A: As suggested by the reviewer, our conclusions have been nuanced, as can be seen by the following edits.

- Abstract: "Conclusions: These findings suggest that modulating the representation of a body part impacts its blood perfusion."

- Plain language summary: "By exploring the brachial artery blood flow recording during the rubber hand illusion paradigm, we observed that modulating the belonging of a body part to the body representation seems to increase its perfusion, through a sympathetic-driven downstream vasodilatation."

- Discussion: "The first element to note is that the adopted experimental process induced a consistent modulation of the blood flow..."
  "While the flow in the *VisionOnly* condition had a more shallow drop (the average flow during Δt_d in VisionOnly is not statistically different from the baseline) and remained higher throughout the experiment, in the *Asynch* control condition the flow had a more pronounced drop due to brush-stroking and it remained lower throughout the experiment. The only condition in which the blood flow relatively increased the most after experiencing this drop was the synchronous brush-stroking condition, which was precisely the one designed to test the effect of the fake hand embodiment."
  "In contrast, *Asynch* and *Synch* conditions did not experience different magnitudes of blood flow drop. Their significantly different values can only be due to their different effect on the body representation of the hand. This finding gives credit to our hypothesis: the embodiment illusion seems to modulate the blood flow directed towards the tested forearm."
  "To our knowledge, we are the first to show that embodying an artificial limb enhances the blood flow directed to the tested limb."
  "Our results suggest an enhancement of limb blood flow with fake hand embodiment."
  "In conclusion, we observed that the modulation of the sense of limb ownership seems to have an impact on the blood flow directed to that limb."
  "This study is the first to bring preliminary evidence that the update of the perceptual status leading to a change of a limb presence in the body representation is paralleled by an enhancement in the perfusion of the tested limb." "Our findings seem to indicate that the modulation of body representation has an impact on the efferent branch of the ANS, whereas previous studies demonstrated that afferent branch of

ANS contributes with the interoception to the body representation [16]-[18]. Taken together, these findings provide a further suggestion that there is a bidirectional influence between the ANS and body ownership. Interoception, led by the afferent branch of the ANS, contributes to shape the sense of body ownership and, in turn, this modulation may change the autonomic outflow and becomes manifested through changes of the sudomotor and vasomotor activity."

3. In my opinion, we do not know for sure if the rubber hand illusion increases the arm's blood flow relative to some relevant baseline, or if the blood flow is actually reduced in both the synchronous and the asynchronous conditions, but this suppression just happens to be more sustained in the asynchronous condition for some unknown reason.

A. The statement "Embodying an artificial hand increases blood flow to the investigated limb" may be intended in two ways: as compared 1) to a control condition or 2) to the previous flow. In our experiment both possibilities seem to be true. Indeed, in our work, we found that:

1. The blood flow value for the condition of fake hand embodiment was higher with respect to the control in Δt_2 interval, hence, there is an increase of the blood flow with respect to a no embodiment condition. Moreover, calculating the blood flow value from the drop (ΔF) (figure 5), the fitting curve values for Synch condition resulted higher than for both the control conditions in the last 30s of stimulation.
2. Considering as baseline the signal before the start of stimulation, the flow for Synch condition in Δt_2 is still averagely higher than the baseline (see figure 4 in the manuscript and A1). This excludes the hypothesis that "*the blood flow is actually reduced in both the synchronous and the asynchronous conditions*",given that the flow is instead higher than the baseline in Synch.

3. (continued) The baseline before the visuotactile stimulation is probably too short for a reliable and stable assessment of the baseline (see below).

A. We started to acquire blood flow 20 seconds before the light was turned on. Before starting to consider the signal suited for baseline, we wanted to be sure that the signal had time to be stable. From visual inspection, we estimated that 5 out of 20 seconds was the best compromise between stabilization after starting the acquisition and stability of the signal before starting the experiment. This was better explained in Materials and Methods section: "This baseline duration was selected based on visual inspection, where 5 s duration was the best compromise between the stability of the signal before starting the experiment and after starting acquisition." However, the concern about the short length of the baseline employed to normalize the dataset prompted us to make an additional analysis to check whether the findings relative to short baseline change using longer baseline. In order to control for such possible bias, and to clear reviewer's and our doubts on result robustness, we tested multiple baseline lengths (16): from 5 s to 20 s with step of 1 s. Figure A1 shows the blood flow with the change of the baseline length, highlighting minimal differences with respect to the 5 s duration. We performed statistical analysis to identify difference among conditions on two different time intervals in similar way to what presented in Methods section of the manuscript. The intervals were identified calculating a point-by-point ANOVA among the different conditions in all the datasets normalized for the different baseline. The intervals of time points, where ANOVA p-values were lower than 0.05 and common to all the normalization baselines, were employed as the analysis intervals. We identified two intervals: one between 16 and 28 s (Δt_1) and the other between 74 and 100 s (Δt_2). The

obtained results (p-values) for rmANOVA run on the mean blood flow of the two “significant” intervals and the post hoc analyses are showed for the different baseline lengths on FigureA2 and A3. p-values are similar among them and to those ones found for shorter baseline duration, suggesting that the selection of shorter baseline interval did not affect our main outcomes. We suggest reporting this further control for different baseline in Supplementary material, because it may divert reader’s attention from the main work message. However, if Prof Ehrsson’s opinion is to insert it in the main text we are available to comply with his request.

The figures discussed can be found here.

3. (continued) The blood flow appears lower in the asynchronous condition compared to the vision only control, but the vision only condition and the synchronous condition display similar blood flow in the critical third period. The authors theorize an effect of tactile stimulation could have influenced the blood flow (or lack of such tactile stimulation in the vision only condition), and this may be reasonable speculation, but problematically, no data is presented to support this view. If you have pilot data demonstrating such a drop in the current setup, I think it could be useful to add them as supplementary material.

A. The blood flow value in the tactile stimulation conditions (i.e. Synch and Asynch) significant lower than no tactile stimulation condition (i.e. VisionOnly) during Δt_1 interval provides already preliminary evidence that the drop is due to the presence of tactile stimulation and, hence, confirm the effect of tactile stimulation on the blood flow. When we state that we run a pilot experiment, just few participants were tested without the VisionOnly condition; we saw the common drop, decided to add VisionOnly and start the experiment again. Thus, those data cannot add anything more to what can be seen and tested statistically here. Additionally, it is worth noting that other cited work [54] reported that tactile stimulation can generate a drop in blood flow. Regarding reviewer’s statement that “no data is presented to support that the reason behind the similar VisionOnly and Synch blood flow in the critical last period is the VisionOnly shallower drop” we respectfully disagree. Indeed, considering that the growth rate of the curve linking drop is significantly higher for Synch compared to VisionOnly, a similar Δt_2 blood flow value may only be due to a different drop, milder for VisionOnly.

3. (continued...) In a future study, it could be useful to have a vision only condition with tactile stimulation of the real hand to better understand the basic tactile effect. I think the authors could discuss these issues a little bit more in the Discussion (for example on page 11).

A. As regards the selection of the control condition, we decided to use the VisionOnly condition, because employed in previous study[43]. Probably, as the reviewer suggests, introducing a condition with only brush-stroking of the real participant’s hand (i.e. only real hand brushstroking procedure) could have been an alternative. We proposed in discussion the only real hand brushstroking procedure as alternative control condition to evaluate also the effect of the touch on the real hand. “To disentangle the effect of hand touch on blood flow from the effect of embodiment, we could have used either a condition maintaining all the factors but touch or a condition with only touch. In the former, participants should have been instructed to simply look at the fake hand, without receiving any paintbrush stimulation on the real hand (*VisionOnly* condition), while in the latter participant’s real hand should have been brush-stroked, without vision or stimulation of the fake hand. We chose

the former *VisionOnly* condition, because this was already employed in previous work [43]."

4. In my opinion, the authors still need to work a little more on the literature review. Some issues are not presented in a sufficiently balanced manner, for example, the potential changes in skin temperature or other objective measures such as the proprioceptive drift.
A. Following the suggestions of the reviewer, in the manuscript, we rephrased the discussion sentences to present in more balanced way the physiological and behavioral measures employed to measure embodiment: Proprioceptive drift: "Indeed, the increase in the blood flow significantly correlated with all the other employed measures designed to rate the strength of the illusion (RHI index, vividness and prevalence scores), except for proprioceptive drift. A possible interpretation for this result may be that proprioceptive drift, although correlated to the subjective measures [44, 48], does not measure the same aspects of the embodiment process as the questionnaire [43]" Skin temperature: "A previous study reported cooling of the RHI tested hand [34], probably caused by a reduced hand skin blood perfusion [68]. How our finding would fit with the previous reported cooling of the RHI tested hand? Firstly, it is worth to say that the cooling effect of the RHI is still matter of debate [35, 36]; secondly, skin perfusion may not be representative of all blood flow directed within the limb." It is also worth to say that, in Introduction, we already acknowledged the mixed results found for skin temperature in literature: "The occurrence of the RHI results in disownership and a decrease in the skin temperature of the real hand [34] , but the consistency of such findings is still under debate [35, 36] ."

5. The structure of the article and language needs to be improved. There are too many grammatical mistakes and awkward phrasing. The paper's level of English doesn't meet academic standards, in my opinion. I would recommend that the text is sent to professional proofreading.
A. The manuscript has been carefully and substantially reviewed by an English mother-tongue scientist (Dr Silvia Isabella), who we deeply thank.

5. (continued) Moreover, the sections are somewhat poorly organized: e.g., there's information that belongs to the Methods section in the Results section.
A. We read thoroughly the manuscript and we found in the results the description of the order of conditions, which we promptly moved to the Methods session. Moreover, we agree with Prof Ehrsson that placing in the Results section participants' information it is uncommon. However, this was done to comply with an explicit request of the editorial team of Open Research Europe (in the version of the manuscript returned with comments of the editorial team they stated: "We would recommend that details of participants and demographics are moved to the Results section").

SPECIFIC POINTS CONCERNING METHODS AND RESULTS
1. The authors mention that the sampling rate is 100Hz. I'm not familiar with blood flow measures, but assuming this is fine, I don't understand why you say "for each condition, about 92 measures of each parameter were recorded…"; are the authors referring to the number of data points in an epoch? To the number of epochs (after segmentation)? The authors need to explain this more clearly so the reader can judge whether having "approximate" numbers of these by-condition measures is fine.
A. Sorry if the explanation was not completely clear. The raw blood signal was recorded at 100 Hz frequency. The devices extracted each 1.3 s from the raw signal three parameters

(i.e. mean, systolic and diastolic flow). These parameters were, hence, calculated from 130 samples of the raw signal. For such reason, 92 measures were recorded from 120 s of recording (120/1.3 = 92 times). "Every 1.3s (i.e. using 130 samples each time), from the blood flow data the device calculated and saved for further analysis three parameters: the mean blood flow, the peak of systolic flow and the peak of the diastolic blood flow. Once the blood flow was stable, it was recorded for 120s, from 20s before the compartment's lighting was turned until 100s after it. For each condition, 92 measures (i.e. 120s/ 1.3s = 92 times) of each parameter were recorded (77 if considering the period when compartment's lighting was on)."

2. You mention that each session lasted 30 minutes – are you talking about blocks or whole sessions? More importantly, how many trials did their experiment involve? How many epochs per trial? How many trials were rejected, and why?

A. We would like to thank the reviewer for the suggestion. Now, we specified what the experimental duration includes: "The overall experimental session lasted about 30 minutes for each participant. This included reading and signing informed consent, participant setup, probe positioning on the artery, administration of the three experimental conditions (one time for each condition) and, for each condition, performing the proprioceptive measures and filling out the questionnaire." It is not clear what the reviewer intends for trial and epoch in our experimental context. For each participant, each condition was repeated one time. In case of condition with brushstroking, the number of brush was about 100 ("The tactile stimulation was delivered at a frequency of 1Hz."). No data or trial was excluded from the analysis. This is, now, specified:"No trial or data was rejected or excluded from the analysis."

3. The authors don't provide a rationale for the baseline they determined. They state that they used a "moving average 5s window to eliminate high-frequency noise." But the authors also say they used the last 5s of the baseline interval, averaged. They don't explain the motivation behind those decisions. I don't have experience measuring blood flow-based time series, but a baseline of 5s for 100s conditions seems insufficient to me, especially if there are so many factors that can affect blood flow (as the authors briefly mention). By looking at the main blood-flow timeline plot, I can see that the last 5s before the trial look stable, with similar values between conditions. Perhaps they chose those 5s based on this. However, in my opinion, this approach violates the main purpose of baseline-corrections: that the time series data don't vary significantly between conditions when there's no event or trial taking place. Any activity measured before the event or trial is considered spontaneous or random and therefore it can be averaged and subsequently subtracted from the rest of the trial. What the authors did, choosing the last 5s (instead of using full 10s period), may have confounded their results.

A. We used a moving average window as low pass filter to eliminate high-frequency noise. We did not want to attenuate the blood flow frequency of interest (i.e. 0.02-0.05 Hz [53]) and, for such reason, cutoff frequency higher than 0.05 Hz has to be selected. The use of 5s window, corresponding to a cutoff of ~ 0.1 Hz, is in line with previous literature (Ferreira et al., 2006) and should not affect the frequency of interest. This was specified in Materials and Methods section and the new reference added: "The use of this moving average window eliminates high frequency noise without attenuating the frequency of interest [87] (i.e. 0.02-0.05 Hz [53])." As regards the duration of the baseline, the reason of using 5 s duration baseline is now explained in Methods section. An additional analysis was also performed to

check whether the findings relative to short baseline change using longer baseline. However, the findings obtained for different length of the baseline are similar among them, demonstrating that the selection of short interval should not affect the main outcomes. See also comment 4 of the reviewer's Main points.

4. The authors used cluster-based permutations to determine significant clusters, and I really like this idea. However, they don't explain why they used 250 permutations. That number seems relatively low to me (for example, in EEG/EMG studies, many more permutations are used). Please explain the choice of 250 permutations and cite other papers that have used a similar number.
A. In literature (Cohen, "Analyzing neural time series data"), it is also reported that 200-500 iterations may be sufficient to determinate significant clusters. However, this depends to quality of the recorded data (noise level) and, for most applications, it is better to use at least 1000 iterations. Following your suggestion, we performed the analysis by increasing the number of permutations (1000 iterations), and the results did not change. The reference was added and Methods section changed: "In particular, we employed 1000 permutations in *clustersize-based permutation testing and percentile of mean cluster sum* as method to define the threshold distinguishing between "significant" and "non-significant" clusters [86]."

5. The authors used Tukey-Kramer corrections for multiple comparisons for the repeated-measures ANOVAs' post hoc t-tests. As far as I understand, this correction assumes that observations are independent. Therefore, it's not appropriate for a within-subjects model such as a repeated-measures ANOVA. In their experiment, the participants went through the three conditions, so the observations are not independent.
A. We thank the reviewer for noting it. As the reviewer correctly said, Tukey-Kramer correction is more appropriated for independent observations post-hoc analysis. We made a mistake in reporting the method. Actually, we used a Tukey's Honest Significant Difference procedure. The function in the software that we used (multcompare function in Matlab) employed the same property name for both the methods (the procedure change on basis of the type of data), the error is, hence, caused by this homonymy. The sentences were corrected. ".. a Tukey's honestly significant difference test was employed as post-hoc analysis." ".. we made two separate post-hoc analyses using a Tukey's honestly significant difference test"

6. The authors report having found a drop in blood flow values during the first 10s, for which they applied another correction. However, the authors don't explain the motivation for this – they already applied baseline correction, so why apply another correction of similar nature? Did this drop in blood flow differ between conditions? I think the authors should provide more details; it looks like a random decision as it is explained now.
A. Main aim of the work was to study how embodiment (i.e. Synch vs Asynch) would have impact on blood flow. In the flow during both Synch and Asynch conditions we found a confounding effect, an initial prominent drop of the signal due to tactile stimulation of the hand, independently if tactile stimulus was delivered synchronously or asynchronously with the visual stimuli. This suggested that the drop was not related to embodiment, thus out of our main scope. We, then, designed a condition without touch to demonstrate that the drop was due to this factor, where the drop was minimal. Thus, to disentangle the mere effect of touch on blood flow and, instead, highlight the effect of visuo-tactile synchrony (embodiment) we corrected the signal from the drop. As specified in the Results section,

normalized drop values significantly lower than 0 were obtained for the Synch and Asynch conditions and no for VisionOnly one. This finding together with significant difference among conditions obtained in Δt_1 interval (i.e. the interval including the drop) seems to highlight a different behavior of the blood flow in drop intervals among conditions, explaining the use of the drop correction. However, an additional statistical analysis of blood flow values in the drop interval among different conditions was also performed in order to confirm such difference. A significant difference was obtained. The use of drop correction is now better explained in Methods section: “$\bar{F}$ (Δt_d) (i.e. the drop of the signal) was calculated as blood flow value averaged on a 10s window centered 10s after the beginning of each trial (i.e. Δt_d = [5s, 15s]). For each condition, the signal drop value was analyzed to assess whether it was significantly lower than the baseline (i.e. 0 value), by using a one-sample t-test. Additionally, a repeated measure ANOVA was employed to detect difference between conditions. Considering the obtained significant difference, we corrected the blood curves (relative shift along the y-axis) of all the three conditions to make all of them starting from the same flow value after the drop and analyze the blood flow increase independently to the drop.” The results about the statistical analysis are specified in Results section: “The values of the drop were (mean ± st. dev.): -18.4 ± 18.3% and -24.8 ± 16.4% for, *Synch* and *Asynch* condition respectively, while just -3.4 ± 16.9%, for *VisionOnly*. A significant difference among conditions was also observed ($F(2, 38) = 8.48$, $p = 0.002$).”

7. For this same correction, the authors report having used one-sample t-tests to determine whether the drop was significantly different from their original baseline. But the baseline wasn’t zero so why did they assume the baseline as zero? This seems quite strange to me. Also, were those t-tests corrected for multiple comparisons? I assume they ran one per condition, but they don’t say.

A. Considering that the blood flow raw data were normalized with respect to the baseline for each condition and participant (this means also subtracting the baseline to the blood flow signal on basis of the equation 3 in the manuscript), if the drop raw value is lower than the baseline one, the normalized drop value has to be lower than 0. Therefore, normalized drop significantly lower than 0 means that baseline raw value is significantly higher than drop value. The t-tests were run one for each condition. This was better specified in the text: “where $\bar{F}$ (Δt_d) (i.e. the drop of the signal) was calculated as blood flow value averaged on a 10s window centered 10s after the beginning of each trial (i.e. Δt_d = [5s, 15s]). For each condition, the signal drop value was analyzed to assess whether it was significantly lower than the baseline (i.e. 0 value), by using a one-sample t-test.” It is important to note that the correction is not necessary for these t-tests because the samples are not compared among them and, hence, these tests are not multiple comparisons.

8. To compare the coefficients of the exponential function that the authors used to fit the data (they don’t explain why they used that one), they used the Friedman Test. Why? Didn’t the data have a normal distribution? They again used the Tukey-Kramer correction. I think that they should probably have used the Dunn-Bonferroni correction to correct for multiple comparisons done with the Friedman Test.

A. We used Friedman test because the coefficient data had a no normal distribution as highlighted by the Kolmogorov-Smirnov test. This is now specified in the Materials and methods section: “Considering that the coefficient data had not a normal distribution, *a* and *b* coefficients in the different conditions were compared using a Friedman test” A

Bonferroni correction was applied in post-hoc analysis, changing little the results. This is now specified in the text: ".. post hoc tests with Bonferroni correction were employed for pairwise comparisons"
".. the *b* values for the *Synch* condition were significantly higher than those of both the *VisionOnly* and *Asynch* conditions (r = 0.47, z = 2.10, p = 0.034; r = 0.49, z = 2.21, p = 0.005; respectively)."

9. The important significant interaction between time and condition, which is the basis for the post-hoc tests and the author's main claims, was p=0.049. Thus, it is very important that all pre-processing decisions are well motivated and appropriately implemented to ensure that this significant interaction is solid.
A. The new analyses and edits support the robustness of the work. It is worth to say that significant interaction between time and condition factors is the basis for the post-hoc test applied on the mean blood flow data and the claims of our work are partly based on such findings. However, we would like to highlight that we did not just find significantly different flow between test and control derived from such analysis, but also the comparison of the growth rate between conditions, employing the b fitting parameter, showed that the synchronous illusion condition had a significantly faster growth rate than both the other conditions. Moreover, the fitting coefficients positively correlate with most of the widely-validated measures of the illusion.

10. I could not find where the authors describe where the different blocks were counterbalanced, assuming they were. Given that all conditions were associated with a drop in blood flow during the first 10s (although I could not find that the authors mention whether the conditions differed in this respect or not), it seems important that the three conditions should be properly counterbalanced to avoid order effects.
A. The conditions were counterbalanced by evenly distributing the order of the condition across the 20 participants (a complete counterbalanced order across the conditions was not possible, because the number of participants to be involved had to be a multiple of six). We have similar number of conditions for the different ordinal position in our pool of participants (first position in the sequence of performed conditions: 7 VisionOnly, 6 Synch and 7 Asynch; second position: 6 VisionOnly, 7 Synch and 7 Asynch; third position: 7 VisionOnly, 7 Synch and 6 Asynch). This has been reported in the main test: "The order of each condition was evenly distributed across participants in order to decrease the order effect in our findings (first position in the sequence of performed conditions: 7-*VisionOnly*, 6-*Synch* and 7-*Asynch*; second position: 6-*VisionOnly*, 7-*Synch* and 7-*Asynch*; third position: 7-*VisionOnly*, 7-*Synch* and 6-*Asynch*)."

LITERATURE REVIEW AND INTERPRETATION
1. Given the uncertainties discussed in this review, I recommend that the authors modify the conclusions to fit the results better. For example, instead of stating that your results "have demonstrated" I would say that your results "suggest" or that "our preliminary evidence indicates". Moreover, in the discussion, the authors unnecessary talk about how "the blood flow dramatically increased", and that this increase occurred "precisely" in the condition designed to test the effect. However, this study only uses one useful control condition, the timing of the blood flow changes was not predicted, and I do not understand what is so dramatic about the effect? There are many examples in the abstract and discussion where I think the authors overstate their

findings' conclusiveness. I recommend modifying the text to present a more accurate and nuanced description of the results and their interpretation.
A. In order to better address the uncertainties inherent in our results, we revisited the description of them in the text accordingly:

- Abstract: "Conclusions: These findings suggest that modulating the representation of a body part impacts its blood perfusion."
- Plain language summary: "By exploiting brachial artery blood flow recording during the rubber hand illusion paradigm, we observed that modulating the belonging of a body part to the body representation seems to increase its perfusion, through a sympathetic-driven downstream vasodilatation."
- Discussion: "The first element to note is that the adopted experimental process induced a consistent modulation of the blood flow…"
  "While the flow in the VisionOnly condition had a shallower drop (the average flow during Δt_d in VisionOnly is not statistically different from the baseline), and remained higher throughout the experiment, in the Asynch control condition the flow had a more pronounced drop due to brush-stroking and it remained lower throughout the experiment. The only condition in which the blood flow seemed to relatively increase the most after experiencing a drop was the synchronous brush-stroking condition, which was precisely the one designed to test the effect of the fake hand embodiment."
  "On the contrary, Asynch and Synch conditions did not experience different magnitudes of drop. Their significantly different value can only be due to their different effect on the body representation of the hand. This finding gives credit to our hypothesis: the embodiment illusion seems to modulate the blood flow directed towards the tested forearm."
  "To our knowledge, we are the first to show that embodying an artificial limb enhances the blood flow directed to the tested limb."
  "Our results suggest an enhancement in limb blood flow with fake hand embodiment."
  "In conclusion, we observed that the modulation of the sense of limb ownership seems to have an impact on the blood flow directed to that limb."
  "This study is the first to bring preliminary evidence that the update of the perceptual status leading to a change of a limb presence in the body representation is paralleled by an enhancement in the perfusion of the tested limb."

2. In on one of the discussion paragraphs, you talk about "the second-time" interval, but in the most recent version of the manuscript, you have three, right?
A. In the most recent version of the manuscript, the analyzed windows are two. However, in some part of the text we refer to a third-time interval. These are typos, the errors are now corrected: "whereas in the second interval, .." "Compared to *Asynch*, the higher *VisionOnly* value in the second interval .."

3. I think you downplay the negative findings too much when reviewing the literature on temperature changes (discussion). In my opinion, we are currently not sure if the arm's skin temperature changes during the RHI. The de Haan study was quite convincing, I think. There exist other published studies with negative findings that you do not cite. We have measured skin temperature in several pilot studies in our lab and never observed significant and systematic

changes. So how do your results fit with the view that the skin temperature might not change during the RHI? Maybe the authors should also consider this scenario in the text. A. In the discussion section we argue that skin perfusion may be unrelated with the blood flow directed towards the limb because only a small portion of it perfuses the skin. This interpretation does not go against the eventuality that the arm's skin temperature does not show significant and consistent changes during RHI, as some other studies report negative findings. We added a brief part in the text (Discussion) to make it clearer:
"A previous study reported cooling of the RHI tested hand[34], probably caused by a reduced hand skin blood perfusion [68]. How our finding would fit with the previous reported cooling of the RHI tested hand? Firstly, it is worth to say that the cooling effect of the RHI is still matter of debate [35, 36]; secondly, skin perfusion may not be representative of all blood flow directed within the limb."

4. In the discussion, you mention studies that found a down-regulation of somatosensory evoked potentials, but you should probably also cite studies that did not, for example, the well-conducted EEG study by Rao and Kayser 2017 (see also the ECoG findings from S1 hand area published by Guterstam et al 2019 Cerebral Cortex). Also, I do not fully understand how this early modulation of somatosensory evoked responses is believed to be related to the blood flow changes in the arm. Is this discussion not too speculative?
A. We are in accord with the reviewer that the sentence could have been considered too much speculative. We eliminate the reference to the relation between down-regulation of somatosensory evoked potentials and blood flow changes

5. In a paragraph towards the end of the discussion, the authors state that the RHI "lacks objective measures". The authors should rephrase this sentence as it is not a correct description of the literature. Several different objective measures have been proposed and replicated in previous RHI studies. I also think that your statement "the proprioceptive drift is an often dissociated measure" needs to be modified because many previous studies have reported significant differences in proprioceptive drift between the synchronous and asynchronous conditions. Moreover, it is not uncommon to find reports of significant correlations between drift and subjective illusion (e.g., Abdulkarim & Ehrsson 2016; Kalckert & Ehrsson 2014).
A: For what concerns the definition of "objective measures", we agree with the reviewer. We have rephrased the sentence: "However, it has several limitations [79–81]; one is that its outcomes are mostly assessed with subjective measures (i.e. Questionnaires)." We have also rephrased the sentence regarding the interpretation of the lack of correlation of the blood flow with the proprioceptive drift: "Indeed, the increase in the blood flow significantly correlated with all the other employed measures designed to rate the strength of the illusion (RHI index, vividness and prevalence scores), except for proprioceptive drift. A possible interpretation for this result may be that proprioceptive drift, although correlated to the subjective measures [44, 48], does not measure the same aspects of the embodiment process as the questionnaire [43]"

6. The title says, "increases blood flow", and this is also how you interpret the results. But as I wrote above, can we be entirely sure that the blood flow does not decrease in both the synchronous and the asynchronous conditions compared to some relevant baseline, but just so to varying degrees after a certain delay? Maybe "changes blood flow", "time-varying changes in blood flow" or "relative increase in blood flow" would be a safer and more appropriately

cautiously worded title given the data?
A. The title "Embodying an artificial hand increases blood flow to the investigated limb" may be intended as 1 compared to a control condition or 2) compared to the previous flow. In our experiment, both possibilities seem to be true. Indeed, in our work, we found that:

1. The blood flow value for the condition of fake hand embodiment was higher with respect to the control in Δt_2 interval, hence, there is an increase of the blood flow with respect to a no embodiment condition. Moreover, calculating the blood flow value from the drop (ΔF) (figure 5), the fitting curve values for Synch condition resulted higher than for both the control conditions in the last 30s of stimulation.
2. Considering as baseline the signal before the start of stimulation, the flow for Synch condition in Δt_2 is still averagely higher than the baseline (see figure 4 in the manuscript and A1). This excludes the hypothesis that "*the blood flow is actually reduced in both the synchronous and the asynchronous conditions*", given that the flow is instead higher than the baseline in Synch.

For such reason, we prefer not to change the title.

7. You conclude that your study provides "further proof that there is a bidirectional influence between ANS and body ownership". However, this study has not tested bidirectionality, so this conclusion is incorrect. I found this part unnecessarily speculative.
A. Our finding alone does not provide evidence of the bidirectional influence between ANS and body ownership. However, our finding seems to indicate that the modulation of body representation has an impact on the efferent branch of ANS; whereas other studies [16-18] highlighted that the afferent branch of ANS contributes with interoception to the multimodal sensory correlation continuously needed to update our representation of the body. Taken together, these findings seem to suggest a bidirectional influence between ANS and body ownership. We changed the related sentences as follows and highlighted the preliminary nature of the statements: "Our findings seem to indicate that the modulation of body representation has an impact on the efferent branch of the ANS; whereas previous studies demonstrated that afferent branch of ANS contribute with the interoception to the body representation [16-18]. Taken together, these findings provides a further suggestion that there is a bidirectional influence of the ANS on body ownership. Interoception, led by the afferent branch of the ANS, contributes to shape the sense of body ownership and, in turn, this modulation may change the autonomic outflow and becomes manifested through changes of the sudomotor and vasomotor activity."

Competing Interests: No competing interests were disclosed.

Reviewer Report 23 November 2021

https://doi.org/10.21956/openreseurope.15369.r27977

© **2021 Peviani V.** This is an open access peer review report distributed under the terms of the Creative Commons Attribution License, which permits unrestricted use, distribution, and reproduction in any medium, provided the original work is properly cited.

Valeria Peviani
Department of Neuroscience, Max Planck Institute for Empirical Aesthetics, Frankfurt, Germany

I appreciate the authors' effort to further improve the clarity of the paper. However, a couple of sentences sound still misleading to me, suggesting that the drop was present in all conditions ("*This drop was present in all conditions, but it was more evident for Synch and Asynch conditions (Figure 4)"*). However, it seems that this drop reflects a physiological variation of the signal (since it is not attributable to the tactile stimulation). This is an important point to be clarified, given the choice to compare the vision-only condition with the other two conditions in the analysis.

In this regard, I think the baseline correction of the signal, together with the contrast synchronous/asynchronous is sufficient to 'correct' for the effect of vision of the hand. The no-vision condition seems more like an additional baseline, in which physiological variations of the signal are observed, and cannot be associated with local tactile or visual events, nor to variation of body sensations or feeling of ownership. I think that that the comparison between async-no-vision and sync-no-vision is not really justified (or necessary). I would ask the authors to comment on this issue.

I appreciate the authors discussing two limits in their study (absence of ownership measure in no-vision condition and measurement for one hand only). However, I still believe their contribution would substantially increase if these issues were addressed in a second experiment.

Competing Interests: No competing interests were disclosed.

I confirm that I have read this submission and believe that I have an appropriate level of expertise to confirm that it is of an acceptable scientific standard, however I have significant reservations, as outlined above.

Author Response 11 Apr 2022

Marco D'Alonzo, Campus Bio-Medico University of Rome, via Alvaro del Portillo, 5, Rome, Italy

The new revised version of the manuscript entitled "Embodying an artificial hand increases blood flow to the investigated limb" is updated. We wish to thank again the Reviewers for the time spent on our manuscript. We carefully considered the Reviewers' observations and suggestions that helped us to refine the quality of the draft. Point-by-point replies (A) to the Reviewers' comments (number) are provided below, and changes to the text are highlighted by underscore here and in the manuscript. The manuscript has been kindly revised by an English mother-tongue scientist (Dr. Silvia Isabella) who we deeply thank.
We look forward to your response.
Sincerely, on behalf of all co-authors Marco D'Alonzo and Giovanni Di Pino

1. *I appreciate the authors' effort to further improve the clarity of the paper. However, a couple of sentences sound still misleading to me, suggesting that the drop was present in all conditions ("This drop was present in all conditions, but it was more evident for Synch and Asynch conditions (Figure 4)"). However, it seems that this drop reflects a physiological variation of the signal (since*

it is not attributable to the tactile stimulation). This is an important point to be clarified, given the choice to compare the vision-only condition with the other two conditions in the analysis.
A. We would like to thank again Prof Peviani for her useful remarks. The drop in VisionOnly condition was meanly lower, but not significantly different, with respect to the baseline. The sentence, that she highlighted, was changed. The reviewer should also take into account that in the highlighted sentence we describe the behavior of the mean blood flow signal in the figure: "This drop was shallower in *VisionOnly* (Figure 4). In addition, in order to better clarify such aspect, other parts of the main text were changed in Discussion: "Furthermore, looking at the average blood flow dynamics, we found a drop common to all conditions (reaching a value significantly lower than the baseline only for *Synch* and *Asynch* conditions), beginning at the start of the experiment" "While the flow in the *VisionOnly* condition had a shallower drop (the average flow during Δt_d in *VisionOnly* is not statistically different from the baseline),.." It is important to note that, in VisionOnly condition, tactile stimuli were not delivered on the participant's hand and this difference in the protocol with respect to the other two conditions can explain the no-significant difference from the baseline for such condition.

2. In this regard, I think the baseline correction of the signal, together with the contrast synchronous/asynchronous is sufficient to 'correct' for the effect of vision of the hand. The no-vision condition seems more like an additional baseline, in which physiological variations of the signal are observed, and cannot be associated with local tactile or visual events, nor to variation of body sensations or feeling of ownership. I think that the comparison between async-no-vision and sync-no-vision is not really justified (or necessary). I would ask the authors to comment on this issue.
A. After noting that the touch of the brush, independently if synchronous or asynchronous, produced an initial drop of the blood flow, we introduced a VisionOnly condition to disentangle the impact of touch, taking into account that the effect of vision (synchrony) was already assessed by the asynchronous condition. Probably, as the reviewer suggests, introducing a condition with only brush-stroking of the real participant's hand and no vision of the fake hand could be an alternative (No Vision procedure). However, considering that in the RHI literature a control condition without brush-stroking was previously employed[43], we decided to use the VisionOnly condition. In the discussion, we proposed also the No Vision procedure as alternative control condition for future experiments aiming to control for the effect of the real hand touch on its blood flow. "To disentangle the effect of hand touch on blood flow from the effect of embodiment, we could have used either a condition maintaining all the factors but touch or a condition with only touch. In the former, participants should have been instructed to simply look at the fake hand, without receiving any paintbrush stimulation on the real hand (*VisionOnly* condition), while in the latter participant's real hand should have been brush-stroked, without vision or stimulation of the fake hand. We chose the former *VisionOnly* condition, because this was already employed in previous work [43]."

3. I appreciate the authors discussing two limits in their study (absence of ownership measure in no-vision condition and measurement for one hand only). However, I still believe their contribution would substantially increase if these issues were addressed in a second experiment.
A. We agree with the Reviewer that a second experiment would have enriched the study and the strength of our claim. We would like very much to comply with the reviewer's

suggestion and conduct a new experiment. Unfortunately, as already said, this is not in our possibilities because the Doppler ultrasonographic machine we employed for the data collection was part of the University Hospital equipment, and the operator (CA) was a neurologist part of the staff. Since the Covid-19 restrictive measures started, it is no longer possible for us to conduct experiments on healthy subjects within the Hospital, neither we are allowed to move the machine elsewhere. The machine needed for this experiment is one in a hundred because 99% of Doppler machines does not allow continuous reporting and export of the flow, but they measure just discrete intervals. We are well aware that our work has limitations, which maybe would have been improved by further experiments. However, please allow me a more general consideration. Once resolved the issue of an arbitrary division of time windows with a brand new blind analysis, and once partly resolved the absence of cue on embodiment of the supplementary control condition (VisionOnly) and having it acknowledged as a limitation, we respectfully think that our work is worthy to be disseminated. As typically in science, if the design of a study and its statistics are correct, confirming its results and expanding them, e.g. through further control conditions, will be a matter for future studies, which we hope will consider worth investigating this topic more deeply. Publishing our pioneering results is of value also to allow others to be involved in the matter.

Competing Interests: No competing interests were disclosed.

Version 1

Reviewer Report 01 September 2021

https://doi.org/10.21956/openreseurope.14712.r27333

© 2021 Ehrsson H. This is an open access peer review report distributed under the terms of the Creative Commons Attribution License, which permits unrestricted use, distribution, and reproduction in any medium, provided the original work is properly cited.

❌ **Henrik Ehrsson**
Department of Neuroscience, Brain, Body and Self Laboratory, Karolinska Institutet, Solna, Sweden

1. Summary

The study is the first to examine the relation between arm blood flow and body ownership in a multisensory bodily illusion, a digital version of the rubber hand illusion. Although the authors ask an interesting and novel question about the link between body ownership and autonomic nervous system activity, the study has some major methodological problems. These issues could potentially be resolved by conducting a second experiment, providing conclusive evidence.

2. Is the work clearly and accurately presented and does it cite the current literature?

The authors provide a background of their study that is sufficient to understand its rationale. However, the literature on interoception, the autonomic system, and body ownership is complex and results are mixed, and this is not always conveyed to the reader. For example, the fifth paragraph of the introduction "…strong relationship between body representation and interoceptive signals" and "interoceptive sensitivity predicts the malleability of participants' body representation ref 20". Actually, there are several previous studies that have failed to replicate relationships between rubber hand illusion and interoception (Horváth *et al* 2020; Crucianelli *et al*. 2018; Critchely *et al*, 2021) making the relationship between body ownership RHI and interoception somewhat unclear. These, or some of these, studies should probably be cited and a more balanced view presented.

The work is accurately presented overall; however, some parts need clarification (see sections below).

3. Is the study design appropriate and does the work have academic merit?

The study has several major problems. The biggest is that the results only provide partial support for the main conclusion and that the results are explorative in my opinion and it is unclear if they can be replicated. The changes in blood flow occur with a very large delay (about 50 s) compared to the likely onset of the hand ownership illusion (about 10s), and we do not understand why. Of course, it could be a slow physiological response that takes time to get going, but it could also be a random fluctuation in the signal that will not be replicated. The statistical analysis is problematic in this case because the authors arbitrarily divide the data into three periods and then find significant differences only at the last, most delayed period. Noteworthy, in the whole period from 15 to 45 seconds, there is no difference between the synchronous and the asynchronous conditions although the illusion is for sure vividly experienced during this period (Gentile et al 2013; Ehrsson *et al* 2004; Lloyd 2007), which can be seen as an observation that goes against the main conclusions of the study. In my opinion, this first experiment corresponds to an explorative, descriptive approach that is OK to use in a first experiment, but now the authors need to conduct a second experiment to test the specific hypothesis generated from the first experiment of a delayed blood flow change induced by the illusion. As it is now, I would not feel comfortable citing this study; I do not know if the finding is real or merely a statistical false positive or an artifact.

Additional serious problems are that the visual-only condition is not matched for tactile stimulation, and thus difficult to compare with the illusion condition. Moreover, this control condition shows higher blood flow than the synchronous illusion condition in the critical time period of 50 to 100 seconds, which is an observation that speaks against the authors' main conclusion of illusion-induced increases in blood flow. Also, the fitted exponential models for the VisionOnly and the Sync conditions look very similar.

Related to this, the authors do not collect questionnaire data from the VisionOnly condition, so we cannot know for sure the illusion was significantly weaker in this condition (even if it is likely). The authors motivate their decision of not including the questionnaire in the VisionOnly condition because "the most significant Botvinick and Cohen questions focus on being touched by the brush, and they lose meaning if the hand is not touched". But this does not make sense to me because they could remove the referral of touch statements and just analyze the ownership statement; many previous studies have done so.

A further limitation that the authors already discuss is that it would be good to record the blood flow from both arms at the same time to confirm that the observed effect is specific to the limb exposed to the illusion. However, I do not understand what the authors mean by saying that “our experimental setup was not robust enough for that”? Please edit for clarity.

A further methodological point is how it was ensured that the recording device's probe was kept still during the experiments, given that it was held by the experimenter manually throughout the procedure. Could this way of recording have produced any artifacts that potentially influenced the outcome?

4. Are sufficient details of methods and analysis provided to allow replication by others?

The Methods and Results sections provide a sufficient amount of details regarding the procedure and the statistical analyses. However, I do not think the arbitrary division of the data into three time periods is well motivated.

The sample size was not justified well. The authors write that “the number of participants was chosen equal to previous RHI studies”. However, they do not cite any studies. Moreover, basic demographics information of the participants should be reported in the Methods section.

5. Are all the source data underlying the results available to ensure full reproducibility?

Yes.

6. Are the conclusions drawn adequately supported by the results?

Not really. I think you need to run a second experiment to demonstrate that the main finding of a delayed illusion-related increase in blood flow is real. The experiment you have so far is hypothesis-generating. Now you need a hypothesis testing experiment.

Additional control conditions would also be good to have to further strengthen the conclusion that the effect is related to changes in embodiment and not to visuotactile synchrony or asynchrony *per se*. This could be, for example, control conditions with synchronous visuotactile stimulation, but the hand presented in an anatomically implausible position, or the strokes are delivered in different directions (spatial incongruence manipulations) (e.g. Gentile *et al* 2013).

References
1. Horváth Á, Ferentzi E, Bogdány T, Szolcsányi T, et al.: Proprioception but not cardiac interoception is related to the rubber hand illusion.*Cortex*. **132**: 361-373 PubMed Abstract | Publisher Full Text
2. Crucianelli L, Krahé C, Jenkinson PM, Fotopoulou AK: Interoceptive ingredients of body ownership: Affective touch and cardiac awareness in the rubber hand illusion.*Cortex*. **104**: 180-192 PubMed Abstract | Publisher Full Text
3. Critchley HD, Botan V, Ward J: Absence of reliable physiological signature of illusory body ownership revealed by fine-grained autonomic measurement during the rubber hand illusion.*PLoS One*. 2021; **16** (4): e0237282 PubMed Abstract | Publisher Full Text
4. Gentile G, Guterstam A, Brozzoli C, Ehrsson HH: Disintegration of multisensory signals from the

real hand reduces default limb self-attribution: an fMRI study.*J Neurosci*. 2013; **33** (33): 13350-66 PubMed Abstract | Publisher Full Text
5. Ehrsson HH, Spence C, Passingham RE: That's my hand! Activity in premotor cortex reflects feeling of ownership of a limb.*Science*. 2004; **305** (5685): 875-7 PubMed Abstract | Publisher Full Text
6. Lloyd DM: Spatial limits on referred touch to an alien limb may reflect boundaries of visuo-tactile peripersonal space surrounding the hand.*Brain Cogn*. 2007; **64** (1): 104-9 PubMed Abstract | Publisher Full Text

Is the work clearly and accurately presented and does it cite the current literature?
Partly

Is the study design appropriate and does the work have academic merit?
Partly

Are sufficient details of methods and analysis provided to allow replication by others?
Yes

If applicable, is the statistical analysis and its interpretation appropriate?
Partly

Are all the source data underlying the results available to ensure full reproducibility?
Yes

Are the conclusions drawn adequately supported by the results?
Partly

Competing Interests: No competing interests were disclosed.

Reviewer Expertise: Cognitive neuroscience, body representation research, and bodily illusions.

I confirm that I have read this submission and believe that I have an appropriate level of expertise to state that I do not consider it to be of an acceptable scientific standard, for reasons outlined above.

Author Response 26 Oct 2021

Marco D'Alonzo, Campus Bio-Medico University of Rome, via Alvaro del Portillo, 5, Rome, Italy

The revised version of the manuscript entitled “Embodying an artificial hand increases blood flow to the investigated limb” is updated. We wish to thank the Reviewers for the time spent on our manuscript. We are really pleased with the interest toward our work. We carefully considered the Reviewers’ observations and suggestions that helped us to refine the quality of the draft. Point-by-point replies to the Reviewers’ comments (in *italics*) are provided below, and changes to the text are highlighted in red here and in the manuscript. We look forward to your response. Sincerely, on behalf of all co-authors Marco D’Alonzo and

Giovanni Di Pino.

The study is the first to examine the relation between arm blood flow and body ownership in a multisensory bodily illusion, a digital version of the rubber hand illusion. Although the authors ask an interesting and novel question about the link between body ownership and autonomic nervous system activity, the study has some major methodological problems. These issues could potentially be resolved by conducting a second experiment, providing conclusive evidence.

Authors' Response: Dear Prof. Ehrsson, we are really honored that one of the worldwide most expert in this topic devoted his precious time to read our paper and suggest ways to improve it. We took into account all your inputs and where we could we have tried to implement them as much as possible. We modified the text in line with your suggestions. Unfortunately, at the present, we are not in the possibility to replicate the experiment for the reasons explained below. However, we have re-analyzed our data from the scratch, deleting the previous arbitrary division so much criticized and we have also added the analysis of the VisionOnly proprioceptive drift, suggesting no embodiment in this condition, which we previously decided not to include in the manuscript. This work costs big effort and we deeply trust on the reliability of the results we found. We hope that in its present version it will be worth of your trust and approval.

The authors provide a background of their study that is sufficient to understand its rationale. However, the literature on interoception, the autonomic system, and body ownership is complex and results are mixed, and this is not always conveyed to the reader. For example, the fifth paragraph of the introduction "...strong relationship between body representation and interoceptive signals" and "interoceptive sensitivity predicts the malleability of participants' body representation ref 20". Actually, there are several previous studies that have failed to replicate relationships between rubber hand illusion and interoception (Horváth et al 2020; Crucianelli et al. 2018; Critchely et al, 2021) making the relationship between body ownership RHI and interoception somewhat unclear. These, or some of these, studies should probably be cited and a more balanced view presented.

Authors' Response: We trust on the impact of ANS on body representation, thus we may have been biased in the presenting the introduction in favor of this hypothesis. Thank you for having make us realize this. Accordingly with your suggestion, the introduction has been rewritten to present a more balanced view:
"Moreover, ANS homeostatic information related to pain, temperature, pH, carbon dioxide, and oxygen are sent to the insula and interact with somatosensory processing. This has been suggested to have a role in the construction of the body representation [2]." "Evidence of the tight connection between the ANS and central body representation may be derived from complex regional pain syndrome (CRPS) [7]" "Beside pathological models, in healthy subject the meaning and strength of the relationship between body representation and interoceptive signals is still matter of debate. For example, emerging evidence suggests interoceptive information such as cardiac feedback to modulate the visual body perception [16] and influence one's own body awareness [17, 18] or, vice-versa, changes in body-ownership and self-identification to alter the ability to detect internal body signals [19]. Furthermore, interoceptive sensitivity seems to predict the malleability of participants' body representation [20]." Moreover, an additional paragraph was inserted in the introduction: "On

the other hand, the relationship between ownership generated by the RHI paradigm and these interoceptive measures was not always confirmed [80] and other interoceptive indexes were found not correlated to the strength of the illusion during the RHI paradigm: e.g. the capability of participants in heartbeat counting tasks [81, 82]."

The changes in blood flow occur with a very large delay (about 50 s) compared to the likely onset of the hand ownership illusion (about 10s), and we do not understand why. Of course, it could be a slow physiological response that takes time to get going, but it could also be a random fluctuation in the signal that will not be replicated.

Authors' Response: "it could also be a random fluctuation in the signal that will not be replicated" Considering that the reviewer asked about the replicability of the results, we performed an additional analysis in order to check how much our results are robust and replicable among the different participants (i.e. whether the obtained results could be due to peculiar behavior of few outlier participants). The difference in mean blood flow between conditions in each participant was calculated in the intervals of interest, and the distribution of these data was plotted (attached figure 2R: Δt_1 = [5 - 31s] data in the left side and Δt_2 = [69 - 100s] data in the right side). All the resulting distributions were found normal by using Kolmogorov Smirnoff test, demonstrating that the distribution of the differences was not skewed. Focusing on the most interesting distribution of data (i.e. the difference in blood flow value between Synch and Asynch conditions in the second interval of interest, in the ellipse in figure 2R), a higher value of Synch condition with respect to Asynch one was highlighted for main part of participants (15 out of 20 had difference between Synch and Asynch higher than 0, mean and median values of the distribution were also higher than 0), additionally, no outliers were present. This demonstrate a consistent behavior (homogeneity of the sample) among the participants, showing a tendency to have higher Synch value with respect to the Asynch one, and demonstrating the replicability of our results.

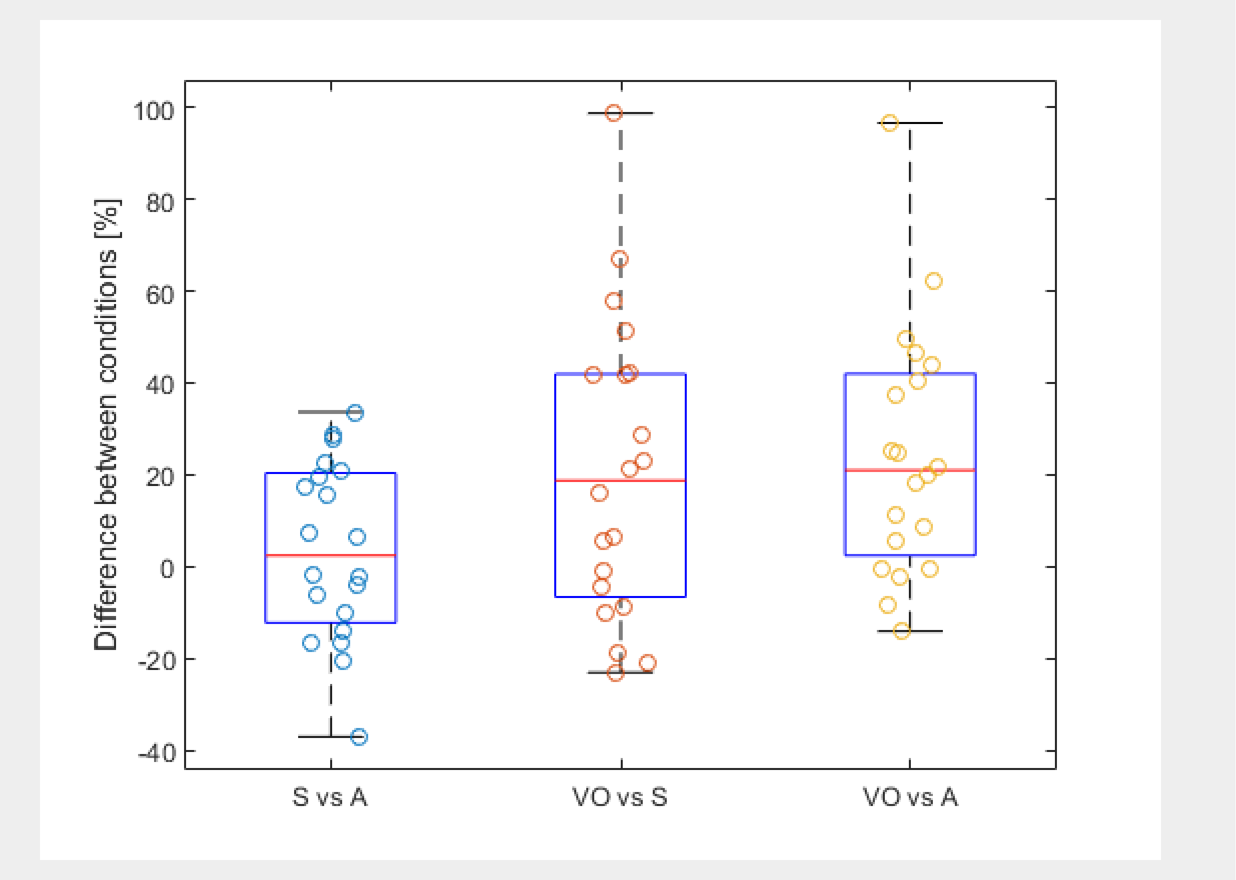
100
80
60
40
20
0
-20
-40
Difference between conditions [%]
S vs A
VO vs S
VO vs A

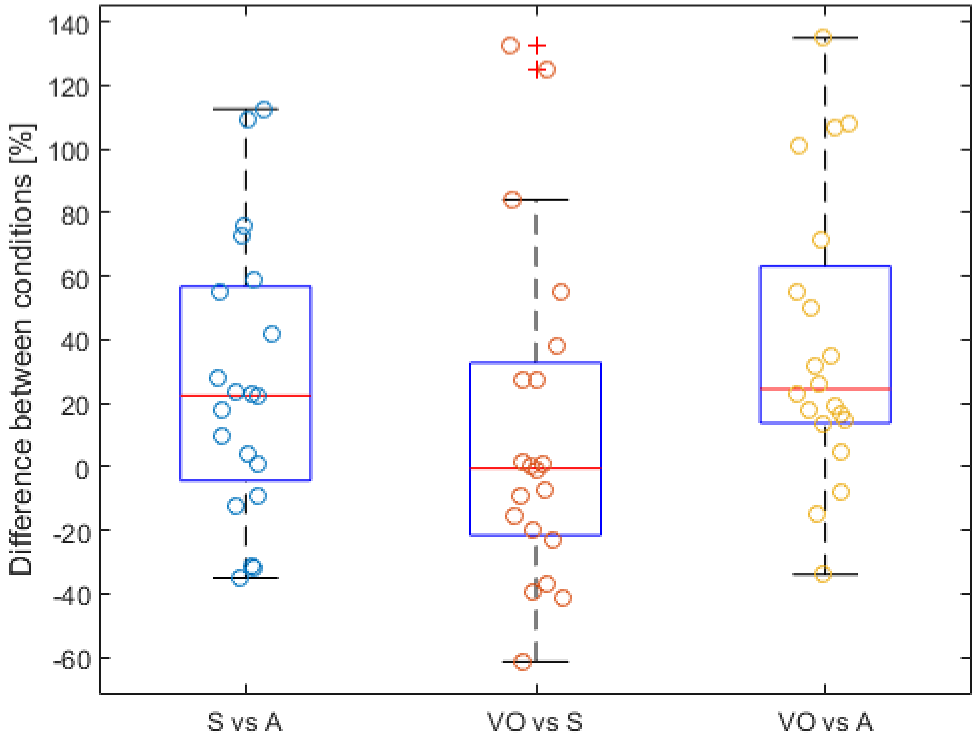

Figure 2R: Difference in blood flow value between conditions in the time-intervals of interest (Δt1, left; Δt2, right; S, Synch; A, Asynch; VO, VisionOnly). Box and whisker plots: median (red lines), 1st and 3rd quartiles (box), lowest and highest values comprised within 1.5 times the interquartile range from the 1st and 3rd percentiles (whisker).

The statistical analysis is problematic in this case because the authors arbitrarily divide the data into three periods and then find significant differences only at the last, most delayed period. Noteworthy, in the whole period from 15 to 45 seconds, there is no difference between the synchronous and the asynchronous conditions although the illusion is for sure vividly experienced during this period (Gentile et al 2013; Ehrsson et al 2004; Lloyd 2007), which can be seen as an observation that goes against the main conclusions of the study.

Authors' Response: In the new version, the statistical analysis has been re-run without the criticized a priori division of time periods (please see the answers below). We agree with the reviewer that from 15 to 45 seconds the illusion is already present. We suppose that after the initial drop, the flow needs 70 seconds to reach significantly different average flow value because of the slow time course of the signal. However, please consider that the growth rate of the Synch signal becomes different from the Asynch already at 25s and it becomes different from VisioOnly at about 50s (Figure 5).

This was highlighted in Results section:
"After this drop, the *F(t)* tends to increase in all conditions. In particular, the mean blood

flow behavior for *Synch* condition starts to have a higher growth than *Asynch* already after 25 s, and higher than *VisionOnly* after about 48 s." To overcame the arbitrary division in time windows, we decided to make a new analysis to identify the intervals where perform the statistics. We compute a point-by-point ANOVA using multiple datasets and a permutation testing to find the cluster of significant difference among the three conditions. In particular, we employed 250 permutations in *clustersize-based permutation testing* and *percentile of mean cluster sum* as method to define the threshold distinguishing between "significant" and "non-significant" clusters. We found two different "significant" clusters one in the interval between 5 and 31 s and the other between 69 and 100 s. In this way, we focused our analysis only on these two intervals. "In order to identify the time intervals where perform the statistics, we computed a point-by-point ANOVA using multiple datasets and a permutation testing to find the cluster of significant difference among the three conditions. In particular, we employed 250 permutations in clustersize-based permutation testing and percentile of mean cluster sum as method to define the threshold distinguishing between "significant" and "non-significant" clusters. We found two different "significant" clusters: one in the interval between 5 and 31 s (Δt_1) and the other between 69 and 100 s (Δt_2). We focused our analysis only on these two intervals."

In my opinion, this first experiment corresponds to an explorative, descriptive approach that is OK to use in a first experiment, but now the authors need to conduct a second experiment to test the specific hypothesis generated from the first experiment of a delayed blood flow change induced by the illusion.

Authors' Response: As regard as the request of a second experiment, please see the last reply (below).

Additional serious problems are that the visual-only condition is not matched for tactile stimulation, and thus difficult to compare with the illusion condition.

Authors' Response: In principle, we wanted to test just synchronous vs asynchronous condition. When we performed preliminary recordings to test the setup, we noted that the touch of the brush, independently if synchronous or asynchronous, produced an initial drop of the blood flow. When multiple factors determine an effect, to control for the contribution of one of those factors either a control condition with only that factor or a control condition without that factor can be used. Since our experimental question was not linked to the change of flow induced by the touch, to be able to isolate the searched effect, theoretically we had two possibilities: either i) introducing a condition with only brush-stroking, which however would have left several open questions (related to how managing visual feedback of the real and rubber hand), or, ii) on the contrary, introducing a condition not affected by touch. Considering that in the RHI literature a control condition without brush-stroking was previously and largely employed, we decided to proceed with the latter option and use the VisionOnly condition. In few words, having a condition without touch was done purposely to isolate the effect of our enquire from the unwanted mere effect of touch.

This is clearly stated in the text. "Since we suspected that brush-stroking itself could have affected the flow independently from the achieved embodiment, a third condition was introduced as further control, where participants were instructed to simply look at the fake

hand, without receiving any paintbrush stimulation on the real or on the fake hand (*VisionOnly* condition). In the latter case, tactile stimuli were not present." And "Such condition was performed in order to control for the effect of mere tactile stimulation on the blood flow and was considered as an additional condition of no embodiment [36]."

Moreover, this control condition shows higher blood flow than the synchronous illusion condition in the critical time period of 50 to 100 seconds, which is an observation that speaks against the authors' main conclusion of illusion-induced increases in blood flow.

Authors' Response: This is true only before correcting for the drop (new Figure 4), because VisionOnly was not affected (or only marginally), by the initial drop. Once we corrected for the initial drop (Figure 5) from around 35 sec to the end VisionOnly (black line) is below the Synch condition (blue line). Thus, we do not see this observation speaking against our main conclusion.

Also, the fitted exponential models for the VisionOnly and the Sync conditions look very similar.

Authors' Response: When we corrected the blood flow for the initial drop and modelled it with an exponential curve we tested two parameters, a connected to the average value and b, which was the slope of the curve, i.e. the growth rate. Actually, the growth rate (the b values) of the Synch was significantly higher than VisionOnly (as well as higher than Asynch), while there was no significant difference between VisionOnly and Asynch, which means that there was a similar growth dynamics for these two conditions. Please see Figure 5. However, it is true that the blood flow curves between VisionOnly and Synch condition are closer in terms of mean values, even when the drop was subtracted. We tried to speculate on the reason of it, suggesting two possible hypotheses: i) the higher VisionOnly value in the second interval may be the effect of the previous milder VisionOnly drop; ii) it may be the effect of the concomitant absence of stroking of the real hidden hand. This speculation has been added to the text   It is likely that, in case of VisionOnly condition, the absence of stroking on the hidden hand during the session could have affected the blood flow value and caused a higher increase in the blood flow with respect to the case of the asynchronous condition. This was reported in the main text: "Compared to *Asynch*, the higher *VisionOnly* value in the second interval may be the effect of the previous milder *VisionOnly* drop or due to the absence of stroking on the hidden hand during the session."

Related to this, the authors do not collect questionnaire data from the VisionOnly condition, so we cannot know for sure the illusion was significantly weaker in this condition (even if it is likely). The authors motivate their decision of not including the questionnaire in the VisionOnly condition because "the most significant Botvinick and Cohen questions focus on being touched by the brush, and they lose meaning if the hand is not touched". But this does not make sense to me because they could remove the referral of touch statements and just analyze the ownership statement; many previous studies have done so.

Authors' Response: The issue of original Botvinick and Cohen questions losing meaning if the hand was not touched has already been raised by Rhode *et al* (2011), the first authors to test this condition (in Rhode *et al* (2011), the ownership questionnaire was not recorded for such condition).

As the Reviewer suggests, we could change the list of the questionnaire, by deleting the questions relative to the touch. However, either we should have deleted those items for all conditions (losing an important part of the illusion outcome also for Synch and Asynch), or we should have tested a different number of items in different comparisons. We thought the latter solution would have messed the experimental design. We collected the proprioceptive drift, however not having the questionnaire, for homogeneity and for maintaining simpler the study design, we originally decided not to analyze and include it in the manuscript. These data were included now: "Such condition was performed in order to control for the effect of mere tactile stimulation on the blood flow and was considered as an additional condition of no embodiment [36] . As in previous studies [36], questionnaire outcomes were not recorded in this condition." A posteriori, we agree with the Reviewer that not having collected the questionnaire in VisionOnly was a bad choice. Considering Reviewer's suggestion, we have now analyzed VisionOnly proprioceptive drift and we found it not significantly different from Asynch and significantly lower than Synch (new Figure 3), supporting the claim of no embodiment for VisionOnly, as much as concerning this measure. We hope that this may help to ease Reviewer's concern about the absence of cue on VisionOnly embodiment. Nevertheless, we cannot hide that the absence of the questionnaire in this condition is a limitation of our design. Indeed, this is clearly acknowledged in the discussion section of the manuscript (see below). RHI is a model with several constraints, of which we are aware and which we accept each time we gather any insight from it. Considering that the main control condition was the Asynch, and that VisionOnly was introduced only to test the effect of the absence of touch on the initial drop, while testing VisionOnly embodiment was not part of the scope of the paper, we strongly think that, once acknowledged, this limitation is not enough to preclude the publication of our results. . "Indeed, despite embodiment illusion being strongly dependent on the integration of coherent multisensory afferences, previous studies hypothesized the mere vision of a fake hand placed in a congruent position as being able to induce some mild degree of embodiment [44, 45], while another study did not [46]. Being the proprioceptive drift significantly lower in *VisionOnly* than in *Synch,* and similar to *Asynch*, *VisionOnly* induced illusion should be very low. Unfortunately, we cannot take a conclusive position on this possibility, because we did not collect questionnaire in *VisionOnly* for two reasons: i) this condition was introduced to control for the cause of the initial flow drop while testing its embodiment was not its original scope; ii) as previously raised [36], several very important items of Botvinick and Cohen questionnaire focus on being touched by the brush, and they lose meaning if the hand is not touched. We collected proprioceptive drift, but considering that this measure is related to different embodiment aspects than the questionnaire [36], the absence of questionnaires in the *VisionOnly* condition should be considered a limitation of the study and it is envisaged for future works investigating the topic."

A further limitation that the authors already discuss is that it would be good to record the blood flow from both arms at the same time to confirm that the observed effect is specific to the limb exposed to the illusion. However, I do not understand what the authors mean by saying that "our experimental setup was not robust enough for that"? Please edit for clarity.

Authors' Response: We did not record the blood flow in the non-tested limb. The flow should have been recorded at the same time in the two limbs. We had already tried to

record at the same time in the two limbs, but results were not trustable with the experimental setup we implemented, because for a single experimenter was not feasible to hold still two probes and accurately monitor the blood flow on the two arms. Blood flow recording should be done by an ultrasonography expert, being it very operator-dependent, and the only involved author with this skill was Dr Altamura (this skill is confirmed by her publication record).

This was better explained in the text: “we realized that our experimental setup was not robust enough for that, because for a single experimenter it was not feasible to hold still two probes and accurately monitor the blood flow on the two arms.”

A further methodological point is how it was ensured that the recording device's probe was kept still during the experiments, given that it was held by the experimenter manually throughout the procedure. Could this way of recording have produced any artifacts that potentially influenced the outcome?

Authors’ Response: Up today, most of neurophysiology experiments still relays on stimulation, recording and perturbation performed manually by experimenters. For instance, let’s think to all TMS experiments where the coil is manually held upon the designed hotspot by the experimenter. For such reason, especially when the task is really operator-dependent, the only caution we can have is to rely on experimenter expertise.

In our case, the experimenter devoted to maintaining still the probe was a Neurologist expert in ultrasonography, thus in measuring the blood flow using the doppler device who perform on average twenty clinical exams per day, this was better specified in Methods section: “During the whole protocol, the probe was kept still by a second experimenter (CA) expert in ultrasonography.” This was a further reason which did not allowed to record two arms simultaneously. Thus, it is unlikely that blood flow recording was affected by artifact; however, even if any artifact was produced, this should be present in each condition, considering that the way how the experimenter maintaining the probe did not change among conditions. Additionally, the experimenter maintaining the probe was not confident with the RHI protocol and she was not in the position to see the real hand brushing performed by the other experimenter. Thus, she was blind about the stimulation condition, unable to bias blood flow recording. This was added to the Methods. “The second experimenter maintaining the probe was not confident with the RHI protocol and was not in the position to see the real hand brushing performed by the other experimenter.”

The Methods and Results sections provide a sufficient amount of details regarding the procedure and the statistical analyses. However, I do not think the arbitrary division of the data into three time periods is well motivated.

Authors’ Response: We had a reason to divide the three periods, however we are sorry if we were not able to explain it, thus making our choice to look arbitrary. In order to avoid any doubt, we decided to re-run a brand new analysis to identify blindly the intervals of interest where statistics was then performed. For such reason, we compute a point-by-point ANOVA using multiple datasets and a permutation testing to find the cluster of significant difference among the three conditions. In particular, we employed 250 permutations in

clustersize-based permutation testing and *percentile of mean cluster sum* as method to define the threshold distinguishing between “significant” and “non-significant” clusters. We found two different “significant” clusters one in the interval between 5 and 31 s and the other between 69 and 100 s. Then, we focused our analysis only on these two intervals, where we found the same results previously found with the three time periods a priori chosen.

The sample size was not justified well. The authors write that “the number of participants was chosen equal to previous RHI studies”. However, they do not cite any studies. Moreover, basic demographics information of the participants should be reported in the Methods section.

Authors’ Response: We are really sorry for the missing information. The references relative to the sample size were added in the sentence: “the number of participants was chosen equal to previous RHI studies [30, 32, 34, 36, 84, 85].”

References Armel KC, Ramachandran VS. Projecting sensations to external objects: evidence from skin conductance response. Proc Biol Sci. 2003;270(1523):1499-506. Abdulkarim Z, Ehrsson HH. No causal link between changes in hand position sense and feeling of limb ownership in the rubber hand illusion. Attention, Perception, & Psychophysics. 2016;78(2):707-20. D’Alonzo M, Mioli A, Formica D, Vollero L, Di Pino G. Different level of virtualization of sight and touch produces the uncanny valley of avatar’s hand embodiment. Scientific reports. 2019;9(1):1-11. Rohde M, Di Luca M, Ernst MO. The rubber hand illusion: feeling of ownership and proprioceptive drift do not go hand in hand. PloS one. 2011;6(6):e21659. Kalckert A, Ehrsson HH. The moving rubber hand illusion revisited: Comparing movements and visuotactile stimulation to induce illusory ownership. Consciousness and cognition. 2014;26:117-32. Peviani V, Magnani F, Ciricugno A, Vecchi T, Bottini G. Rubber hand illusion survives ventral premotor area inhibition: a rTMS study. Neuropsychologia. 2018;120:18-24.

As regard the suggestion to include basic demographic information in Methods, we have to say that, originally, such info “Twenty volunteers took part in the experiment (age: 29.55 ± 6.12; 12 M, 8 F; 20 right-handed as by self-report)” was indeed placed on the Methods section. Then, the information was placed in Results following the suggestions of the editorial team of Open Research Europe. However, the number of participants is also reported now in Methods section: “Twenty participants were selected among a population..”

I think you need to run a second experiment to demonstrate that the main finding of a delayed illusion-related increase in blood flow is real. The experiment you have so far is hypothesis-generating. Now you need a hypothesis testing experiment.

Additional control conditions would also be good to have to further strengthen the conclusion that the effect is related to changes in embodiment and not to visuotactile synchrony or asynchrony per se. This could be, for example, control conditions with synchronous visuotactile stimulation, but the hand presented in an anatomically implausible position, or the strokes are delivered in different directions (spatial incongruence manipulations) (e.g. Gentile et al 2013).

Authors’ Response: We agree with the Reviewer that additional control conditions would have enriched the study and the strength of our claim. We would like very much to comply

with reviewer's suggestion and conduct a new experiment. Unfortunately, this is not in our possibilities because the Doppler ultrasonographic machine we employed for the data collection was part of the University Hospital equipment, and the operator (CA) was a neurologist part of the staff. Since the Covid-19 restrictive measures started, it is no longer possible for us to conduct experiments on healthy subjects within the Hospital, neither we are allowed to move the machine elsewhere. The machine needed for this experiment is one in a hundred because 99% of Doppler machines does not allow continuous reporting and export of the flow, but they measure just discrete intervals. We are well-aware that our work has limitations, which maybe would have been improved by further experiments.

However, please allow me a more general consideration. Once resolved the issue of an arbitrary division of time windows with a brand new blind analysis, and once partly resolved the absence of cue on embodiment of the supplementary control condition (VisionOnly) and having it acknowledged as limitation we can affirm that our study design is sound. Moreover, study limitations are clearly stated in the manuscript (Discussion):

"the absence of questionnaires in the *VisionOnly* condition should be considered a limitation of the study and it is envisaged for future works investigating the topic." "This is the first study demonstrating that the update of the perceptual status leading to a change of a limb presence in the body representation is paralleled by an enhancement in the perfusion of the tested limb. It also opens the intriguing question of whether the reported changes are unspecific effects of an alert response regarding the whole body or, on the contrary, are specifically causally and topographically related to the limb, the representation of which was modulated. We speculated on this topic providing cues in favor of the latter. This, however, remains an extremely interesting question, a matter still open for future research."
Our sample size is suited to our conclusions. More importantly, we did not just find significantly different flow between test and two controls, but also the correlation of such differences with embodiment measures. I'm pretty sure the Reviewer is aware of how much this is rare when working with heterogeneous healthy participants and mixing physiological and behavioral outcomes. Considering all those points, we respectfully think that our work is worth to be disseminated. As typically in science, if the design of a study and its statistics are correct, confirming its results and expanding them, e.g. through further control conditions, will be matter for future studies, which we hope will consider worth to investigate this topic more deeply. Publishing our pioneering results is worth also to allow others to be involved in the matter.

The aspects highlighted by Prof. Ehrsson were reported in the discussion: "Additionally, to assess the repeatability of our findings, further studies could be performed to assess the difference of synchronous brush-stroking with additional control conditions, such as with the fake hand placed in an incongruent position. In such way, it will be possible to confirm whether the found effect is related to changes in embodiment and not to manipulation of visuotactile stimulation synchrony."

Competing Interests: No competing interests were disclosed.

Reviewer Report 09 August 2021

https://doi.org/10.21956/openreseurope.14712.r27284

© **2021 Peviani V.** This is an open access peer review report distributed under the terms of the Creative Commons Attribution License, which permits unrestricted use, distribution, and reproduction in any medium, provided the original work is properly cited.

Valeria Peviani
Department of Neuroscience, Max Planck Institute for Empirical Aesthetics, Frankfurt, Germany

This work investigates changes in blood flow of the arm during the Rubber Hand Illusion, a well-known procedure that induces the sense of ownership towards a fake hand, by triggering a multimodal mismatch. Participants' blood flow was measured in two conditions of visuo-tactile stimulation (fake hand and unseen participant's hand were stroked simultaneously [synchronous condition], fake hand and unseen participant's hand were stroked non simultaneously [asynchronous condition], and one control condition (same set-up, but no tactile stimulation).

The manuscript is well-written and clear (but see some questions below); results are clearly reported and described by figures. I think the authors raise a very relevant question, and this work could potentially contribute to our knowledge on physiological correlates of transitory abnormal experiences in the context of body ownership.

However, I have major concerns on the experimental design and data analysis, detailed below. In my view, these issues, which undermine the reliability of results and their interpretation, need to be carefully addressed before approval.

I hope my comments and suggestions will be useful to the authors to improve the quality of their work.

Major

1. In the introduction, the authors well describe evidence on the role of interoceptive signals in body perception and awareness. I think it would be also relevant to briefly describe studies that investigated physiological changes possibly related to ANS activity (e.g., skin conductance, temperature) during the RHI and other multisensory illusions.

   Relatedly, I think this paper may be very relevant when introducing the work and discussing results: Teaford, M., Fitzpatrick, J., & James Smart Jr, L. (2021). The impact of experimentally induced limb ischemia on the rubber hand illusion. Perception, 50(1), 88-96.

2. *"A drop of the mean blood flow values was identified at around 10s from the beginning of the conditions. Thus, we corrected the blood curves of all the three conditions to make all of them starting from the same value after the drop, the value of the drop was subtracted to the mean blood flow by using the following equation: ..."*

   If I correctly understood, the authors re-aligned the signals for the curve-fitting and coefficient analysis. What was the precise extent of the re-alignment (in time) for each

condition?

3. *"This suggests that the drop was due to the initial, mostly unexpected, tactile stimulation of the hidden hand caused by the brush, regardless of whether the stroke was synchronous or asynchronous and if an illusion was achieved"*

   "[the vision-only] condition was performed in order to control for the effect of mere tactile stimulation on the blood flow and was considered as an additional condition of no embodiment"

   From these sentences, it reads that the signal drop results from tactile stimulation, and that the vision-only condition was included to control for the effect of tactile stimulation. However, no tactile stimulation was delivered in the vision-only condition, which makes it not suitable to account for signal changes due to mere touch, such as signal changes due to non-repeated tactile stimulation.

   Relatedly, as the authors acknowledged in the discussion, the signal increase in the vision-only condition may be due to increased sense of body ownership. However, in this paradigm, the vision-only condition is not an effective no-embodiment condition, especially since no subjective reports on the feeling of ownership towards the fake hand were collected.

   In this regard, authors state: *"There are both technical and scientific reasons that may suggest not recording embodiment measures in VisionOnly: the most significant Botvinick and Cohen questions focus on being touched by the brush, and they lose meaning if the hand is not touched."*

   In my view, existent ownership questionnaires could be adapted to the experimental set-up, as it has been done in other studies (e.g., for virtual set-ups: Tieri *et al.*, 2017; for mirror-box: Medina *et al.*, 2015).

4. Analyses and discussion seem to rely on the assumption that the signal drop described in the signal in the vision-only condition is due to tactile stimulation, despite no tactile stimulation was delivered in this condition. In my view, this is a critical issue that needs to be carefully addressed.

   How do the authors justify the presence of a drop in the vision-only condition, and which is the criterion used to establish the presence of this drop? How did the authors establish that the drop in the vision-only condition does not reflect a physiological oscillation of the signal? Was this drop systematically present in the sample?

   I would ask the authors to comment on this, revise their analyses and discussion accordingly, and consider collecting further evidence.

   In light of observations reported in comments 3 and 4, I think that this work would substantially improve by including more suitable control condition(s) in a follow-up experiment. The present design poses important limits to the interpretation of results. The vision-only condition does not effectively control for neither touch, since no tactile stimulus was delivered, nor no-embodiment, since no ownership measures were collected.

5. The present investigation does not allow to establish the specificity of the effect, i.e., whether the effect is specific to the arm involved in the RHI, thus related to the feeling of ownership towards it, or represent a more diffuse physiological change associated to the multisensory illusion.

   While the authors put forward indirect evidence for the local specificity of the observed effect in the discussion, this could be clarified by including blood flow measures for the homologous arm or even another body part. This would significantly improve the impact of this work.

6. Order effects are relevant in the context of RHI experiments. Why was the order of conditions randomized and not counterbalanced?

7. *"Considering that the dynamic of the blood flow oscillation at frequencies of 0.02–0.05 Hz are mainly affected by sympathetic nerve activity, we chose to analyze the blood flow signal by splitting the recording session into three time intervals (33s each)."*

   This sentence is not very clear to me. Can the authors further elaborate on the choice of splitting the signal into three intervals?

8. As the authors stated, blood flow dynamics are relatively slow, in the range of 2-to-5 cycles per 100 seconds. In light of this, can the authors justify the choice of a 5-seconds baseline?

Minor

1. Introduction: "*Emerging evidence for the existence of a strong relationship between body representation and interoceptive signals are not confined to pathological models*". I think grammar needs to be adjusted.

2. Method: *"Therefore, in this case, the number of participants was chosen equal to previous RHI studies."* Can the authors cite the studies on which their sample size estimation rely on?

3. Method: I suggest the authors to report the sample size in the participants section as well.

4. Method, Experimental Procedure: I would suggest the authors to clarify how references 33 and 34 relate to the sentence: *"They could see the content of each compartment only when the experimenter turned the relative internal light on"*

5. Method, Results (pg. 8): *"Considering the interaction between ... were identified in the second interval"* Were these contrasts corrected for multiple comparisons?

6. It is not clear which *alfa* and *beta* coefficients were included in the correlation analysis whose results are reported in Table 2. I guess those belonging of the synchronous condition, but I would suggest the authors make it explicit.

7. Figure 2: axis label and units are missing in top panel.

References
1. Medina J, Khurana P, Coslett HB: The influence of embodiment on multisensory integration using the mirror box illusion.*Conscious Cogn*. 2015; **37**: 71-82 PubMed Abstract | Publisher Full Text
2. Teaford M, Fitzpatrick J, James Smart L: The Impact of Experimentally Induced Limb Ischemia on the Rubber Hand Illusion.*Perception*. 2021; **50** (1): 88-96 PubMed Abstract | Publisher Full Text
3. Tieri G, Gioia A, Scandola M, Pavone EF, et al.: Visual appearance of a virtual upper limb modulates the temperature of the real hand: a thermal imaging study in Immersive Virtual Reality. *Eur J Neurosci*. **45** (9): 1141-1151 PubMed Abstract | Publisher Full Text

Is the work clearly and accurately presented and does it cite the current literature?
Partly

Is the study design appropriate and does the work have academic merit?
Partly

Are sufficient details of methods and analysis provided to allow replication by others?
Yes

If applicable, is the statistical analysis and its interpretation appropriate?
Partly

Are all the source data underlying the results available to ensure full reproducibility?
Yes

Are the conclusions drawn adequately supported by the results?
Partly

Competing Interests: No competing interests were disclosed.

Reviewer Expertise: Body perception and representation

I confirm that I have read this submission and believe that I have an appropriate level of expertise to state that I do not consider it to be of an acceptable scientific standard, for reasons outlined above.

Author Response 26 Oct 2021

Marco D'Alonzo, Campus Bio-Medico University of Rome, via Alvaro del Portillo, 5, Rome, Italy

The revised version of the manuscript entitled “Embodying an artificial hand increases blood flow to the investigated limb” is updated. We wish to thank the Reviewers for the time spent on our manuscript. We are really pleased with the interest toward our work. We carefully considered the Reviewers’ observations and suggestions that helped us to refine the quality of the draft. Point-by-point replies to the Reviewers’ comments (in *italics*) are provided below, and changes to the text are highlighted in red here and in the manuscript. We look forward to your response. Sincerely, on behalf of all co-authors Marco D’Alonzo and

Giovanni Di Pino

This work investigates changes in blood flow of the arm during the Rubber Hand Illusion, a well-known procedure that induces the sense of ownership towards a fake hand, by triggering a multimodal mismatch. Participants' blood flow was measured in two conditions of visuo-tactile stimulation (fake hand and unseen participant's hand were stroked simultaneously [synchronous condition], fake hand and unseen participant's hand were stroked non simultaneously [asynchronous condition], and one control condition (same set-up, but no tactile stimulation).

The manuscript is well-written and clear (but see some questions below); results are clearly reported and described by figures. I think the authors raise a very relevant question, and this work could potentially contribute to our knowledge on physiological correlates of transitory abnormal experiences in the context of body ownership.

However, I have major concerns on the experimental design and data analysis, detailed below. In my view, these issues, which undermine the reliability of results and their interpretation, need to be carefully addressed before approval.

I hope my comments and suggestions will be useful to the authors to improve the quality of their work.

Authors' Response: We really thank Prof Peviani for her extremely useful remarks, which allowed us to strength the manuscript. We deeply trust that in its new form it reached the needed level of clearness to deserve her approval.

In the introduction, the authors well describe evidence on the role of interoceptive signals in body perception and awareness. I think it would be also relevant to briefly describe studies that investigated physiological changes possibly related to ANS activity (e.g., skin conductance, temperature) during the RHI and other multisensory illusions.

Relatedly, I think this paper may be very relevant when introducing the work and discussing results: Teaford, M., Fitzpatrick, J., & James Smart Jr, L. (2021). The impact of experimentally induced limb ischemia on the rubber hand illusion. Perception, 50(1), 88-96.

Authors' Response: Thanks for your suggestion. We followed it integrating the suggested reference and now the introduction seems a lot better balanced. The introduction was edited following the suggestion of the Reviewer:
"A link between autonomic mechanisms and cognitive processes behind body representation has been previously demonstrated using RHI paradigm, such as the altered temperature regulation while inducing body ownership over the fake hand [20 79]. The occurrence of the RHI results in a disownership and a decrease of the skin temperature of the real hand [54], but the consistency of such finding is still under debate [58, 59]. Furthermore, increased fluctuations in the skin conductance have been proven to correlate with the onset and the strength of the illusion during the RHI [50]. On the other hand, the relationship between ownership generated by the RHI paradigm and these interoceptive measures was not always confirmed [80] and other interoceptive indexes were found not correlated to the strength of the illusion during the RHI paradigm: e.g. the capability of participants in

heartbeat counting tasks [81, 82]. Interestingly, artificially-induced peripheral ischemia modulated the proprioceptive drift during the RHI paradigm [83]."

"A drop of the mean blood flow values was identified at around 10s from the beginning of the conditions. Thus, we corrected the blood curves of all the three conditions to make all of them starting from the same value after the drop, the value of the drop was subtracted to the mean blood flow by using the following equation: ..." *If I correctly understood, the authors re-aligned the signals for the curve-fitting and coefficient analysis. What was the precise extent of the re-alignment (in time) for each condition?*

Authors' Response: We did not realign the signals in time (along the x-axis), but only in amplitude of the flow (dependent variable). All the data was treated with the same procedure of analysis to focus on the flow behaviour beyond the initial drop and to make the results comparable among the three conditions. To make all the curves starting from a common ground and eliminate the offset, we subtract the relative drop (the mean amplitude of the blood flow on the interval between 5 and 15 s after the beginning of the condition) to each condition. We are sorry if this was not clear enough. In the new version Methods have been modified to make this clear:

"we corrected the blood curves (relative shift along the y-axis) of all the three conditions to make all of them starting from the same flow value after the drop. This was done by subtracting the value of the drop to the mean blood flow as in the following equation:

Δ Ft=*Ft*-*F*Δ td $\Delta F(t) = F(t) - \bar{F}(\Delta t_d)$

(2) where *F*

$\bar{F}$ (Δt_d) (i.e. the drop of the signal) was calculated as blood flow value averaged on a 10s window centered 10s after the beginning of the trial (i.e. Δt_d = [5s, 15s])... The obtained signal in the interval between 10s and 100s were fitted by using an exponential curve"

"This suggests that the drop was due to the initial, mostly unexpected, tactile stimulation of the hidden hand caused by the brush, regardless of whether the stroke was synchronous or asynchronous and if an illusion was achieved"

"[the vision-only] condition was performed in order to control for the effect of mere tactile stimulation on the blood flow and was considered as an additional condition of no embodiment" *From these sentences, it reads that the signal drop results from tactile stimulation, and that the vision-only condition was included to control for the effect of tactile stimulation. However, no tactile stimulation was delivered in the vision-only condition, which makes it not suitable to account for signal changes due to mere touch, such as signal changes due to non-repeated tactile stimulation.*

Authors' Response: In principle, we wanted to test just synchronous vs asynchronous condition. When we performed preliminary recordings to test the setup, we noted that the touch of the brush, independently if synchronous or asynchronous, produced an initial drop of the blood flow. When multiple factors determine an effect, to control for the contribution

of one of those factors either a control condition with only that factor or a control condition without that factor can be used. Since our experimental question was not linked to the change of flow induced by the touch, to be able to isolate the searched effect, theoretically we had two possibilities: either i) introducing a condition with only brush-stroking, which however would have left several open questions (related to how managing visual feedback of the real and rubber hand), or, ii) on the contrary, introducing a condition not affected by touch. Considering that in the RHI literature a control condition without brush-stroking was previously employed, we decided to proceed with the latter option and use the VisionOnly condition. In few words, having a condition without touch was done purposely to isolate the effect of our enquire from the unwanted mere effect of touch. Regarding the suitability to use VisioOnly to control for the impact of touch on the drop, please see also our reply to your comment n 6 (below). We hope that this explication makes things clearer.

This is clearly stated in the text: "Since we suspected that brush-stroking itself could have affected the flow independently from the achieved embodiment, a third condition was introduced as further control, where participants were instructed to simply look at the fake hand, without receiving any paintbrush stimulation on the real or on the fake hand (*VisionOnly* condition). In the latter case, tactile stimuli were not present." and "Such condition was performed in order to control for the effect of mere tactile stimulation on the blood flow and was considered as an additional condition of no embodiment [36]."

Relatedly, as the authors acknowledged in the discussion, the signal increase in the vision-only condition may be due to increased sense of body ownership. However, in this paradigm, the vision-only condition is not an effective no-embodiment condition, especially since no subjective reports on the feeling of ownership towards the fake hand were collected. In this regard, authors state: ***"There are both technical and scientific reasons that may suggest not recording embodiment measures in VisionOnly: the most significant Botvinick and Cohen questions focus on being touched by the brush, and they lose meaning if the hand is not touched."***

In my view, existent ownership questionnaires could be adapted to the experimental set-up, as it has been done in other studies (e.g., for virtual set-ups: Tieri et al., 2017; for mirror-box: Medina et al., 2015).

Authors' Response: The issue of original Botvinick and Cohen questions losing meaning if the hand was not touched has already been raised by Rhode *et al* (2011), the first authors to test this condition (in Rhode *et al* (2011), the ownership questionnaire was not recorded for such condition).

As the Reviewer suggest, we could change the list of the questionnaire, by deleting the questions relative to the touch. However, either we should have deleted those items for all conditions (losing an important part of the illusion outcome also for Synch and Asynch), or we should have tested a different number of items in different comparisons. We thought the latter solution would have messed the experimental design. We collected the proprioceptive drift, however not having the questionnaire, for homogeneity and for maintaining simpler the study design, we originally decided not to analyze and include it in the manuscript. Now these data were included: "Such condition was performed in order to control for the effect of mere tactile stimulation on the blood flow and was considered as an

additional condition of no embodiment [36] . As in previous studies [36], questionnaire outcomes were not recorded in this condition."

A posteriori, we agree with the Reviewer that not having collected the questionnaire in VisionOnly was a bad choice. Considering Reviewer's suggestion, we have now analyzed VisionOnly proprioceptive drift and we found it not significantly different from Asynch and significantly lower than Synch (new Figure 3), supporting the claim of no embodiment for VisionOnly, as much as concerning this measure. We hope that this may help to ease Reviewer's concern about the absence of cue on VisionOnly embodiment. Nevertheless, we cannot hide that the absence of the questionnaire in this condition is a limitation of our design. Indeed, this is clearly acknowledged in the discussion section of the manuscript (see below). RHI is a model with several constraints, of which we are aware and which we accept each time we gather any insight from it. Considering that the main control condition was the Asynch, and that VisionOnly was introduced only to test the effect of the absence of touch on the initial drop, while testing VisionOnly embodiment was not part of the scope of the paper, we strongly think that, once acknowledged, this limitation is not enough to preclude the publication of our results. "Indeed, despite embodiment illusion being strongly dependent on the integration of coherent multisensory afferences, previous studies hypothesized the mere vision of a fake hand placed in a congruent position as being able to induce some mild degree of embodiment [44, 45], while another study did not [46]. Being the proprioceptive drift significantly lower in *VisionOnly* than in *Synch,* and similar to *Asynch*, *VisionOnly* induced illusion should be very low. Unfortunately, we cannot take a conclusive position on this possibility, because we did not collect questionnaire in *VisionOnly* for two reasons: i) this condition was introduced to control for the cause of the initial flow drop while testing its embodiment was not its original scope; ii) as previously raised [36], several very important items of Botvinick and Cohen questionnaire focus on being touched by the brush, and they lose meaning if the hand is not touched. We collected proprioceptive drift, but considering that this measure is related to different embodiment aspects than the questionnaire [36], the absence of questionnaires in the *VisionOnly* condition should be considered a limitation of the study and it is envisaged for future works investigating the topic."

Analyses and discussion seem to rely on the assumption that the signal drop described in the signal in the vision-only condition is due to tactile stimulation, despite no tactile stimulation was delivered in this condition. In my view, this is a critical issue that needs to be carefully addressed.

How do the authors justify the presence of a drop in the vision-only condition, and which is the criterion used to establish the presence of this drop? How did the authors establish that the drop in the vision-only condition does not reflect a physiological oscillation of the signal? Was this drop systematically present in the sample?

I would ask the authors to comment on this, revise their analyses and discussion accordingly, and consider collecting further evidence.

In light of observations reported in comments 3 and 4, I think that this work would substantially improve by including more suitable control condition(s) in a follow-up experiment. The present design poses important limits to the interpretation of results. The vision-only condition does not

effectively control for neither touch, since no tactile stimulus was delivered, nor no-embodiment, since no ownership measures were collected.

Authors' Response: On this point, respectfully, it seems to us that the Reviewer misunderstood the message of our work (or we were not enough clear).
VisionOnly had no tactile stimulation, neither it had drop (or really negligible compared to Synch and Asynch). This was the reason why we attributed the initial drop to tactile stimulation. The drop (and its absence) can be easily seen by visually inspecting the signals, which clearly show a consistent decrease for all but the VisionOnly condition after about 10 s from the beginning of the stimulation (new Figure 4).

Which is the criterion used to establish the presence of this drop?

Authors' Response: We firstly defined what we meant with drop: "where $\bar{F}$ *(Δt_d)* (i.e. the drop of the signal) was calculated as blood flow value averaged on a 10s window centered 10s after the beginning of the trial (i.e. Δt_d = [5s, 15s])." Reviewer: "How did the authors establish that the drop in the vision-only condition does not reflect a physiological oscillation of the signal?" We do think that the drop in VisionOnly was mainly the physiological oscillation of the signal. Indeed, the values of the drop were (mean ± st. dev.): -18.4 ± 18.3% and -24.8 ± 16.4% for, Synch and Asynch condition respectively, while just -3.4 ± 16.9%, for VisionOnly.

Was this drop systematically present in the sample?

Authors' Response: How much the drop was systematically present in the participant sample can be gathered from the standard error, which was also graphically represented by the blur of the signal in the new Figure 4 (see zoom in Figure 1R). Then, we run a statistical analysis on the drop values: the distribution of drop values was tested against 0 value (i.e. baseline) by using one sample t-test. Only drop values in Synch and Asynch conditions were statistically lower than the baseline, supporting our hypothesis that only in those condition we have a significant drop, thus linking it to the presence of touch in the Synch and Asynch conditions. These edits were reported in Methods section: "For all condition, the signal drop value was analyzed to assess whether it was significantly lower than the baseline (i.e. 0 value), by using a one-sample t-test." And in Results section: "In particular, focusing on the drop values calculated as blood flow value averaged on Δt_d interval, we found that only drop values in *Synch* and *Asynch* conditions were statistically lower than the baseline (*Synch*: $t(19) = -4.52$, $p < 0.001$; *Asynch*: $t(19) = -6.78$, $p <0.001$). The values of the drop were (mean ± st. dev.): -18.4 ± 18.3% and -24.8 ± 16.4% for, *Synch* and *Asynch* condition respectively, while just -3.4 ± 16.9%, for *VisionOnly*.." However, even if a lot less represented, the Reviewer is right when she says that a subtle decrease of the signal was also present in VisionOnly, peaking at about the same time (10 sec), which is however not different from the normal fluctuation of the blood signal (black line of new Figure 4). We discussed this mild decrease suggesting that, beyond the touch-dependent strong decrease of the flow seen in Synch and Asynch, also other factors other than the touch of the hidden hand, such as the sudden lighting of the RHI platform compartment, likely have produced the flow decrease.

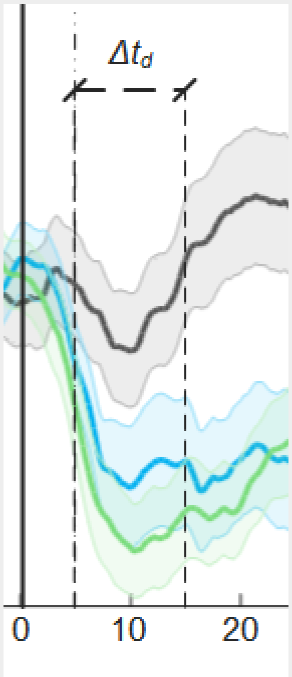

Figure 1R: zoom of Figure 4: Mean blood flow (F) averaged across participants for each condition, the shade represents the standard error (SEM), dashed lines indicate the time intervals where the drop values was calculated (Δt_d).

The present investigation does not allow to establish the specificity of the effect, i.e., whether the effect is specific to the arm involved in the RHI, thus related to the feeling of ownership towards it, or represent a more diffuse physiological change associated to the multisensory illusion.

While the authors put forward indirect evidence for the local specificity of the observed effect in the discussion, this could be clarified by including blood flow measures for the homologous arm or even another body part. This would significantly improve the impact of this work.

Authors' Response: We did not record the blood flow in the non-tested limb. The flow should have been recorded at the same time in the two limbs. We had already tried to record at the same time in the two limbs, but results were not trustable with the experimental setup we implemented, because it was impossible for a single experimenter to hold two probes and accurately monitor the blood flow on the two arms at the same time. Blood flow recording should be done by an ultrasonography expert, being it very operator-dependent, and the only involved author with this skill was Dr Altamura (this skill is confirmed by her publication record).

We are well-aware of this limitation of our study, which we clearly discussed on the manuscript: "In the attempt to test the local specificity of our hypothesis, in a preliminary experiment run before the study, we tried to record the blood flow from both arms at the same time, but unfortunately we realized that our experimental setup was not robust enough for that, because for a single experimenter it was not feasible to hold still two probes and accurately monitor the blood flow on the two arms."

Once established that we were not able to record the flow of the two upper limbs simultaneously, we could have recorded them in different sessions. We agree on that, but unfortunately, we have no the possibility to refine this work with new acquisition. Indeed, the Doppler ultrasonography machine we employed for the data collection was part of the University Hospital equipment, and the operator (CA) is a neurologist part of the staff. Since the Covid-19 restrictive measures started, it is no longer possible for us to conduct experiments on healthy subjects within the Hospital, neither are we allowed to move the machine elsewhere. The machine needed for this experiment is peculiar because 99% of Doppler machines do not allow continuous reporting and export of the flow, but they measure just discrete intervals. To reduce the impact of this limitation, we calculated the resistance index, from which an indirect cue on the local specificity of the autonomic response can be gathered. We found a decrease of the index, sign of vasodilatation, and a negative correlation with flow, suggesting a peripheral vessel origin of the change in the blood flow.

We are well-aware that our work has limitations, which maybe would have been improved by further experiments, as already highlighted in Discussion section: “It also opens the intriguing question of whether the reported changes are unspecific effects of an alert response regarding the whole body or, on the contrary, are specifically causally and topographically related to the limb, the representation of which was modulated. We speculated on this topic providing cues in favor of the latter. This, however, remains an extremely interesting question, a matter still open for future research.” However, our study design is sound, its limitations are clearly stated, our sample size is suited to our conclusions, and we did not just find significantly different flow between test and two controls, but also the correlation of such differences with embodiment measures. Considering all those points, we respectfully think that our work is worth to be disseminated. As typically in science, confirming the results and expanding them, e.g. through further control conditions, will be matter for future studies, which we hope will consider worth to investigate this topic more deeply. Publishing our pioneering results is worth also to allow others to be involved in the matter. We hope that being not in the possibility to acquire new data for a period lasting unpredictably, once the limitations have been clearly acknowledged and the other raised points cleared, Prof Peviani will be in favor to disseminate the results we have gathered.

Order effects are relevant in the context of RHI experiments. Why was the order of conditions randomized and not counterbalanced?

Authors’ Response: Thank you for having raised this point which allowed us to be more detailed in the Methods section. The conditions cannot be completely counterbalanced with 20 participants (in order to have complete counterbalanced order among the conditions, the number of participants to be involved had to be a multiple of six); however, we try to have similar number of conditions for the different ordinal position in our pool of participants (first position in the sequence of performed conditions: 7 VisionOnly, 7 Synch and 6 Asynch; second position: 6 VisionOnly, 7 Synch and 7 Asynch; third position: 7 VisionOnly, 6 Synch and 7 Asynch). This has been now reported in the main test:
“Similar number of conditions for the different ordinal position in experimental sequence

was obtained in our pool of participants (first position in the sequence of performed conditions: 7 *VisionOnly*, 7 *Synch* and 6 *Asynch*; second position: 6 *VisionOnly*, 7 *Synch* and 7 *Asynch*; third position: 7 *VisionOnly*, 6 *Synch* and 7 *Asynch*)."

"Considering that the dynamic of the blood flow oscillation at frequencies of 0.02–0.05 Hz are mainly affected by sympathetic nerve activity, we chose to analyze the blood flow signal by splitting the recording session into three time intervals (33s each)."
This sentence is not very clear to me. Can the authors further elaborate on the choice of splitting the signal into three intervals?

Authors' Response: We had a reason to divide the three periods, however we are sorry if we were not able to explain it, thus making our choice to look arbitrary. In order to avoid any doubt, we decided to re-run a brand new analysis to identify blindly the intervals of interest where statistics was then performed. For such reason, we compute a point-by-point ANOVA using multiple datasets and a permutation testing to find the cluster of significant difference among the three conditions. In particular, we employed 250 permutations in *clustersize-based permutation testing* and *percentile of mean cluster sum* as method to define the threshold distinguishing between "significant" and "non-significant" clusters. We found two different "significant" clusters one in the interval between 5 and 31 s and the other between 69 and 100 s. Then, we focused our further analysis only on these two intervals, where we found the same results previously found with the three time periods a priori chosen.

We changed the text accordingly: "In order to identify the time intervals where perform the statistics, we computed a point-by-point ANOVA using multiple datasets and a permutation testing to find the cluster of significant difference among the three conditions. In particular, we employed 250 permutations in clustersize-based permutation testing and percentile of mean cluster sum as method to define the threshold distinguishing between "significant" and "non-significant" clusters. We found two different "significant" clusters: one in the interval between 5 and 31 s (Δt_1) and the other between 69 and 100 s (Δt_2). We focused our analysis only on these two intervals."

As the authors stated, blood flow dynamics are relatively slow, in the range of 2-to-5 cycles per 100 seconds. In light of this, can the authors justify the choice of a 5-seconds baseline?

Authors' Response: We started to acquire blood flow 20 seconds before the light was turned on. Before starting to consider the signal suited for baseline, we wanted to be sure that the signal had time to be stable. From visual inspection, we estimated that 5 out of 20 seconds was the best compromise between stabilization after starting the acquisition and stability of the signal before starting the experiment.

*Introduction: "**Emerging evidence for the existence of a strong relationship between body representation and interoceptive signals are not confined to pathological models**". I think grammar needs to be adjusted.*

Authors' Response: Thank you for noting it, the sentence, after the revision, was re-written: "Beside pathological models, in healthy subject the meaning and strength of the

relationship between body representation and interoceptive signals is still matter of debate. For example, emerging evidence suggests interoceptive information such as cardiac feedback to modulate the visual body perception [16] and influence one's own body awareness [17, 18] or, vice-versa, changes in body-ownership and self-identification to alter the ability to detect internal body signals [19]. Furthermore, interoceptive sensitivity seems to predict the malleability of participants' body representation [20]."

Method: ***"Therefore, in this case, the number of participants was chosen equal to previous RHI studies."*** *Can the authors cite the studies on which their sample size estimation rely on?*

Authors' Response: Sorry for the missing citations. The references were added in the sentence: "the number of participants was chosen equal to previous RHI studies [30, 32, 34, 36, 84, 85]."

References Armel KC, Ramachandran VS. Projecting sensations to external objects: evidence from skin conductance response. Proc Biol Sci. 2003;270(1523):1499-506. Abdulkarim Z, Ehrsson HH. No causal link between changes in hand position sense and feeling of limb ownership in the rubber hand illusion. Attention, Perception, & Psychophysics. 2016;78(2):707-20. D'Alonzo M, Mioli A, Formica D, Vollero L, Di Pino G. Different level of virtualization of sight and touch produces the uncanny valley of avatar's hand embodiment. Scientific reports. 2019;9(1):1-11. Rohde M, Di Luca M, Ernst MO. The rubber hand illusion: feeling of ownership and proprioceptive drift do not go hand in hand. PloS one. 2011;6(6):e21659. Kalckert A, Ehrsson HH. The moving rubber hand illusion revisited: Comparing movements and visuotactile stimulation to induce illusory ownership. Consciousness and cognition. 2014;26:117-32. Peviani V, Magnani F, Ciricugno A, Vecchi T, Bottini G. Rubber hand illusion survives ventral premotor area inhibition: a rTMS study. Neuropsychologia. 2018;120:18-24.

Method: I suggest the authors to report the sample size in the participants section as well.

Authors' Response: The sample size was reported also in Participants section. "Twenty participants were selected among a population.."

Method, Experimental Procedure: I would suggest the authors to clarify how references 33 and 34 relate to the sentence: ***"They could see the content of each compartment only when the experimenter turned the relative internal light on"***

Authors' Response: Sorry, this was a typo, the references were shifted in the previous paragraph, considering that the cited references refer to the experimental setup employed here.
"Participants were placed in front of a custom-made experimental set-up, made of three parallel compartments (L x W x H = 40 x 60 x 20 cm each) covered by a two-way mirror (Figure 1) [33, 34]."

Method, Results (pg. 8): ***"Considering the interaction between … were identified in the second interval"*** *Were these contrasts corrected for multiple comparisons?*

Authors' Response: Yes, the contrast for the two previously identified time windows between conditions were corrected for multiple comparison. In particular the Tukey-Kramer adjustment was employed, as was stated in the Methods:
"Hence, a paired t-test with Tukey-Kramer adjustment was employed as post-hoc analysis."
".. Considering the interaction between the factors and given that our aim was to find a difference among conditions in the single time interval, we made two separate post-hoc analysis using a Tukey-Kramer adjustment, one for each time-interval."

It is not clear which alfa and beta coefficients were included in the correlation analysis whose results are reported in Table 2. I guess those belonging of the synchronous condition, but I would suggest the authors make it explicit.

Authors' Response: In order to calculate the correlation between the fitting coefficients and illusion outcomes both the Synch and Asynch conditions, data were employed, pooling them together. This was reported in Methods section, and now better explicated:
"The link between blood flow changes and embodiment was investigated by correlating (Spearman's) *a* and *b* coefficients with the illusion outcomes in *Synch* and *Asynch* condition pooled together."   "Table 2: Correlation values between fitting coefficients and illusion outcomes calculated pooling together *Synch* and *Asynch* conditions."

Figure 2: axis label and units are missing in top panel.

Authors' Response: Thank you for noting it, the figure was corrected.

Competing Interests: No competing interests were disclosed.
